# Multi-omics analysis reveals the genetic basis of rice fragrance mediated by *betaine aldehyde dehydrogenase 2*

**DOI:** 10.1016/j.jare.2021.12.004

**Published:** 2021-12-18

**Authors:** Rungnapa Phitaktansakul, Kyu-Won Kim, Kyaw Myo Aung, Thant Zin Maung, Myeong-Hyeon Min, Aueangporn Somsri, Wondo Lee, Sang-Beom Lee, Jungrye Nam, Seung-Hyun Kim, Joohyun Lee, Soon-Wook Kwon, Bhagwat Nawade, Sang-Ho Chu, Sang-Won Park, Kwon Kyoo Kang, Yoo-Hyun Cho, Young-Sang Lee, Ill-Min Chung, Yong-Jin Park

**Affiliations:** a Department of Plant Resources, College of Industrial Sciences, Kongju National University, Yesan 32439, Republic of Korea; b Center of Crop Breeding on Omics and Artificial Intelligence, Kongju National University, Yesan 32439, Republic of Korea; c Seedpia, 85 Maesil-ro, Kwonsun-ku, Suwon 16395, Republic of Korea; d Department of Applied Bioscience, Konkuk University, Seoul 05029, Republic of Korea; e Department of Plant Bioscience, Pusan National University, Pusan 46241, Republic of Korea; f Chemical Safety Division, National Institute of Agriculture Science (NIAS), Wanju 55365, Republic of Korea; g Department of Horticultural Life Science, Hankyong National University, Anseong 17579, Republic of Korea; h Department of Medical Biotechnology, Soonchunhyang University, Asan 31538, Republic of Korea

**Keywords:** Betaine aldehyde dehydrogenase 2, Fragrant rice, Haplotype, Expression quantitative trait loci, Protein quantitative trait loci, Volatiles

## Abstract

**Introduction:**

Fragrance is an important economic and quality trait in rice. The trait is controlled by the recessive gene *betaine aldehyde dehydrogenase 2* (*BADH2*) via the production of 2-acetyl-1-pyrroline (2AP).

**Objectives:**

Variation in *BADH2* was evaluated at the population, genetic, transcriptional, and metabolic levels to obtain insights into fragrance regulation in rice.

**Methods:**

Whole-genome resequencing of the Korean World Rice Collection of 475 rice accessions, including 421 breeding lines and 54 wild accessions, was performed. Transcriptome analyses of a subset of 279 accessions, proteome analyses of 64 accessions, and volatile profiling of 421 breeding lines were also performed.

**Results:**

We identified over 3.1 million high-quality single nucleotide polymorphisms (SNPs) in Korean rice collection. Most SNPs were present in intergenic regions (79%), and 190,148 SNPs (6%) were located in the coding sequence, of which 53% were nonsynonymous. In total, 38 haplotypes were identified in the *BADH2* coding region, including four novel haplotypes (one in cultivated and three in wild accessions). Tajima's D values suggested that *BADH2* was under balancing selection in japonica rice*.* Furthermore, we identified 316 expression quantitative trait loci (eQTL), including 185 *cis*-eQTLs and 131 *trans*-eQTLs, involved in *BADH2* regulation. A protein quantitative trait loci (pQTL) analysis revealed the presence of *trans*-pQTLs; 13 pQTLs were mapped 1 Mbp from the *BADH2* region. Based on variable importance in projection (VIP) scores, 15 volatile compounds, including 2AP, discriminated haplotypes and were potential biomarkers for rice fragrance.

**Conclusion:**

We generated a catalog of haplotypes based on a resequencing analysis of a large number of rice accessions. eQTLs and pQTLs associated with *BADH2* gene expression and protein accumulation are likely involved in the regulation of 2AP variation in fragrant rice. These data improve our understanding of fragrance and provide valuable information for rice breeding.

## Highlights

• A set of novel functional haplotypes were detected in the *BADH2* coding region
• Tajima’s D index suggested balancing selection in japonica rice for *BADH2*
• 316 expression quantitative trait loci (eQTLs) regulate *BADH2* expression
• 13 *trans*-protein quantitative trait loci (pQTLs) were mapped on different chromosomes
• 15 volatiles discriminated fragrant haplotypes in PLS-DA model and VIP score

## Graphical abstract

**Figure:**
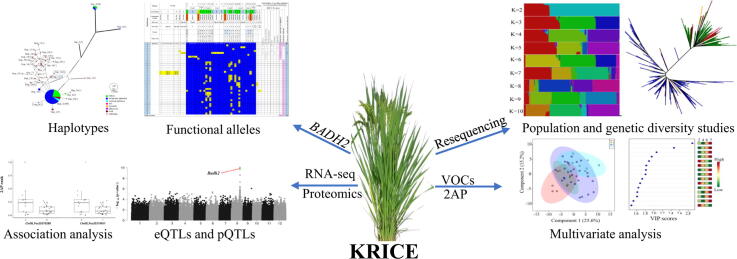

## Introduction

Rice taste is directly related to its fragrance [1] and therefore fragrant rice is widely preferred among consumers. Fragrant rice accounts for approximately 15–18% of the world’s rice trade, reflecting high demand [2], [3]. Rice breeders have paid particular attention to the molecular and biochemical basis of fragrance and its improvement [4], [5].

Previous studies have identified hundreds of volatile compounds in fragrant rice, among which 2-acetyl-1-pyrroline (2AP) has been identified as a key compound responsible for a popcorn-like fragrance [6], [7], [8], [9]. Further, studies have confirmed that rice fragrance results from the accumulation of 2AP as a consequence of the recessive *betaine aldehyde dehydrogenase* 2 (*BADH2*) gene [6], [10]. The gene is located on chromosome 8, comprising 15 exons and 14 introns and encoding 503 amino acids [10], [11], [12]**.** Active BADH2 stimulates γ-aminobutyric acid (GABA) synthesis and inhibits 2AP accumulation in non-fragrant rice [12]. Fragrant rice with mutations in *BADH2* fails to convert γ-aminobutyraldehyde (GABAld) into GABA, resulting in GABAld accumulation. Consequently GABAld spontaneously cyclizes to Δ1-pyrroline, leading to the synthesis of 2AP [10], [12]. A gene expression analysis has revealed that reduced expression levels of *BADH2* and glyceraldehyde-3-phosphate dehydrogenase (*GAPDH*) and elevated transcript levels of triose phosphate isomerase (*TPI*) and Δ**1**-pyrroline-5-carboxylic acid synthetase (*P5CS*) increase 2AP accumulation [13], [14] (Fig. S1).

Various *BADH2* mutations associated with fragrance in rice have been reported [11]. In particular, an 8 bp deletion in exon 7 and a 7 bp deletion in exon 2 are frequently detected in many fragrant rice accessions [15], [16]. Moreover, the strength of the fragrance differs even in presence of mutant alleles and variation in 2AP accumulation has been recorded in several fragrant varieties from South and South-East Asia, as well as the USA [7], [17], [18], [19], [20]. This underlines the possibility that regulation at the transcriptional and translational levels influences rice fragrance along with *BADH2* allelic variation.

With advances in high-throughput sequencing technologies, -omics-based studies of rice have progressed considerably, including analyses of genomes (genomics), RNA transcripts (transcriptomics), and metabolites (metabolomics) aimed at unraveling the functional variants and molecular mechanisms underlying traits for crop improvement [21]. Expression quantitative trait loci (eQTLs) and protein quantitative trait loci (pQTL) provide insight into the genetic basis of transcriptional variation and protein abundance, respectively [22], [23]. These strategies are used extensively in human genetics to identify functionally relevant genetic variants associated with phenotypic variation and to reduce the gap between genomic variation, expression levels, and phenotype variation. However, integrative studies of rice fragrance linking transcript levels to phenotypic variation are still lacking. Here, we evaluate fragrance polymorphism in a Korean rice collection at the population, gene, transcript, and proteomic levels.

We used multiple -omics approaches to study *BADH2* variation, and fragrance regulation. We conducted whole-genome resequencing of 475 accessions, including 54 wild rice accessions. Furthermore, we quantified *BADH2* expression in a subset of 279 accessions by RNA-Seq, protein levels in a subset of 64 accessions by liquid chromatography-tandem mass spectrometry (LC-MS/MS), and the volatile profiles in a subset of 421 accessions by HS-solid-phase microextraction (SPME)/GC–MS. By this gene-to-metabolite approach, we identified four novel alleles in the *BADH2* coding region, 316 eQTLs, and 13 pQTLs. Considering the importance of fragrance in the rice market, our data are expected to provide important information for rice breeding.

## Material and methods

### Plant materials for whole-genome resequencing

A set of 137 accessions were previously selected from 25,604 rice accessions of the Korean Genebank of the Rural Development Administration (RDA) using PowerCore [24], [25]. In addition, 284 varieties from RDA genebank and 54 wild rice accessions procured from the International Rice Research Institute (IRRI) were added to the collection to build a core set of 475 accessions (**Table S1**). This Korean World Rice Collection (475 accessions) was comprised of 421 cultivated accessions, including 305 japonica, 102 indica, 9 aus, 2 aromatic, and 3 admixture varieties.

### Resequencing of 475 rice accessions

Genomic DNA of each accession was extracted from the leaf using the DNeasy Plant Mini Kit (Qiagen, Germantown, MD, USA) and subjected to whole-genome resequencing using the Illumina HiSeq 2500 sequencing platform (Illumina Inc., San Diego, CA, USA), with an average coverage of approximately 15×. The raw sequences were aligned to the Nipponbare rice reference genome (International Rice Genome Sequencing Project IRGSP-1.0; http://rapdb.dna.affrc.go.jp/download/irgsp1.html) using Burrows-Wheeler Aligner (BWA) version 0.7.8 with default parameters [26]. Sequence quality was checked based on the alignments using Integrative Genomics Viewer (IGV) with default parameters. The duplicate reads in the raw resequencing data were removed using PICARD version 2.14 and Samtools version 1.8. The Genome Analysis Toolkit (GATK) version 3.6 pipeline was used for SNP calling [27]. Whole-genome sequencing data from 3,000 rice accessions [28] were also evaluated using PowerCore [24].

### Population structure analysis

SNPs (minor allele frequency [MAF] ≥ 5% and missing rate ≤ 20%) detected in the whole-genome resequencing data were used to a construct phylogenetic tree and were evaluated by a principal component analysis (PCA). FigTree version 1.4.3 (http://tree.bio.ed.ac.uk/software/figtree/) was used to construct a dendrogram based on the neighbor-joining method with 1,000 bootstrap replications. The PCA was conducted using the high-quality SNPs in TASSEL 5.0 and plots were drawn using the R package ggplot2 version 3.6.2 [29]. Population structure was evaluated using the fastStructure [30] with *K* values ranging from 2 to 10. Nucleotide diversity (π), population differentiation (*F_ST_*), and Tajima’s *D* values were calculated using VCFtools 0.1.13 with 500 bp sliding windows and 500 steps.

### Analysis of *BADH2* genetic variation

Genetic variation in the *BADH2* gene was evaluated using the DNA variant VCF file containing the whole-genome resequencing data. Plots of SNPs and InDels in the *BADH2* gene were generated using Circos version 0.67 (http://circos.ca/software/download/circos/).

### Haplotype analysis of *BADH2*

Genetic diversity of the *BADH2* gene (chromosome 8, Chr08_20379823-20385975) obtained from the DNA variant VCF file was imported into TASSEL 5.0 [31]. IRGSP-1.0 was used as the reference genome for variant calling. The sequences were aligned using MEGA7 version 7.0 [32]. Haplotype diversity was analyzed using DnaSP version 6 [33]. The aligned DNA sequences were imported into DnaSP version 6 software to calculate π, the number of polymorphic or segregating sites, ThetaW (Watterson estimator, θ_w_), and Tajima’s *D*. Haplotypes were constructed by statistical parsimony using the TCS network with PopART (Population Analysis with Reticulate Trees) version 1.7 [34].

### Variant positioning on the 3D protein structure

A 3D model of dimeric BADH2 (where each monomer consisted of 504 amino acids) was built by homology modeling using MODELLER version 9.21 and by searching for structures related to a novel gene encoding *Trichomonas vaginalis* lactate dehydrogenase (TvLDH) using a Python script.

### RNA-Seq analysis

Total RNA was extracted from 297 rice accessions grown in the paddy field at the Kongju National University, Korea. Panicles were collected at the milky stage (after 15 days of heading) and immediately transferred to liquid nitrogen. Total RNA was extracted from milky stage panicles using the RNeasy® Plant Mini Kit (Qiagen) as per the manufacturer’s instructions. The RNA quality was confirmed by 1.0% agarose gel electrophoresis and quantified using the NanoDrop ND-1000 (NanoDrop Technologies, Wilmington, DE, USA). In addition, the RNA 6000 Pico Kit (Agilent Technologies, Santa Clara, CA, USA) was used to evaluate RNA integrity. Five micrograms of total RNA were used as input material for RNA sample preparation and sequencing libraries were generated using the TruSeq RNA Sample Preparation Kit (Illumina). Finally, the constructed cDNA libraries were sequenced on the Illumina HiSeq 2500 sequencing platform (Illumina). Trimmomatic was used to remove adapters and low-quality reads with default parameters. The clean reads were mapped to IRGSP 1.0 using the R Trinity package. Expression levels quantified in FPKM (fragments per kilobase of transcript per million mapped reads) were standardized using z-scores (zero-mean normalization) [35] and 18 accessions with absolute values > 2 were defined as outliers. Then, the FPKM values of 279 rice accessions were normalized.

### Protein extraction and digestion

Proteins were extracted following the method described by Lee *et al*. [36] with some modifications. In brief, rice milky stage grains were homogenized and suspended in an extraction buffer composed of Tris-HCl (100 mM, pH 8.5), EDTA (1 mM), DTT (5 mM), and dodecyl-β-maltoside (2 % m/v). After 30 min of incubation at room temperature (25°C), samples were centrifuged for 15 min at 14,000*g*. The supernatant was filtered through 5 μm and 0.45 μm membrane filters, sequentially (Millipore, Billerica, MA, USA). Protein extracts were precipitated overnight with trichloroacetic acid (20% v/v), washed with cold acetone three times, and fully dissolved in 8 M urea/Tris-HCl pH 8.5. The protein concentration was determined using the 2D-Protein Quant Kit (GE Healthcare, Piscataway, NJ, USA).

The proteins (500 μg) were reduced with 5 mM Tris (2-carboxyethyl) phosphine hydrochloride (TCEP) at room temperature for 30 min. Then, alkylation was performed with 10 mM iodoacetamide at room temperature in the dark for 30 min. Subsequently, samples were diluted with Tris-HCl (100 mM, pH 8.5) to reduce the urea concentration from 8 M to 2 M and digested with trypsin (5 μg) overnight at 37°C. Protein digestion was terminated with formic acid (5%). Then, digested samples were desalted the using SPEC PLUS PT C18 column (Agilent Technologies). SpeedVac was used to dry the solvent.

### LC-MS/MS

Samples were analyzed by A Nano LC connected to a Finnigan LTQ mass spectrometer (Thermo Scientific, Waltham, MA, USA). Biphasic columns were organized using 365-μm o.d. × 100-µm i.d. fused-silica capillaries (Polymicro Technologies, Phoenix, AZ, USA). The desalted proteins were loaded onto the column. A binary buffer system with 0.1% formic acid (buffer A) and acetonitrile in 0.1% formic acid (buffer B) was used. A linear gradient from 3% buffer B to 50% buffer B at a flow rate of 0.200 µl/min was used for separation. An 11-step program with increasing concentrations of salt solution was used for peptide elution. The run time was 120 min for each step, and approximately 22 h for the 11 steps. Peptide eluent ionization was performed by electrospraying directly into the MS/MS system, and parent-ions were scanned in the range of 400–1600 *m*/*z*. MS/MS-ion scanning of the top five most intense parent ions was performed by collusion-induced dissociation.

### Association studies

The high-quality SNPs (MAF ≥ 5% and missing rate ≤ 20%) obtained by whole-genome resequencing were used in a genome wide analysis of associations with *BADH2* transcript levels and protein abundance. To detect genetic variants associated with *BADH2* transcript levels, 279 accessions were subjected to an eQTL analysis using the mixed linear model implemented in the GAPIT R package [37]. The same package was used for pQTL mapping considering SNPs from 64 accessions as markers, with the *BADH2* protein abundance as the phenotype. The R package qqman (https://cran.r-project.org/web/packages/qqman/index.html) was used to draw Manhattan plots. A SNP with a significant association with *BADH2* transcript expression and protein abundance was defined as an eQTL and pQTL, respectively, a cut-off − log_10_(*p*-value) of < 4. A SNP mapped 1 Mb upstream or downstream of the target gene was considered as a *cis*-QTL, while the remaining SNPs were referred as *trans*-QTLs.

### Analysis of volatile compounds by HS-SPME/GC–MS

The volatile profiles of rice panicles from the milky stage were analyzed according to Lee *et al.* [38], with slight modifications. Briefly, sample powders (500 mg) from 421 accessions (**Table S1**) were placed in a headspace vial and spiked with 2,4,6-trimethylpyridine as an internal standard. The preheated volatiles were adsorbed onto a SPME fiber (divinylbenzene/carboxen/polydimethylsiloxane StableFlex fiber; Supelco, Bellefonte, PA, USA) and injected into a GC/MS instrument (QP2010 Ultra, Shimadzu, Japan) equipped with an Rxi-5Sil MS capillary column (30 m × 0.25 mm ID; Restek, Bellefonte, PA, USA). Volatile compounds were identified based on the n-alkane (C8 to C20)-based retention index for each peak in comparison with the NIST08 (Shimadzu, Japan) and FFNSC 2 (Flavor and Fragrance Natural and Synthetic Compounds, version 2.0, Japan) mass spectral libraries. The peak area of the total ion chromatogram of each compound, including 2AP (*m*/*z* 83), was defined as the relative amount of compound and used for statistical analyses (SPSS, version 24). A partial least squares discriminant analysis (PLS-DA) and variable importance in projection (VIP) were performed using MetaboAnalyst 4.0 (http://www.metaboanalyst.ca/) [39] after log transformation of the data followed by Pareto scaling. We also performed the sensory test of grain aroma by using a 1.7% potassium hydroxide (KOH) solution (**Supplementary methods**).

### Data availability

The whole-genome resequencing data for rice accessions are available in NCBI under the BioProjects PRJNA664261 and PRJNA564458.

## Results

### Genome-wide SNPs identified in 475 accessions

A total of 39,461,811,585 reads were resequenced with a depth of 20.81×, resulting in 24,919,662 SNPs (Table 1). After removing SNPs MAF < 0.05, and missing call rate > 0.2 as well as bi-allele, 3,136,635 SNPs remained. These SNPs consisted of 2,480,451 intergenic and 656,184 genic (319,751 in exon and 336,433 in intron). Further SNPs from exon were grouped in coding sequence (CDS), 3′UTR, and 5′UTR with 190,148, 82,444, and 47,159 SNPs, respectively. The ratio of nonsynonymous to synonymous SNPs was 1.15 with 101,890 nonsynonymous and synonymous 88,258 SNPs (Table 1).

**Table 1:** Genome-wide SNPs in a 475-accession rice core set.

**Chromosome**	**Intergenic**	**Genic**	**Exon**	**Intron**	**CDS**	**3**′**UTR**	**5**′**UTR**	**Synonymous**	**Nonsynonymous**
1	260,131	86,168	43,078	43,090	25,388	11,473	6,217	11,785	13,603
2	226,368	68,127	32,258	35,869	18,608	8,670	4,980	8,725	9,883
3	212,860	56,486	25,219	31,267	14,272	7,015	3,932	6,642	7,630
4	216,651	54,545	27,988	26,557	16,474	7,003	4,511	7,700	8,774
5	177,452	39,765	19,206	20,559	11,301	5,188	2,717	5,353	5,948
6	214,465	54,140	26,536	27,604	15,657	7,042	3,837	7,275	8,382
7	199,199	55,988	26,738	29,250	16,705	6,324	3,709	7,778	8,927
8	192,398	47,535	22,294	25,241	12,623	5,968	3,703	5,911	6,712
9	160,607	37,728	17,541	20,187	10,661	4,406	2,474	4,948	5,713
10	200,020	46,311	23,041	23,270	13,772	5,850	3,419	6,277	7,495
11	228,213	67,004	35,224	31,780	22,287	8,204	4,733	10,009	12,278
12	192,087	42,387	20,628	21,759	12,400	5,301	2,927	5,855	6,545

**Total**	2,480,451	656,184	319,751	336,433	190,148	82,444	47,159	88,258	101,890

MAF ≥ 0.05, Missing Rate < 0.2) CDS: coding sequence; UTR: untranslated region.

### Population genetic structure of a 475-accession core set

**Fig. 1:**
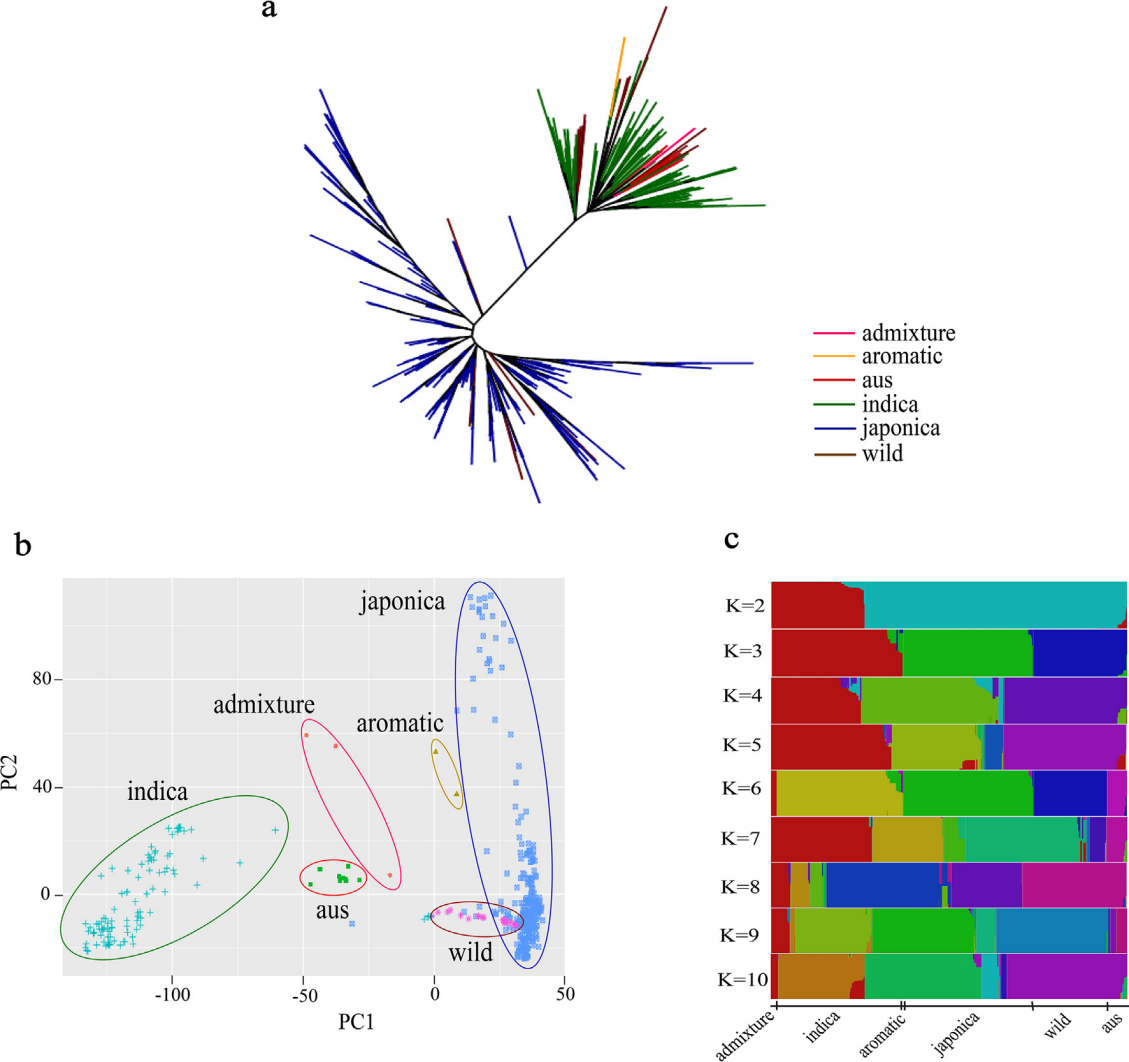
Phylogenetic tree and population structure of 475 rice accessions. **a,** Phylogenetic tree constructed using the neighbor-joining method based on SNPs from whole-genome sequencing. **b,** Principal component analysis (PCA) of cultivated and wild rice accessions. **c,** Population structure clustering with K values from 2 to 10.

A phylogenetic tree was derived from the SNPs called from whole-genome resequencing data for the 475 accessions (Fig. 1). This core set was classified into six groups, including two major groups (indica and japonica), three small varietal groups (aus, admixture, and aromatic), and a small group of wild rice accessions. A PCA (Fig. 1**b**) confirmed the results of the phylogenetic analysis. The indica accessions clustered with aus, aromatic, and admixture (Fig. 1**a**). The japonica group was closely related to the aromatic and wild rice groups in the PCA (Fig. 1**b**). The wild rice group showed overlap with the japonica group in both analysis types. Furthermore, we analyzed the population structure using K values ranging from 2 to 10 and found that a K value of 6 was optimal, with clear separation among groups (Fig. 1**c**).

### Genetic variation in BADH2 of the 475-accession core set

A total of 321 variants, including 209 SNPs, 79 deletions, and 33 insertions, were identified in the seven groups for *BADH2* region (Table 2). The transition/transversion (Ts/Tv) ratio was 1.789. Moreover, the average nonsynonymous/synonymous site diversity (π_non_/ π_syn_) ratio was higher than 1, indicating a high level of nucleotide polymorphism (Table 2).

**Table 2:** Summary of the distribution of nucleotide polymorphisms detected in the coding region of the *BADH2* gene in different groups of rice accessions and results of polymorphism, statistical, and haplotype analyses using japonica as the reference.

**Population**		**admixture**	**aromatic**	**aus**	**indica**	**temperate japonica**	**tropical japonica**	**wild**	**Total/Average**
**Number of accessions**		3	2	9	102	279	26	54	**475**

**Distribution of nucleotide variations**	Deletions (Del)	6	3	4	9	7	7	43	**79**
	Insertions (In)	0	1	2	3	1	1	25	**33**
	SNPs	18	3	20	25	18	15	110	**209**
	Total (SNPs and InDels)	24	7	26	37	26	23	178	**321**
	Heterozygotes	11	0	1	16	4	0	51	**83**
	Transition (Ts)	12	2	13	16	11	11	71	**136**
	Transversion (Tv)	6	1	7	9	7	7	39	**76**
	Ts/Tv	2.000	2.000	1.857	1.778	1.571	1.571	1.821	**1.789**

**Polymorphism, statistical and haplotype analyses of the coding region**	No. of segregating sites (S)	212	568	97	569	1,105	568	22	**1,128**
	Nucleotide diversity (π)	0.0703	0.2518	0.0229	0.0728	0.0400	0.0990	0.0027	**0.0485**
	ThetaW (ϴ_W_)	0.0766	0.2518	0.0227	0.0727	0.1456	0.0980	0.0033	**0.1505**
	Average nonsynonymous site diversity (π_non_)	0.0711	0.2608	0.0224	0.0754	0.0414	0.1025	0.1025	**0.0499**
	Average synonymous site diversity (π_syn_)	0.0677	0.2241	0.0227	0.0649	0.0359	0.0881	0.0881	**0.0444**
	π_non_/π_syn_	1.0499	1.1639	0.9894	1.1614	1.1517	1.16392	1.1639	**1.1239**
	Number of haplotypes	2	2	3	4	5	2	31	**38**
	Haplotype diversity (Hd)	0.5	0.6667	0.711	0.2766	0.1554	0.262	0.9199	**0.3422**
	Theta (q)	0.0766	0.2518	0.0227	0.0727	0.1456	0.0980	0.0033	**0.1505**
	Tajima's D test	−0.8742	n.a.	0.0547	0.0074	−2.2854^**^	0.04076	−0.5893	−2.0686*

Statistical significance is indicated by ^**^P < 0.01 and *P < 0.05; n.a. indicates not determined.

**Fig. 2:**
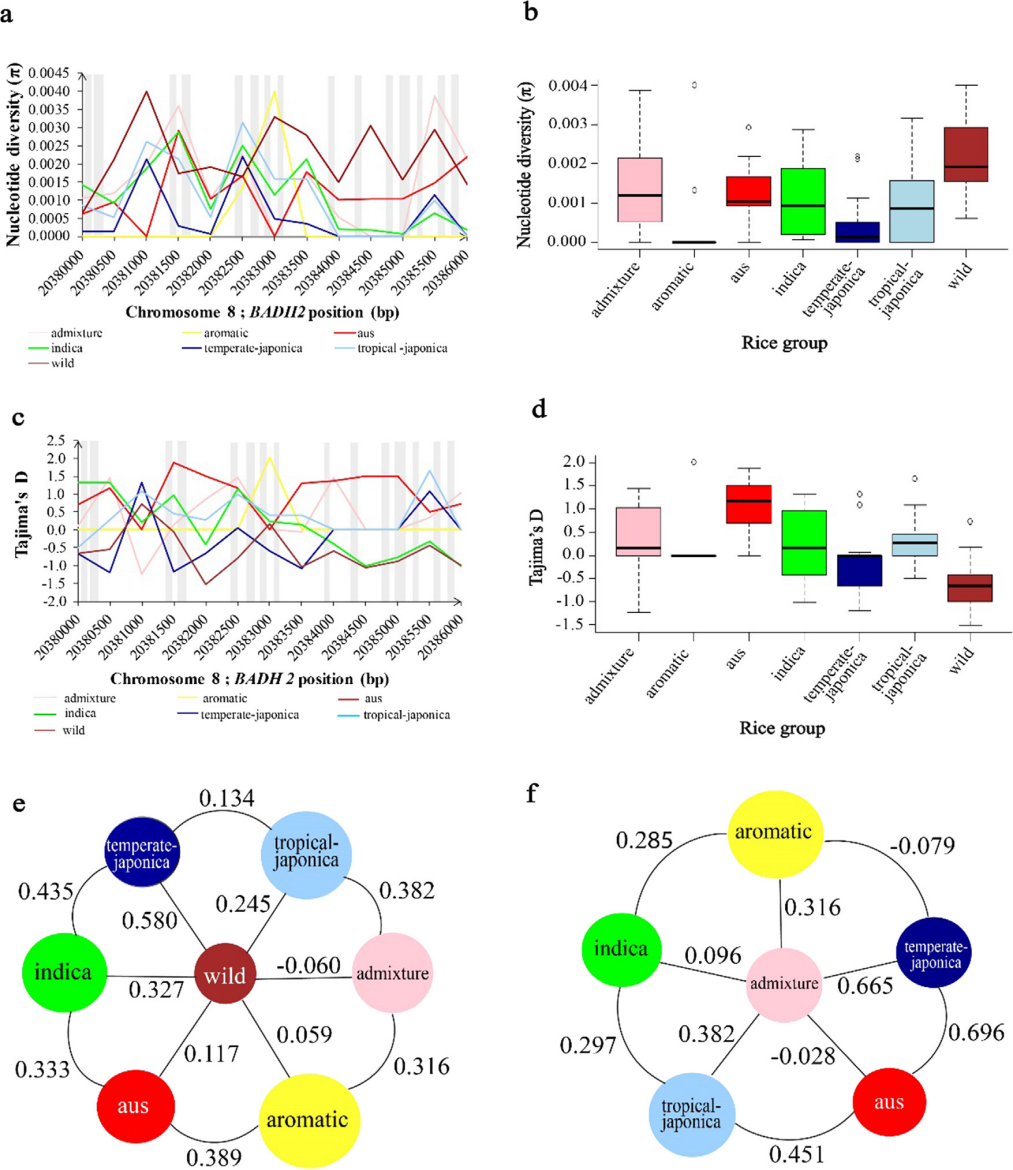
Genetic diversity in the *BADH2* gene in the core set of 475 rice accessions. A, b, Nucleotide diversity (π) in different subgroups with a 500-bp sliding window. The coding region on exons 1 to 15 is shown in the gray-colored columns. C, d, Tajima’s D values for the *BADH2* region. E, f, Fixation index (*F_ST_*) for pairwise comparisons among groups based on 26 and 4 novel alleles, respectively*.*

Nucleotide diversity in the *BADH2* region was highest in the wild group, followed by the admixture, aus, indica, tropical-japonica, and temperate-japonica groups (Fig. 2**a**). The Tajima’s D value was the more negative in the wild group than in temperate-japonica, indica, and tropical-japonica (Fig. 2**d**). The fixation index (*F_ST_*) revealed a very high level of gene flow (low *F_ST_*) between the wild and admixture groups, with *F_ST_* = −0.060, compared with higher estimates between the wild and aromatic groups (0.059), wild and aus groups (0.117), wild and tropical-japonica groups (0.245), wild and indica groups (0.327), and wild and temperate-japonica groups (0.580) (Fig. 2**e**). In addition, an analysis of novel alleles revealed that breeding between the temperate-japonica group and the aromatic group was markedly more frequent than that between the temperate-japonica and aus groups (Fig. 2**f**).

### No signature of selection during domestication

Likelihood ratio tests showed that *BADH2* did not experience significant positive selection between *O. sativa* and *O. rufipogon* or between *O. sativa* or *O. rufipogon* and other plants (**Supplementary methods and** Fig. S2). These results suggest that *BADH2* did not show adaptive evolution after divergence from the most recent common ancestor of *O. sativa* and *O. rufipogon*, implying that variation in *BADH2* was shaped by recent selection, such as domestication and recent selective pressure.

### Haplotypes of *BADH2* in the 475-accession core set

**Fig. 3:**
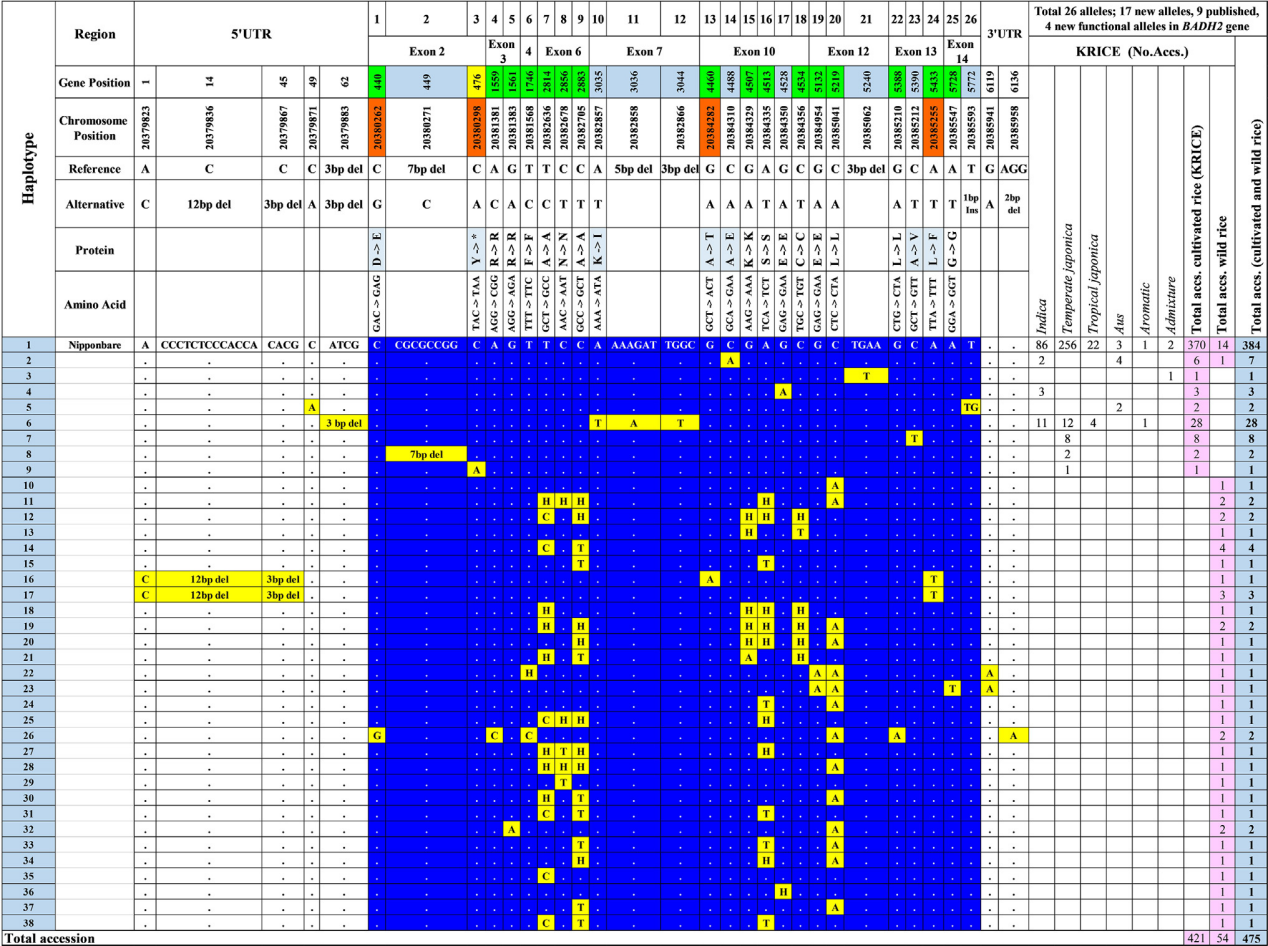
Summary of novel alleles and haplotypes of *BADH2*. The four novel alleles and their chromosomal positions are shown (orange-colored boxes). The published alleles and their positions are shown (blue-colored box).

We found 33 polymorphic sites in the *BADH2* transcript region, including 26 SNPs and InDel alleles in the coding region, five alleles in the 5′UTR, and two alleles in the 3′UTR (Figs. 3**, S3**). Thirty-eight haplotypes were identified based on 26 SNPs within the coding region of *BADH2* (**Tables S2, S3**). The most common haplotype (Hap_1) was found in 384 rice accessions (Fig. S4). Eight haplotypes (Hap_2 to Hap_9) belonged to cultivated rice accessions and seven (Hap_2 to Hap_8) were identified as by previously reported functional alleles (**Tables S1, S2**). One of the newly identified haplotypes, Hap_9, was distinguished by a substitution (C/A) at nucleotide position 476 at exon 2 (*BADH2*-E2-476C > A) in a cultivated accession (RWG-431). Interestingly, all of the 29 other newly identified haplotypes (Hap_10 to Hap_38) were found in wild rice accessions (**Table S3**). Three of the haplotypes were functional: Hap_16 with a SNP (G/A) at nucleotide position 4460 in exon 10 (*BADH2-*E10-4460G > A) found in an *O. glumaepatula* accession (RWG-459), Hap_17 with a SNP (A/T) at nucleotide position 5433 in exon 13 (*BADH2-*E13-5433A > T) detected in two accessions of *O. glumaepatula* (RWG-460 and RWG-461), and Hap_26 with a SNP (C/G) at nucleotide position 440 in exon 2 (*BADH2-*E2-440C > G) found in two accessions of *O. meridionalis* (RWG-475 and RWG-476) (**Tables S2, S3**). Of note, more haplotypes were identified in wild rice accessions than in cultivated rice accessions (**Table S3**). Next, we performed cloning and sequencing of the coding region of *BADH2* from accessions with the four novel SNPs and confirmed the SNPs by a sequence alignment against a reference (Fig. S5).

### Effects of variants on the 3D protein structure of BADH2

**Fig. 4:**
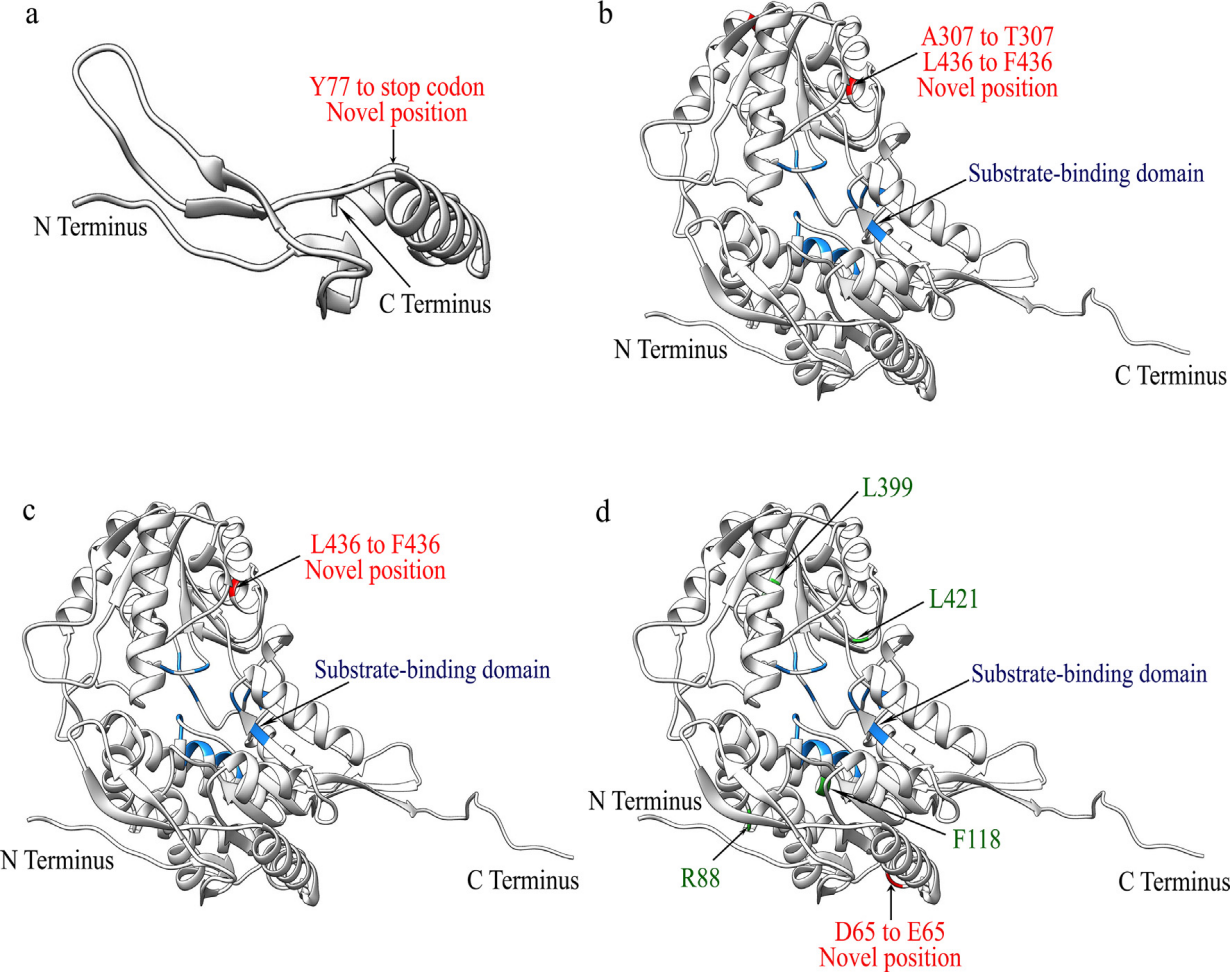
Graphical representation of the three-dimensional protein structure of BADH2. The four novel functional sites are shown in red, the substrate-binding domain is presented in blue, and the published functional sites are shown in green. **a,** Novel functional site in cultivated rice accession RWG-431 at protein position Y77 (Hap_9). **b,** Novel functional sites in wild rice at protein positions A307 and L436 (Hap_16). **c,** Novel functional site in wild rice at protein position L436 (Hap_17). **d,** Novel functional site in wild rice at protein position D65 (Hap_26).

We analyzed the three-dimensional structures of proteins with the four nonsynonymous SNPs and found a substitution in the α-helix active site of accessions, indicating a loss of BADH2 activity (Fig. 4). In Hap_9, the SNP (C/A) resulted in a substitution from tyrosine (TAC) to a stop codon (TAA), resulting in premature termination. In Hap_16, the nonpolar alanine became the polar threonine at 307^th^ amino acid. In Hap_17, leucine became phenylalanine (both nonpolar) at 436^th^ amino acid. In Hap_26, aspartic acid became glutamic acid (both polar) at 65^th^ amino acid (Fig. 4**a–d**).

### Expression quantitative trait loci associated with *BADH2*

**Fig. 5:**
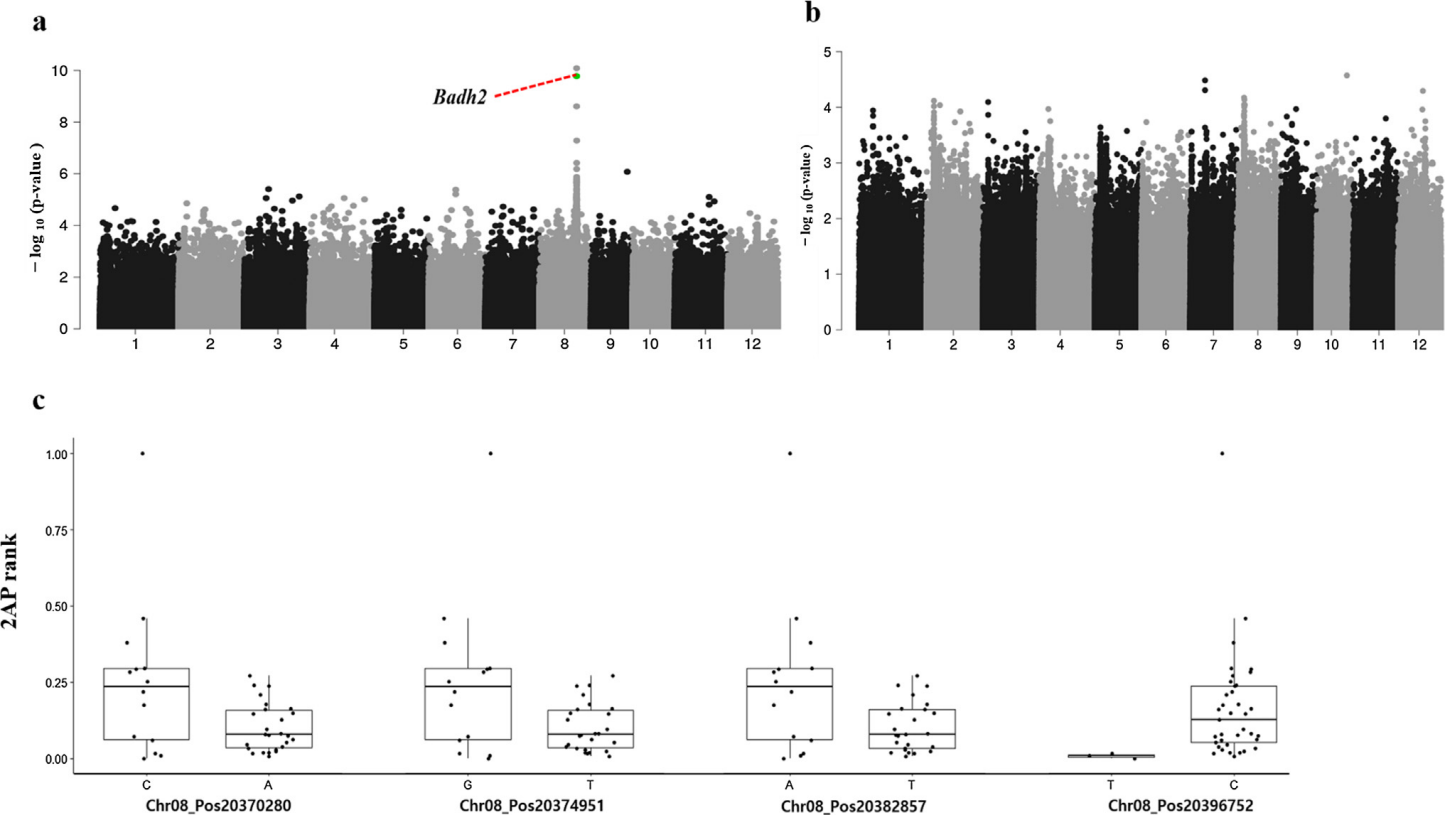
Associatton studies. **a,** Manhattan plot of significant eQTLs for *BADH2* gene expression. **b,** Manhattan plot of significant of pQTL analysis of 64 accessions. **c,** Unpaired two-sample Wilcoxon test for reprentative eQTLs and their effects on phenotype.

Next, we quantified the expression levels of the *BADH2* gene and performed a genome-wise association study of *BADH2* expression levels based on RNA-Seq data from a subset of 279 rice accessions. We found that the expression level of Os08g0424500 (*BADH2*) was regulated by 316 eQTLs with -log_10_*P* > 4 (Fig. 5**a, Table S4**). Among these, 201 eQTLs were located on chromosome 8 and 185 eQTLs were within 1 Mbp of the *BADH2* region at positions 20379823–20385975, suggesting that *cis-*SNPs had a greater contribution than *trans-*SNPS to *BADH2* expression (**Table S4**). The flanking region about 20 kb upstream and about 12 kb downstream of *BADH2* had loci with the most significant effects. A total of 63 eQTLs were located on 22 genes, such as dynamin family protein (Os08g0425100), eight hypothetical conserved proteins, cytokinin oxidase/dehydrogenase 10 (Os06g0572300), and carbonic anhydrase endoglucanase 21 (Os08g0424100). The remaining 253 eQTLs were in intergenic regions (**Table S4**). An eQTL (9_19892312 bp) from chromosome 9 located in intergenic region of two *β*-glucosidase genes (Os09g0511700 and Os09g0511600) had significant effects on *BADH2* expression*.* Glucosidase enzymes hydrolyze glycosides and release aromatic components with a natural flavor [40].

### Protein quantitative trait loci for BADH2

We further quantified the level of BADH2 protein expression and performed a genome-wide association study of BADH2 protein levels. We quantified the BADH2 protein level by LC-MS/MS in 64 accessions selected based on weak to strong fragrance for the pQTL analysis. We identified 13 significant pQTLs (*−*log_10_*P* > 4) on chromosomes 2, 7, 8, 10, and 12 (Fig. 5**b, Table S5)**. All five pQTLs from chromosome 8 were not within 1 Mbp of the *BADH2* region. Chromosome 10:19402186 pQTL had the most significant *P*-value (4.57), while two pQTLs on chromosome 2 were located within Os02g0184800 and Os02g0184900, of which Os02g0184800 is a conserved hypothetical protein and Os02g0184900 is a cytochrome P450, CYP71D8L (**Table S5**).

We assessed evidence for an association between QTLs and 2AP accumulation using the unpaired two-samples Wilcoxon rank sum test on 2AP-producing accessions (accessions with mutant *BADH2* alleles). We considered sample size ≥ 3, removed ‘N’ genotypes or missing values and selected five most significant SNP markers from eQTL and pQTL analysis. The Wilcoxon test showed that the four markers Chr08_20382857, Chr08_20374951, Chr08_20370280 and Chr08_20396752 had the most highly significant effect on the 2AP phenotype with *p* < 0.06 (Fig. 5**c**).

### Volatile profiling and discriminant analysis

We carried out an aroma test with the 421 cultivated accessions from the core set. Based the panel assessment, samples were divided into four categories: nonfragrant, slightly fragrant, moderately fragrant, and strongly fragrant. In total, 50 accessions were categorized as fragrant, of which 25 accessions were slightly fragrant, 22 were moderately fragrant, and 3 were classified as strongly fragrant (**Table S1**). The aceession RWG-431 with a novel allele (*badh2-*E2-476C > A) showed a 2AP peak and was classified as moderately fragrant (**Table S1**). Five accessions of wild rice (RWG-459, RWG-460, RWG-461, RWG-475 and RWG-476) with nonsynonymous substitutions were rated as slightly fragrant (**Table S1**).

**Fig. 6:**
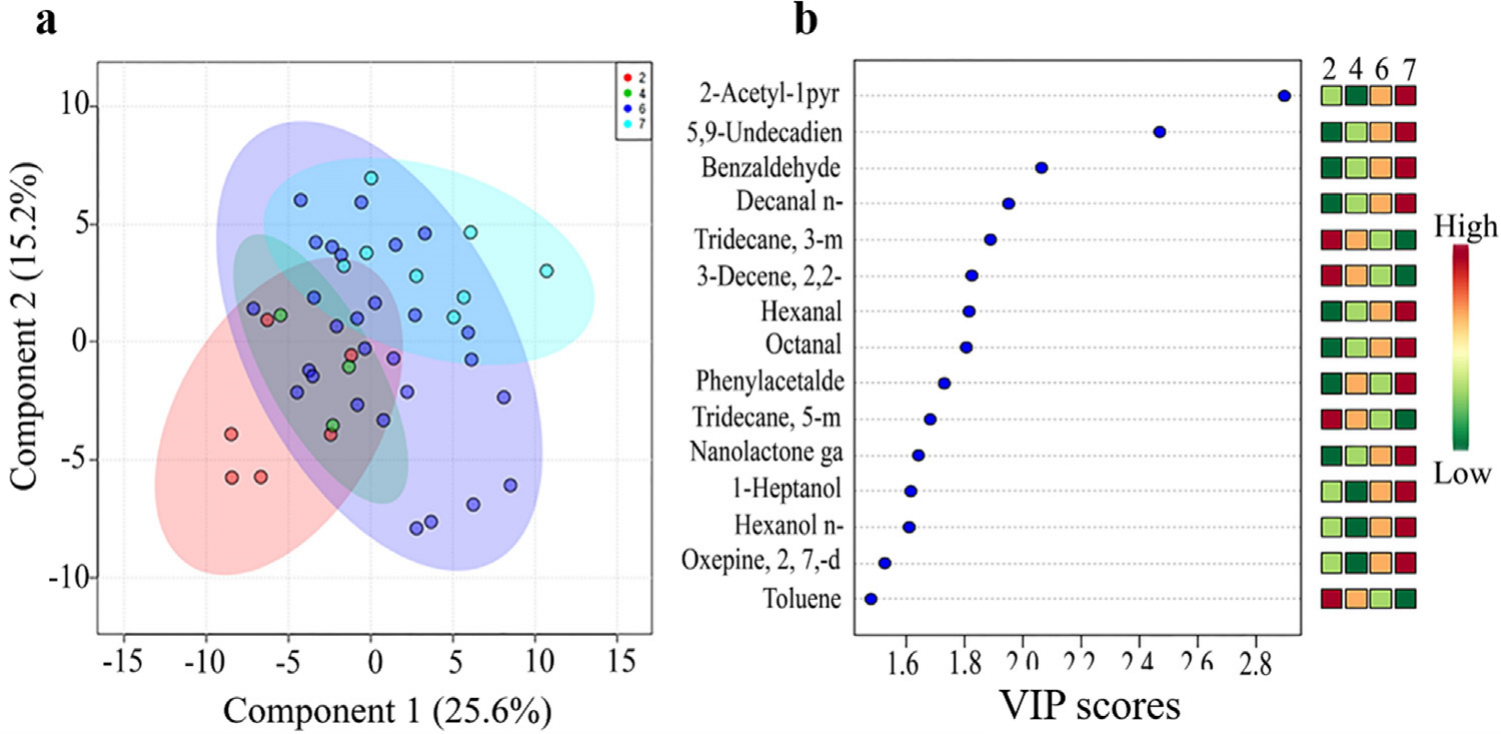
Multivariate analysis of volatiles and *BADH2* haplotypes**. a**, Score plot from a partial least squares discriminant analysis (PLS-DA). **b**, Variable importance in projection (VIP) scores for volatiles discriminating *BADH2* haplotypes. Volatiles detected are 2AP; 5,9-Undecadien-2-one 6,10-dimethyl-, (Z)-; Benzaldehyde, Decanal < n->; Tridecane, 3-methylene-; 3-Decene, 2,2-dimethyl-, (3E); Hexanal; Octanal; Phenylacetaldehyde; Tridecane, 5-methyl-; Gamma-nonalactone; 1-Heptanol; Hexanol < n->; Oxepane-2,7-dione; Toluene.

To examine the volatile profile variation within *BADH2* haplotypes, we performed PLS-DA, estimated VIP scores, and determined the indicator variables that maximize the separation between haplotypes (Fig. 6). Only haplotypes 2, 4, 6, and 7 were used for the PLS-DA, because this analysis requires a sample size of more than three. In the PLS-DA, principal component 1 (PC1) of the score-plot explained 25.6% of the total variance and PC2 explained 15.2% of the variance. Most of the accessions showed overlap on the score-plot, while some accessions from haplotypes 2, 6, and 7 were separated **(**Fig. 6**a**). We then identified the compounds that contribute to the variation observed in PLS-DA by extracting volatiles with a high VIP score (>1.0). The VIP score plots showed that 15 compounds were the main discriminants in the PLS-DA model. Among them, 2AP, 5,9-undecadien-2-one 6,10-dimethyl-(Z), benzaldehyde, n-decanal, hexanal, octanal, phenylacetaldehyde, *gamma*-nonalactone, 1-heptanol, n-hexanol, and oxepane-2,7-dione were significantly more abundant in haplotypes 7 and 6 than in other haplotypes. Haplotypes 7 and 6 were characterized by *badh2-E13-5390C > T* and an 8 bp deletion in exon 7, respectively. In addition to 2AP, benzaldehyde, n-decanal, hexanal, octanal, phenylacetaldehyde, and n-hexanol are known to contribute to rice aroma [38]. In haplotypes 2 and 4, four volatiles (tridecane-3-methylene, 3-decene-2,2-dimethyl-(3E), tridecane-5-methyl-, and toluene) were detected at significantly higher levels than those in other haplotypes **(**Fig. 6**b**); among these four volatiles, toluene is known to confer a sweet and pungent aroma [13]. Therefore, based on the multivariate analysis, 15 volatile compounds could be considered potential biomarkers for establishing the relationship between volatile compounds and fragrance.

## Discussion

High demand for fragrant rice has prompted breeders to identify and characterize *badh2* alleles from different genetic backgrounds and to provide genetic resources for fragrance breeding. In this study, a multi-omics analysis of a Korean collection of 475 rice accessions produced a comprehensive view of this commercially important trait. We performed whole-genome resequencing of 475 rice accessions and identified 26 alleles in the *BADH2* coding region. Among these, eight alleles have been reported previously, including a 7 bp deletion in exon 2 [41], [42], A/T SNP in exon 7 [43], 8 bp deletion in exon 7 [10], [41], [42], C/A SNP in exon 10 [42], G/A SNP in exon 10 [15], [42], 3 bp deletion in exon 12 [44], C/T SNP in exon 13 [42], [45], and 1-bp insertion in exon 14 [42], [45], [46]. Furthermore, we analyzed 3,000 rice genome project (3 K-RGP) data to assess the *BADH2* polymorphisms in a large set of accessions (**Supplementary Results; Tables S6–S8,** Figs. S6–S9). The mean Tajima’s D values for the genic *BADH2* region of 3 K-RGP accessions increased in the following order: aromatic (-3.00), indica (-2.20), admixture (-1.54), aus (-1.171), and japonica (1.41) (**Table S1).** These results indicate that the aromatic, indica, admixture, and aus groups were under positive selection or experienced selective sweeps, while japonica (with a positive value) experienced population bottlenecks or balancing selection. A total of 37 synonymous and nonsynonymous substitutions, including 21 functional SNPs (18 nonsynonymous, one insertion, and two deletions) were found in the *BADH2* coding region of 543 accessions (Fig. S7**, Table S8**)*.* Functional SNPs, *badh2-E7-3035A > T*, and 8 bp deletion in exon 7 characterized for the fragrant phenotype [12], [41], [47], [48] were present in 156 and 110 accessions and represented two major haplotypes (Hap_14 and Hap_16) (**Tables S6, S8)**. Fourteen haplotypes were distinguished by 15 novel functional SNPs (14 nonsynonymous SNPs and one deletion) detected in 77 accessions (**Table S8**). The functional haplotypes identified from the Korean rice collection and 3 K-RGP data will provide important genetic resources for the development of new fragrant rice varieties.

We also tested the effect of the *betaine aldehyde dehydrogenase* homolog *BADH1* (Os04g0464200) on Korean rice fragrance by association analyses (**Supplementary Results; Tables S9–10,** Fig. S10). There were no associations between 2AP accumulation and major *BADH1* haplotypes (Fig. S10). Consistent with our results, He *et al*. [42] also recorded no association between *BADH1* and fragrance in 205 rice accessions, while Singh *et al*. [49] reported a significant association between BADH1 protein haplotypes and aroma score in eighty rice accessions. These contradictory findings may be due to the differences in genotypes, fragrance analysis stage, and classification approaches. A transcript level analysis revealed the constitutive expression of *BADH2* under normal and stress conditions and the upregulation in *BADH1* expression by salt and drought stresses [12], [50], [51]. Various studies have revealed the involvement of *BADH1* in salt stress tolerance [52], [53], [54], [55]. Furthermore, *BADH1* has a much lower affinity for GABAld and higher affinities for other aldehyde substrates than those of *BADH2* [12], [53], [56], [57]. The absence of aroma in *BADH1-*RNAi rice lines [52] suggests physiologically discrete roles of *BADH* homologs. Hence, *BADH2* may substantially diminish the GABAld pool available for 2AP biosynthesis, and *BADH1* cannot compensate for abrogated *BADH2* activity.

Fragrant rice varieties with the same mutant allele or haplotype showed considerable variation in 2AP accumulation, consistent with previous reports [18], [44], [45], [46], [56]. The accessions with Hap_2 carrying *badh2-E10-4488C > A* causing an alanine to glutamic acid substitution [42] and Hap_4 characterized by a synonymous SNP (*badh2-E10-4528G > A*) [15], [44] did not show 2AP accumulation. Such variation in 2AP accumulation in fragrant rice can be attributed to environmental factors and variation in cultivation practices [58], [59], however differences in the levels of transcripts with similar coding sequences also significantly influence plant phenotypes [60], [61].

Despite studies of associations between the *BADH2* region and fragrance, genetic variation at the whole-genome level associated with *BADH2* gene expression levels, contributing to phenotypic variation, are lacking. In plants, most eQTL and pQTL studies are aimed at building a regulatory network to link genetic networks with phenotypic variation [62], [63], [64], [65]. Recently, Anacleto *et al*. [66] reported eQTLs associated with the expression of a candidate gene, *granule-bound starch synthase I* (*GBSSI*), and revealed *cis-*acting functionally relevant genetic variants influencing the glycaemic index and texture in rice. Most eQTLs detected in our study were located in intergenic regions. These regions function via transcription factor binding interactions and regulatory modules and as barriers in nucleosome positioning and organization, determining DNA accessibility [64], [67], [68]. Intergenic regions have been identified in genome-wide association studies of various traits in human and a few plants [62], [69]. Our pQTL analysis revealed the presence of *trans-*pQTLs, as 13 pQTLs were mapped on a different chromosome, 1 Mbp from the location of *BADH2*. The *trans-*pQTLs could be explained by many factors, like post-translational modifications, in addition to gene transcription in the regulation of protein expression [70], [71]. Interestingly, we did not detect any genetic variation in 2AP precursor genes (*GAPDH*, *TPI* and *P5CS*) in eQTL or pQTL analysis, indicating that the correlations between these genes and 2AP accumulation reported earlier [13], [14] are not directly related and rather might be due to the availability and utilization of these gene products leading to 2AP biosynthesis. We detected large number of eQTLs significantly associated with gene expression. However, associations were weaker for pQTLs, possibly because the genetic basis underlying protein expression involves more complex regulation than mRNA abundance [71], [72], [73]. Our study provides evidence supporting the roles of SNPs associated with *BADH2* expression in 2AP variation in fragrant rice.

## Conclusions

We performed whole**-**genome resequencing, transcriptomics, and metabolomics analyses of 475 accessions in the Korean World Rice Collection. The novel functional haplotypes identified from the Korean rice collection and 3 K-RGP data provide important genetic resources for the development of new fragrant rice varieties. High-quality SNPs from whole**-**genome resequencing were used in association analyses with *BADH2* transcript levels and protein abundance. An array of eQTLs and pQTLs associated with *BADH2* expression and protein accumulation are likely regulators mediating 2AP variation in fragrant rice. Indeed, further studies are needed to validate the candidate eQTLs and their interactions. A multivariate analysis identified 15 volatile compounds along with AP as potential biomarkers for rice fragrance. Our study demonstrates the power of integrating genetic, gene expression, and phenotype variation to gain insights into rice fragrance.

## Compliance with Ethics Requirements

*This article does not contain any studies with human or animal subjects.*

## CRediT authorship contribution statement

**Rungnapa Phitaktansakul:** Methodology, Investigation, Validation, Data curation, Writing – original draft. **Kyu-Won Kim:** Methodology, Investigation, Software, Formal analysis, Visualization, Writing- review & editing. **Kyaw Myo Aung:** Investigation, Data curation. **Thant Zin Maung:** Investigation, Data curation. **Myeong-Hyeon Min:** Investigation, Software, Formal analysis. **Aueangporn Somsri:** Investigation, Data curation. **Wondo Lee:** Investigation. Data curation, Resources. **Sang-Beom Lee:** Investigation. Data curation. **Jungrye Nam:** Software, Formal analysis. **Seung-Hyun Kim:** Investigation, Data curation, Methodology. **Joohyun Lee:** Investigation, Data curation, Methodology. **Soon-Wook Kwon:** Investigation, Data curation, Methodology. **Bhagwat Nawade:** Formal analysis, Validation, Writing – review & editing. **Sang-Ho Chu:** Methodology, Project administration, Resources. **Sang-Won Park:** Investigation, Data curation, Methodology. **Kwon Kyoo Kang:** Investigation, Data curation, Methodology. **Yoo-Hyun Cho:** Investigation, Data curation, Resources. **Young-Sang Lee:** Investigation, Methodology, Data curation , Visualization. **Ill-Min Chung:** Conceptualization, Project administration, Fund acquisition , Writing – review & editing. **Yong-Jin Park:** Conceptualization, Project administration, Supervision, Fund acquisition, Supervision, Validation, Resources, Writing – review & editing.

## Declaration of Competing Interest

*The authors declare that they have no known competing financial interests or personal relationships that could have appeared to influence the work reported in this paper.*

## References

[1] Shao G.N., Tang A., Tang S.Q., Luo J., Jiao G.A., Wu J.L. (2011). A new deletion mutation of fragrant gene and the development of three molecular markers for fragrance in rice. Plant Breed.

[2] Giraud G. (2013). The world market of fragrant rice, main issues and perspectives. Int Food Agribus Manag Rev.

[3] Sakthivel K., Sundaram R.M., Shobha Rani N., Balachandran S.M., Neeraja C.N. (2009). Genetic and molecular basis of fragrance in rice. Biotechnol Adv.

[4] Gaur A., Wani S., Deepika P., Bharti N., Malav A., Shikari A. (2016). Understanding the fragrance in rice. Rice Res Open Access.

[5] Nadaf A.B., Wakte K.V., Zanan R.L. (2014). 2-Acetyl-1-pyrroline biosynthesis: from fragrance to a rare metabolic disease. J Plant Sci Res.

[6] Buttery R.G., Ling L.C., Juliano B.O., Turnbaugh J.G. (1983). Cooked rice aroma and 2-acetyl-1-pyrroline. J Agric Food Chem.

[7] Mathure S.V., Jawali N., Thengane R.J., Nadaf A.B. (2014). Comparative quantitative analysis of headspace volatiles and their association with BADH2 marker in non-basmati scented, basmati and non-scented rice (*Oryza sativa* L.) cultivars of India. Food Chem.

[8] Hashemi F.S.G., Rafii M.Y., Ismail M.R., Mahmud T.M.M., Rahim H.A., Asfaliza R. (2013). Biochemical, genetic and molecular advances of fragrance characteristics in rice. Crit Rev Plant Sci.

[9] Ramtekey V., Cherukuri S., Modha K.G., Kumar A., Kethineni U.B., Pal G. (2021). Extraction, characterization, quantification, and application of volatile aromatic compounds from Asian rice cultivars. Rev Anal Chem.

[10] Bradbury L.M.T., Fitzgerald T.L., Henry R.J., Jin Q., Waters D.L.E. (2005). The gene for fragrance in rice. Plant Biotechnol J.

[11] Okpala N.E., Mo Z., Duan M., Tang X. (2019). The genetics and biosynthesis of 2-acetyl-1-pyrroline in fragrant rice. Plant Physiol Biochem.

[12] Chen S., Yang Y.i., Shi W., Ji Q., He F., Zhang Z. (2008). Badh2, encoding betaine aldehyde dehydrogenase, inhibits the biosynthesis of 2-acetyl-1-pyrroline, a major component in rice fragrance. Plant Cell.

[13] Hinge V.R., Patil H.B., Nadaf A.B. (2016). Aroma volatile analyses and 2AP characterization at various developmental stages in Basmati and Non-Basmati scented rice (*Oryza sativa* L.) cultivars. Rice.

[14] Wakte K., Zanan R., Hinge V., Khandagale K., Nadaf A., Henry R. (2017). Thirty-three years of 2-acetyl-1-pyrroline, a principal basmati aroma compound in scented rice (*Oryza sativa* L.): a status review. J Sci Food Agric.

[15] Shao G., Tang S., Chen M., Wei X., He J., Luo J.u. (2013). Haplotype variation at Badh2, the gene determining fragrance in rice. Genomics.

[16] Amarawathi Y., Singh R., Singh A.K., Singh V.P., Mohapatra T., Sharma T.R. (2007). Mapping of quantitative trait loci for basmati quality traits in rice (*Oryza sativa* L.). Mol Breed.

[17] McClung A.M., Edwards J.D., Jia M.H., Huggins T.D., Bockelman H.E., Ali M.L. (2020). Enhancing the searchability, breeding utility, and efficient management of germplasm accessions in the USDA−ARS rice collection. Crop Sci.

[18] Addison C.K., Angira B., Kongchum M., Harrell D.L., Baisakh N., Linscombe S.D. (2020). Characterization of haplotype diversity in the BADH2 aroma gene and development of a KASP SNP assay for predicting aroma in U.S. rice. Rice.

[19] Roy S., Banerjee A., Senapati B.K., Sarkar G. (2012). Comparative analysis of agro-morphology, grain quality and aroma traits of traditional and Basmati-type genotypes of rice *Oryza sativa* L.. Plant Breed.

[20] Fitzgerald M.A., Hamilton N.R.S., Calingacion M.N., Verhoeven H.A., Butardo V.M. (2008). Is there a second fragrance gene in rice?. Plant Biotechnol J.

[21] Scossa F., Alseekh S., Fernie A.R. (2021). Integrating multi-omics data for crop improvement. J Plant Physiol.

[22] Keurentjes J.J.B., Fu J., Terpstra I.R., Garcia J.M., van den Ackerveken G., Snoek L.B. (2007). Regulatory network construction in Arabidopsis by using genome-wide gene expression quantitative trait loci. Proc Natl Acad Sci.

[23] Battle A., Khan Z., Wang S.H., Mitrano A., Ford M.J., Pritchard J.K. (2015). Impact of regulatory variation from RNA to protein. Science.

[24] Kim K.-W., Chung H.-K., Cho G.-T., Ma K.-H., Chandrabalan D., Gwag J.-G. (2007). PowerCore: a program applying the advanced M strategy with a heuristic search for establishing core sets. Bioinformatics.

[25] Kim T.-S., He Q., Kim K.-W., Yoon M.-Y., Ra W.-H., Li F.P. (2016). Genome-wide resequencing of KRICE_CORE reveals their potential for future breeding, as well as functional and evolutionary studies in the post-genomic era. BMC Genomics.

[26] Li H., Durbin R. (2010). Fast and accurate long-read alignment with Burrows-Wheeler transform. Bioinformatics.

[27] McKenna A., Hanna M., Banks E., Sivachenko A., Cibulskis K., Kernytsky A. (2010). The Genome Analysis Toolkit: a MapReduce framework for analyzing next-generation DNA sequencing data. Genome Res.

[28] 3 000 Rice Genomes Project. The 3,000 rice genomes project. GigaScience 2014;3:2047-217X-3–7. 10.1186/2047-217X-3-7 PMC4035669 24872877

[29] Rizzo M.L. (2019).

[30] Raj A., Stephens M., Pritchard J.K. (2014). fastSTRUCTURE: variational inference of population structure in large SNP data sets. Genetics.

[31] Bradbury P.J., Zhang Z., Kroon D.E., Casstevens T.M., Ramdoss Y., Buckler E.S. (2007). TASSEL: software for association mapping of complex traits in diverse samples. Bioinformatics.

[32] Kumar S., Stecher G., Tamura K. (2016). MEGA7: molecular evolutionary genetics analysis version 7.0 for bigger datasets. Mol Biol Evol.

[33] Rozas J., Ferrer-Mata A., Sánchez-DelBarrio J.C., Guirao-Rico S., Librado P., Ramos-Onsins S.E. (2017). DnaSP 6: DNA sequence polymorphism analysis of large data sets. Mol Biol Evol.

[34] Massey A., Seago A. (2017).

[35] Trapnell C., Roberts A., Goff L., Pertea G., Kim D., Kelley D.R. (2012). Differential gene and transcript expression analysis of RNA-seq experiments with TopHat and Cufflinks. Nat Protoc.

[36] Lee J., Koh H.-J. (2011). A label-free quantitative shotgun proteomics analysis of rice grain development. Proteome Sci.

[37] Lipka A.E., Tian F., Wang Q., Peiffer J., Li M., Bradbury P.J. (2012). GAPIT: genome association and prediction integrated tool. Bioinformatics.

[38] Lee Y.-S., Oh Y., Kim T.-H., Cho Y.-H. (2019). Quantitation of 2-acetyl-1-pyrroline in aseptic-packaged cooked fragrant rice by HS-SPME/GC-MS. Food Sci Nutr.

[39] Chong J., Soufan O., Li C., Caraus I., Li S., Bourque G. (2018). MetaboAnalyst 4.0: towards more transparent and integrative metabolomics analysis. Nucleic Acids Res.

[40] Sheng X., Lin Y., Cao J., Ning Y., Pang X., Wu J. (2021). Comparative evaluation of key aroma-active compounds in sweet osmanthus (*Osmanthus fragrans* Lour.) with different enzymatic treatments. J Agric Food Chem.

[41] Shi W., Yang Y.i., Chen S., Xu M. (2008). Discovery of a new fragrance allele and the development of functional markers for the breeding of fragrant rice varieties. Mol Breed.

[42] He Q, Yu J, Kim T-S, Cho Y-H, Lee Y-S, Park Y-J (2015). Resequencing reveals different domestication rate for BADH1 and BADH2 in rice (*Oryza sativa*). PloS One.

[43] Trung K.H., Nguyen T.K., Khuat H.B.T., Nguyen T.D., Khanh T.D., Xuan T.D. (2017). Whole genome sequencing reveals the islands of novel polymorphisms in two native aromatic japonica rice landraces from Vietnam. Genome Biol Evol.

[44] He Q., Park Y.-J. (2015). Discovery of a novel fragrant allele and development of functional markers for fragrance in rice. Mol Breed.

[45] Kovach M.J., Calingacion M.N., Fitzgerald M.A., McCouch S.R. (2009). The origin and evolution of fragrance in rice (*Oryza sativa* L.). Proc Natl Acad Sci.

[46] Dissanayaka S., Kottearachchi N.S., Weerasena J., Peiris M., Virk P. (2014). Development of a CAPS marker for the badh2. 7 allele in SriLankan fragrant rice (*Oryza sativa*). Plant Breed.

[47] Ashokkumar S, Jaganathan D, Ramanathan V, Rahman H, Palaniswamy R, Kambale R, et al. Creation of novel alleles of fragrance gene OsBADH2 in rice through CRISPR/Cas9 mediated gene editing. PloS One 2020;15:e0237018. 10.1371/journal.pone.0237018 PMC7423090 32785241

[48] Li W., Zeng X., Li S., Chen F., Gao J. (2020). Development and application of two novel functional molecular markers of BADH2 in rice. Electron J Biotechnol.

[49] Singh A., Singh P.K., Singh R., Pandit A., Mahato A.K., Gupta D.K. (2010). SNP haplotypes of the BADH1 gene and their association with aroma in rice (*Oryza sativa* L.). Mol Breed.

[50] Niu X., Zheng W., Lu B.-R., Ren G., Huang W., Wang S. (2007). An unusual posttranscriptional processing in two betaine aldehyde dehydrogenase loci of cereal crops directed by short, direct repeats in response to stress conditions. Plant Physiol.

[51] Fitzgerald T.L., Waters D.L.E., Henry R.J. (2008). The effect of salt on betaine aldehyde dehydrogenase transcript levels and 2-acetyl-1-pyrroline concentration in fragrant and non-fragrant rice (*Oryza sativa*). Plant Sci.

[52] Tang W., Sun J., Liu J., Liu F., Yan J., Gou X. (2014). RNAi-directed downregulation of betaine aldehyde dehydrogenase 1 (OsBADH1) results in decreased stress tolerance and increased oxidative markers without affecting glycine betaine biosynthesis in rice (*Oryza sativa*). Plant Mol Biol.

[53] Mitsuya S., Yokota Y., Fujiwara T., Mori N., Takabe T. (2009). OsBADH1 is possibly involved in acetaldehyde oxidation in rice plant peroxisomes. FEBS Lett.

[54] Hasthanasombut S., Paisarnwipatpong N., Triwitayakorn K., Kirdmanee C., Supaibulwatana K. (2011). Expression of OsBADH1 gene in Indica rice (*Oryza sativa* L.) in correlation with salt, plasmolysis, temperature and light stresses. Plant Omics.

[55] Min M.-H., Maung T.Z., Cao Y., Phitaktansakul R., Lee G.-S., Chu S.-H. (2021). Haplotype analysis of BADH1 by next-Generation sequencing reveals association with salt tolerance in rice during domestication. Int J Mol Sci.

[56] Bradbury L.M.T., Gillies S.A., Brushett D.J., Waters D.L.E., Henry R.J. (2008). Inactivation of an aminoaldehyde dehydrogenase is responsible for fragrance in rice. Plant Mol Biol.

[57] Wongpanya R., Boonyalai N., Thammachuchourat N., Horata N., Arikit S., Myint K.M. (2011). Biochemical and enzymatic study of rice BADH wild-type and mutants: an insight into fragrance in rice. Protein J.

[58] Mo Z., Li Y., Nie J., He L., Pan S., Duan M. (2019). Nitrogen application and different water regimes at booting stage improved yield and 2-acetyl-1-pyrroline (2AP) formation in fragrant rice. Rice.

[59] Sansenya S., Wechakorn K. (2021). Effect of rainfall and altitude on the 2-acetyl-1-pyrroline and volatile compounds profile of black glutinous rice (Thai upland rice). J Sci Food Agric.

[60] Ranjan A., Budke J.M., Rowland S.D., Chitwood D.H., Kumar R., Carriedo L. (2016). eQTL regulating transcript levels associated with diverse biological processes in tomato. Plant Physiol.

[61] Kuroha T., Nagai K., Kurokawa Y., Nagamura Y., Kusano M., Yasui H. (2017). eQTLs regulating transcript variations associated with rapid internode elongation in deepwater Rice. Front Plant Sci.

[62] Miculan M., Nelissen H., Ben Hassen M., Marroni F., Inzé D., Pè M.E. (2021). A forward genetics approach integrating genome-wide association study and expression quantitative trait locus mapping to dissect leaf development in maize (*Zea mays*). Plant J.

[63] Tang S., Zhao H.u., Lu S., Yu L., Zhang G., Zhang Y. (2021). Genome- and transcriptome-wide association studies provide insights into the genetic basis of natural variation of seed oil content in *Brassica napus*. Mol Plant.

[64] Pang J., Fu J., Zong N., Wang J., Song D., Zhang X. (2019). Kernel size-related genes revealed by an integrated eQTL analysis during early maize kernel development. Plant J.

[65] Zhou Q., Fu Z., Liu H., Wang J., Guo Z., Zhang X. (2021). Mining novel kernel size-related genes by pQTL mapping and multi-omics integrative analysis in developing maize kernels. Plant Biotechnol J.

[66] Anacleto R., Badoni S., Parween S., Butardo V.M., Misra G., Cuevas R.P. (2019). Integrating a genome-wide association study with a large-scale transcriptome analysis to predict genetic regions influencing the glycaemic index and texture in rice. Plant Biotechnol J.

[67] Wu Y., Zhang W., Jiang J. (2014). Genome-wide nucleosome positioning is orchestrated by genomic regions associated with DNase I hypersensitivity in rice. PLOS Genet.

[68] Zhu B.o., Zhang W., Zhang T., Liu B., Jiang J. (2015). Genome-wide prediction and validation of intergenic enhancers in *Arabidopsis* using open chromatin signatures. Plant Cell.

[69] Ortiz Fernández L., Coit P., Yilmaz V., Yentür S.P., Alibaz‐Oner F., Aksu K. (2021). Genetic association of a gain-of-function IFNGR1 polymorphism and the intergenic region LNCAROD/DKK1 with Behçet’s disease. Arthritis Rheumatol.

[70] Albert FW, Bloom JS, Siegel J, Day L, Kruglyak L. Genetics of trans-regulatory variation in gene expression. ELife 2018;7:e35471. Doi: 10.7554/eLife.35471. 10.7554/eLife.35471 PMC6072440 30014850

[71] Brion C., Lutz S.M., Albert F.W. (2020). Simultaneous quantification of mRNA and protein in single cells reveals post-transcriptional effects of genetic variation. ELife.

[72] He B., Shi J., Wang X., Jiang H., Zhu H.-J. (2020). Genome-wide pQTL analysis of protein expression regulatory networks in the human liver. BMC Biol.

[73] François Y., Vignal A., Molette C., Marty-Gasset N., Davail S., Liaubet L. (2017). Deciphering mechanisms underlying the genetic variation of general production and liver quality traits in the overfed mule duck by pQTL analyses. Genet Sel Evol.

