# Supplementary material for: Multi-omics analysis reveals the genetic basis of rice fragrance mediated by betaine aldehyde dehydrogenase 2
Source: J Adv Res. 2021 Dec 18;42:303–14. doi: 10.1016/j.jare.2021.12.004 (PMC9788947; doi:10.1016/j.jare.2021.12.004)
Supplement: Supplementary data 1 [file mmc1.docx]

**Supplementary information**

**Multi-omics analysis reveals the genetic basis of rice fragrance mediated by *betaine aldehyde dehydrogenase 2***

**Supplementary Methods**

***BADH2* orthologs across plant species**

The protein coding sequence (CDS) the BADH2 from 17 plant species (*Aegilops tauschii*, *Triticum urartu*, *Hordeum vulgare*, *Brachypodium distachyon*, *Leersia perrieri*, *O. barthii*, *O. glaberrima*, *O. longistaminata*, *O. glumaepatula*, *O. nivara*, *O. rufipogon*, *O. sativa*, *O. meridionalis*, *O. punctate*, *O. brachyantha*, *Sorghum bicolor* and *Arabidopsis thaliana*) were downloaded from the Plant Ensembl genome database. The *BADH2* gene was found in 15 plant species, including *O. sativa*, but not in *O. meridionalis* and *O. punctate*, and *BADH2* exhibited one-to-one orthology across the 15 plant species. Orthogroups were identified using Proteinortho version 5.11 with default values. The *BADH2* orthologs were checked for curated gene annotation and the results showed consistency between the curated gene annotations and the set of orthologs.

**Assessment of site-wise adaptive evolution**

The site-wise adaptive evolution of *BADH2* in *O. sativa* and *O. rufipogon* was compared to other plant species using a comparative method for estimation of evolutionary history at a species-wide level.

A branch-site ‘A model’ was used to estimate the ω (ratio of fixation probabilities of nonsynonymous and synonymous substitutions, dN/dS) values. It represents the ratio of nonsynonymous substitutions rate in codon sequences to the synonymous substitutions rate that does not change the amino acid sequence. The nonsynonymous substitutions influenced by selection pressure and potentially change the amino acid sequence resulting non-functional protein are considared. The leaf nodes of *O*. *sativa* and *O*. *rufipogo* were assigned to the foreground branches and the other species to the background branches. The significance of each dN/dS value of the foreground branches were compared with the dN/dS values of the background branches. The branch-site model assumes three classes of ω values: ω_0_<1 for purifying selection, ω_1_=1 for neutral selection, and ω_2_>1 for positive selection. The “A model” of the branch-site model can provide the significance of the degree of difference between the ω values under two hypotheses (H_0_ and H_1_) using the log-likelihood ratio test (LRT). The null hypothesis (H_0_) signifies no evolutionary differentiation between the foreground and background branches, and the alternative hypothesis (H_1_) denotes a significant adaptive evolution at the foreground branches with other branches. Before applying the branch-site model, the *BADH2* orthologs were aligned using PRANK version 100802 [1]. It is one of the most reliable multiple sequence aligners (MSAs), particularly for the alignment of InDels. We used this aligner as any shift in the alignment likely results in the erroneous estimation of codon substitutions and thereby an excessive over-or underestimation of the dN/dS value interpreted as adaptive evolution.

***DNA cloning and sequencing of the novel functional BADH2 alleles***

The four primer sets (**Table S11**) were designed based on the *BADH2* flanking sequence of the reference Nipponbare genome using Primer 3 version 0.4.0 (http://bioinfo.ut.ee/primer3-0.4.0/). The PCR mix (20 µl) consisted 2 µl Thumb buffer (10X), 0.4 µl dNTP mix (0.4 mM), 2 µl 5X Band helper for the high GC content, 0.5 µl each forward and reverse primers (10 µM), 0.2 µl of Thumb Taq polymerase (BioFact, Daejeon, Korea), 2 µl genomic DNA (80 ng/µl), and 10.4 µl nuclease-free water. The PCR was run in a S1000^TM^ Thermal Cycler (Bio-Rad) with the following three-step program: 95°C for 2 min; 35 cycles of 95°C for 2 min, 60°C for 30 seconds, and 72°C for 1 min; and a final extension for 10 min at 72°C. The PCR products were separated on a 3% agarose gel. The amplified products were ligated using the T-Blunt™ PCR Cloning kit (SolGent kit, Korea). The ligation reaction (6-µl) composed of PCR product (4 µl), 6X T-Blunt buffer (1 µl) and T-Bunt vector (1 µl) was kept for 1 h at room temperature. Transformation was done *via* conventional heat shock method into competent *Escherichia coli* (*DH10β*) cells and after colony PCR, plasmids were extracted using a plasmid mini-prep kit (Qiagen). Further, The sequencing was performed for three clones from each accessions and aligned with reference sequence using Clustal Omega ([https://www.ebi.ac.uk/Tools/msa/clustalo/](https://www.ebi.ac.uk/Tools/msa/clustalo/).)).

**Sensory test for fragrance classification**

The fragrance of milk-stage grains from 421 accessions were tested by a sensory analysis according to the method of Sood and Siddiq [2]. Two grams of sample was sliced and mixed with 10 ml of 1.7% KOH solution, then incubated for 10 min in water bath at 30°C, and smelled by five individuals to evaluate aroma. The samples were scored on 1-4 scale with 1, 2, 3 and 4 corresponding to absence of fragrance, slight fragrance, moderate fragrance, and strong fragrance, respectively [3].

**Supplementary Results**

**Genetic variations in the *BADH2* gene of 3K accessions.**

In our core set of 475 accessions, we identified 26 alleles in *BADH2* coding region (**Fig. 3**). Besides, we also analyzed genetic variations in the *BADH2* gene (chromosome 8, Chr08_20379823-20385975) from 3K-RGP accessions (**Tables S6-S8,** **Figs. S6-S9**). In total, 1,065 variants, comprising 1,001 SNPs, 45 deletions, and 19 insertions, were identified in the five rice groups. The highest number of polymorphisms were found in the indica (370, 34.75%) followed by japonica (350, 32.86%), admixture (124, 11.64%), aus (134, 12.58%), and aromatic (87, 8.17%) (**Table S6**). The mean transition/transversion (Ts/Tv) ratio was recorded as 5.326. The number of transitions were higher than the transversions in all groups except indica group. The average haplotype diversity (Hd) was 0.4065 (**Table S6**). Nucleotide diversity (π) value was highest in the japonica group (π =0.39388), followed by the aus, admixture, indica, and aromatic groups. The mean nucleotide diversity (π) was 0.02948. Estimation of the nucleotide diversity based on the Watterson estimator (ThetaW, ϴ_W_) value was also highest in the japonica group (**Table S6)**. All groups except japonica found to have negative Tajima’s D value and most highly negative in aromatic (-3.00), followed by indica (-2.20), admixture (-1.54) and aus (-1.171) groups (**Table S6**). The population differentiation (*F_ST_*) revealed a very high level of breeding (low *Fst* value) between aromatic and admixture with *F_ST_*= 0.043 compared with higher estimates between the japonica and aromatic (0.048), japonica and admixture (0.120), aromatic and indica (0.178), japonica and indica (0.180), indica and aus (0.242), aus and admixture (0.284), aus and aromatic (0.287), japonica and aus (0.425), and aus and admixture (0.451) (**Figure S8)**.

**Novel fragrance alleles in *BADH2* from 3K accessions**.

A total of 150 haplotypes were generated based on the 830 segregating sites of *BADH2* (**Table S6)** and 58 of these haplotypes consisted of 37 SNPs and InDels in the coding *BADH2.* Among 37*,* 21 functional SNPs (18 nonsynonymous, one insertion, and two deletions) were detected coding regions of 543 accessions (**Table S8)**. Fifteen functional SNPs (14 nonsynonymous SNPs and one deletion) were novel and six have been described previously, including a A/T SNP at P_3035_ (P indicate a position in gene region) in exon 7 [4], 8 bp deletion at P_3037_ in exon 7 [5–7], C/A SNP at P_4488_ in exon 10 [7], G/A SNP at P_4528_ in exon 10 [7,8], C/T SNP at P_5390_ in exon 13[7,9], and 1 bp deletion in exon 14 [7,9,10] (**Table** **S8**). Fourteen of the 150 haplotypes were distinguished by newly identified SNPs as following (1) Hap_42: a SNP (P_199_) in exon 1 corresponds to alanine to aspartic acid substitution detected in one accession (IRIS_313-10711), (2) Hap_135: two nonsynonymous SNPs in exon 1 detected in a accession (IRIS_313-9233), one at (P_198_) resulted in alanine to proline substitution and another at (P_208_) caused leucine to histidine substitution, (3) Hap_30: a SNP (P_1771_) in exon 4 (serine to alanine substitution) of one accession (IRIS_313-10057); (4) Hap_35: a SNP (P_1789_) in exon 4 (asparagine to histidine substitution) was found in two accessions (IRIS_313-10440 and IRIS_313-11788), (5) Hap_131: a SNP (P_1810_) in exon 4 (methionine to leucine substitution) detected in a accession (IRIS_313-8927); (6) Hap_76: a SNP (P_2604_) in exon 5 (valine by alanine substitution) was found in one accession (IRIS_313-11557), (7) Hap_37: a SNP (P_2658_) in exon 5 (valine to alanine substitution) of 30 accessions, (8) Hap_14: a SNP (P_3047_) in exon 7 (alanine to valine substitution) of 17 accessions, (9) Hap_81, a 1-bp deletion (P_4036_) in exon 9 was found in one accession (IRIS_313-11678), (10) Hap_52: a SNP (P_4460_) in exon 10 (alanine to threonine substitution) detected in two accessions; (11) Hap_44: a SNP (P_4550_) in exon 10 (valine to isoleucine substitution) was found in one accession (IRIS_313-10722); (12) Hap_79: a SNP (P_5149_) in exon 12 (glutamic acid by alanine substitution) was found in one accession (IRIS_313-11599), (13) Hap_45: a SNP (P_5768_) in exon 14 (proline to serine substitution) in ten accessions, and (14) Hap_46: a SNP (P_5799_) in exon 14 (glycine to valine substitution) was found in seven accessions (**Tables S7–S8***).* Most of the *BADH2* mutant accessions found have to polymorphism in exon 7 with highest 194 accessions for *badh2-E10-4528G>A* allele followed by 156 accessions for *badh2-E7-3035A>T* alleles. Multiple SNPs were recorded in a large number of accessions (**Table S8**). Among groups, indica group showed highest SNPs with 320 accessions, while among 215 aus accessions, 103 accessions were detected with variant allele. In the aromatic group, 42.6% accessions (29) were found with *BADH2* alleles and interestingly, all these accessions had SNPs in exon 7 and exon 10 (**Table** **S8**). The Hap_37 characterized by badh2-E5-2658C>T predominantly contained indica accessions (28 out of 30 accessions (**Tables S6, S8)**. These findings provide a catalog of rice accessions as a resource for fragrance rice breeding programs.

***BADH1* and fragrance association**

We investigated the association between *BADH1*(chr04: 23171516..23176332) and fragrance *via* haplotype analysis. Rice *BADH* homologs, *BADH1* and *BADH2*, share 75% sequence identity are encoded on chromosome four and chromosome eight, respectively [11].

The haplotypes based on SNPs (a minor allele frequency maf filter of <0.03) of the *BADH1* gene [12] were generated using TASSEL 5.0. IRGSP-1.0 was used as the reference genome for variant calling and and a haplotype list was generated by DnaSP version 6.0. In total, 193 variants including, 116 SNPs and 77 InDels were dectected in the *BADH1* gene region. A total of 39 haplotypes were identified in 421 cultivated accessions [12] (**Table S9**). While 2AP producing accessions were grouped in eight haplotypes. There was no speecfic haplotype corresponded with fragrant accessions. Furthermore, no marker was detected with significant *p*-value in association analysis between the phenotype (fragrance) and the *BADH1* region in a general linear model (GLM) in the TASSEL 5 (**Table S10**). Further, to check haplotypes of 2AP-producing accessions, we considered haplotypes with sample size ≥3 for haplotype-trait association analysis (**Table S9**). Wilcoxon rank sum test was used to rank the 2AP-producing accessions and performed assosication analtsis for two major haplotypes (Hap_3 and Hap_18) (**Fig. S10**). The Differences between haplotypes were statistically analyzed using Wilcoxon test and Student's t-test. There were no significant differences between the two haplotypes (**Fig. S10**). Similarly, in resequencing analysis of 205 accessions generated 7 *BADH1* haplotypes and the GLM based association revealed no association between *BADH1* and aroma [7]. The abundance of *BADH1* transcripts was similar in fragrant and non-fragrant rice varieties, while *BADH2* transcripts in non-fragrant varieties were significantly more abundant than *badh2* transcripts in fragrant varieties [13]. Hence *BADH1* is little likely related to rice fragrance.

**Table S1**. Passport information of 475 rice accessions core set used in this study. (Excel file)

**Table S2.** Summary of coding mutations in *BADH2* from 475 rice accessions.

| **Serial number** | **Allele name** | **Novel/ reported** | **Accession no.** | **Varieties** | **Total** |
| --- | --- | --- | --- | --- | --- |
| *badh2*-N001 | *badh2*-E2-440C>G | * | RWG-475, RWG-476 | *Oryza meridionalis* | 2 |
| *badh2*-N002 | *badh2*-E2-450_456delGCGCCGG | *badh2-E2*[6], *badh2.2* [7,9], *badh2.2*[8], *badh2-E2.1*[14] | RWG-413, RWG-424 | Wuxiang 99-8, SD-25 | 2 |
| *badh2*-N003 | *badh2*-E2-476C>A | * | RWG-431 | SP-698 | 1 |
| *badh2*-N004 | *badh2*-E3-1559A>C | * | RWG-475, RWG-476 | *Oryza meridionalis, Oryza meridionalis* | 2 |
| *badh2*-N005 | *badh2*-E3-1561G>A | * | RWG-486, RWG-487 | Oryza glaberrima, Oryza barthii | 2 |
| *badh2*-N006 | *badh2*-E4-1746T>C | * | RWG-475, RWG-476 | *Oryza meridionalis, Oryza meridionalis* | 2 |
| *badh2*-N007 | *badh2*-E6-2814T>C | * | RWG-452, RWG-457, RWG-474, RWG-480, RWG-481, RWG-483, RWG-484 | *Oryza alta, Oryza eichingeri, Oryza malampuzhaensis, Oryza punctata, Oryza rhizomatis, Oryza rhizomatis, Oryza rhizomatis* | 9 |
| *badh2*-N008 | *badh2*-E6-2856C>T | * | RWG-435, RWG-477, RWG-479 | *Oryza eichingeri, Oryza minuta, Oryza punctata* | 3 |
| *badh2*-N009 | *badh2*-E6-2883C>T | * | RWG-434, RWG-457, RWG-458, RWG-467, RWG-480, RWG-481, RWG-482, RWG-483, RWG-484, RWG-444, RWG-447 | *Oryza officinalis, Oryza eichingeri, Oryza eichingeri, Oryza latifolia, Oryza punctata, Oryza punctata, Oryza rhizomatis, Oryza rhizomatis, Oryza rhizomatis, Oryza rufipogon, Oryza glaberrima* | 12 |
| *badh2*-N010 | *badh2*-E7-3035A>T | Shi et al.[6], Trung et al.[4] | RWG-042, RWG-061, RWG-064, RWG-069, RWG-179, RWG-201, RWG-236, RWG-272, RWG-296, RWG-373, RWG-374, RWG-384, RWG-411, RWG-412, RWG-415, RWG-416, RWG-417, RWG-418, RWG-419, RWG-420, RWG-421, RWG-422, RWG-423, RWG-425, RWG-426, RWG-427, RWG-492, RWG-493, RWG-494 | Mushkan, Binato, Hawm Supan, Keng Chi Ju, Heugjinju, Heugkwang, Seonhyangheukmi, Hyangmi 1, Daw Dam, Chungnam 1, Chungnam 2, Heughyangchal1ho, BP603, Daohuaxiang 2, Delmont, IR841-85-1-1-2, Jasmine 85, Zixiangnuo 861, A-1, Goolarath, Hwangmibyeo 2, Seonhyangheukmi, SD-11, E2, BP066, BP444, Hom mali nin surin, Khao Dawk Mali 105, Pathumthani 1 | 29 |
| *badh2*-N011 | *badh2*-E7-3037_3041delAAGAT | *badh2-E7*[6,14]*,* *badh2.7*[8] (Shao et al., 2013), *badh2.1*[7] | RWG-042, RWG-061, RWG-064, RWG-069, RWG-179, RWG-201, RWG-236, RWG-272, RWG-296, RWG-373, RWG-374, RWG-384, RWG-411, RWG-412, RWG-415, RWG-416, RWG-417, RWG-418, RWG-419, RWG-420, RWG-421, RWG-422, RWG-423, RWG-425, RWG-426, RWG-427, RWG-492, RWG-493, RWG-494 | Mushkan, Binato, Hawm Supan, Keng Chi Ju, Heugjinju, Heugkwang, Seonhyangheukmi, Hyangmi 1, Daw Dam, Chungnam 1, Chungnam 2, Heughyangchal1ho, BP603, Daohuaxiang 2, Delmont, IR841-85-1-1-2, Jasmine 85, Zixiangnuo 861, A-1, Goolarath, Hwangmibyeo 2, Seonhyangheukmi, SD-11, E2, BP066, BP444, Hom mali nin surin, Khao Dawk Mali 105, Pathumthani 1 | 29 |
| *badh2*-N012 | *badh2*-E7-3045_3047delGGC | *badh2-E7*[6,14]*,* *badh2.7*[8] (Shao et al., 2013), *badh2.1*[7] | RWG-042, RWG-061, RWG-064, RWG-069, RWG-179, RWG-201, RWG-236, RWG-272, RWG-296, RWG-373, RWG-374, RWG-384, RWG-411, RWG-412, RWG-415, RWG-416, RWG-417, RWG-418, RWG-419, RWG-420, RWG-421, RWG-422, RWG-423, RWG-425, RWG-426, RWG-427, RWG-492, RWG-493, RWG-494 | Mushkan, Binato, Hawm Supan, Keng Chi Ju, Heugjinju, Heugkwang, Seonhyangheukmi, Hyangmi 1, Daw Dam, Chungnam 1, Chungnam 2, Heughyangchal1ho, BP603, Daohuaxiang 2, Delmont, IR841-85-1-1-2, Jasmine 85, Zixiangnuo 861, A-1, Goolarath, Hwangmibyeo 2, Seonhyangheukmi, SD-11, E2, BP066, BP444, Hom mali nin surin, Khao Dawk Mali 105, Pathumthani 1 | 29 |
| *badh2*-N013 | *badh2*-E10-4460G>A | * | RWG-459 | *Oryza glumaepatula* | 1 |
| *badh2*-N014 | *badh2*-E10-4488C>A | *badh2.16*[7] | RWG-008, RWG-037, RWG-043, RWG-044, RWG-113, RWG-137, RWG-438 | Tchampa, Dharial, Pukhi, Red Rice, Spin Mere, IR40, *Oryza nivara* | 7 |
| *badh2*-N015 | *badh2*-E10-4507G>A | * | RWG-466, RWG-467 | *Oryza latifolia, Oryza latifolia* | 2 |
| *badh2*-N016 | *badh2*-E10-4513A>T | * | RWG-458, RWG-473, RWG-481 | *Oryza eichingeri, Oryza malampuzhaensis, Oryza punctata* | 3 |
| *badh2*-N017 | *badh2*-E10-4528G>A | *badh2.17*[7] | RWG-036, RWG-491 | Chiem Chank, Tubtim chumpare | 2 |
| *badh2*-N018 | *badh2*-E10-4534C>T | * | RWG-453 | *Oryza alta* | 1 |
| *badh2*-N019 | *badh2*-E12-5132G>A | * | RWG-470, RWG-471 | *Oryza longistaminata, Oryza longistaminata* |  |
| *badh2*-N020 | *badh2*-E12-5219C>A | * | RWG-459, RWG-460, RWG-462 | *Oryza glumaepatula* | 3 |
| *badh2*-N021 | *badh2*-E12-5241-5243delGAA | *badh2*-E12[14], *badh2.18*[7] | RWG-031 | Mongdonjaerae | 1 |
| *badh2*-N022 | *badh2*-E13-5388G>A | * | RWG-475, RWG-476 | *Oryza meridionalis, Oryza meridionalis* |  |
| *badh2*-N023 | *badh2*-E13-5390C>T | *badh2*-E13.2[14], *badh2.10*[7,9]*,* *badh2.13*[8] | RWG-088, RWG-191, RWG-197, RWG-380, RWG-414, RWG-428, RWG-429, RWG-430 | Mihyang, Seolhyangchal, Aranghangchal, Hyangnam, OITA KAORI INE 2, SP-519, SP-687, SP-688 | 8 |
| *badh2*-N024 | *badh2*-E13-5433A>T | * | RWG-459, RWG-460, RWG-461 | *Oryza glumaepatula* | 3 |
| *badh2*-N025 | *badh2*-E13-5728A>T | * | RWG-470, RWG-471 | *2* | 2 |
| *badh2*-N026 | *badh2*-E14-5772_5773insG | *badh2.7*[7,9]*,* *badh2*-E14.1[14] | RWG-038, RWG-295 | Dular, Tal | 2 |

* : Discovered in this study; In allele name-*badh2* : *betaine aldehyde* dehydrogenase 2, E: exon number, then number indicate gene position

Yellow box: nonsynonymous alleles were validated by DNA cloning and sequencing.

**Table S3.** Haplotype distribution based on *BADH2* coding region in 475 rice accessions.

| **Haplotype** | **Accession no.** | **Total** |
| --- | --- | --- |
| Hap_1 | RWG-001 RWG-002 RWG-003 RWG-004 RWG-005 RWG-006 RWG-007 RWG-009 RWG-010 RWG-011 RWG-012 RWG-013 RWG-014 RWG-015 RWG-016 RWG-017 RWG-018 RWG-019 RWG-020 RWG-021 RWG-022 RWG-023 RWG-024 RWG-025 RWG-026 RWG-027 RWG-028 RWG-029 RWG-030 RWG-032 RWG-033 RWG-034 RWG-035 RWG-039 RWG-040 RWG-041 RWG-045 RWG-046 RWG-047 RWG-048 RWG-049 RWG-050 RWG-051 RWG-052 RWG-053 RWG-054 RWG-055 RWG-056 RWG-057 RWG-058 RWG-059 RWG-060 RWG-062 RWG-063 RWG-065 RWG-066 RWG-067 RWG-068 RWG-071 RWG-072 RWG-073 RWG-074 RWG-075 RWG-076 RWG-077 RWG-078 RWG-079 RWG-080 RWG-081 RWG-082 RWG-083 RWG-084 RWG-085 RWG-086 RWG-087 RWG-089 RWG-090 RWG-091 RWG-092 RWG-093 RWG-094 RWG-095 RWG-096 RWG-097 RWG-098 RWG-099 RWG-100 RWG-101 RWG-102 RWG-103 RWG-104 RWG-105 RWG-106 RWG-107 RWG-108 RWG-109 RWG-110 RWG-111 RWG-112 RWG-114 RWG-115 RWG-116 RWG-117 RWG-118 RWG-119 RWG-120 RWG-121 RWG-122 RWG-123 RWG-124 RWG-125 RWG-126 RWG-127 RWG-128 RWG-129 RWG-130 RWG-131 RWG-132 RWG-133 RWG-134 RWG-135 RWG-136 RWG-138 RWG-139 RWG-140 RWG-141 RWG-142 RWG-143 RWG-144 RWG-145 RWG-146 RWG-147 RWG-148 RWG-149 RWG-150 RWG-151 RWG-152 RWG-153 RWG-154 RWG-155 RWG-156 RWG-157 RWG-158 RWG-159 RWG-160 RWG-161 RWG-162 RWG-163 RWG-164 RWG-165 RWG-166 RWG-167 RWG-168 RWG-169 RWG-170 RWG-171 RWG-172 RWG-173 RWG-174 RWG-175 RWG-176 RWG-177 RWG-178 RWG-180 RWG-181 RWG-182 RWG-183 RWG-184 RWG-185 RWG-186 RWG-187 RWG-188 RWG-189 RWG-190 RWG-192 RWG-193 RWG-194 RWG-195 RWG-196 RWG-198 RWG-199 RWG-200 RWG-202 RWG-203 RWG-204 RWG-205 RWG-206 RWG-207 RWG-208 RWG-209 RWG-210 RWG-211 RWG-212 RWG-213 RWG-214 RWG-215 RWG-216 RWG-217 RWG-218 RWG-219 RWG-220 RWG-221 RWG-222 RWG-223 RWG-224 RWG-225 RWG-226 RWG-227 RWG-228 RWG-229 RWG-230 RWG-231 RWG-232 RWG-233 RWG-234 RWG-235 RWG-237 RWG-238 RWG-239 RWG-240 RWG-241 RWG-242 RWG-243 RWG-244 RWG-245 RWG-246 RWG-247 RWG-248 RWG-249 RWG-250 RWG-251 RWG-252 RWG-253 RWG-254 RWG-255 RWG-256 RWG-257 RWG-258 RWG-259 RWG-260 RWG-261 RWG-262 RWG-263 RWG-264 RWG-265 RWG-266 RWG-267 RWG-268 RWG-269 RWG-270 RWG-271 RWG-273 RWG-274 RWG-275 RWG-276 RWG-277 RWG-278 RWG-279 RWG-280 RWG-281 RWG-282 RWG-283 RWG-284 RWG-285 RWG-286 RWG-287 RWG-288 RWG-289 RWG-290 RWG-291 RWG-292 RWG-293 RWG-294 RWG-297 RWG-298 RWG-299 RWG-316 RWG-317 RWG-318 RWG-319 RWG-320 RWG-321 RWG-322 RWG-323 RWG-324 RWG-325 RWG-326 RWG-327 RWG-328 RWG-329 RWG-330 RWG-331 RWG-332 RWG-333 RWG-334 RWG-335 RWG-336 RWG-337 RWG-338 RWG-339 RWG-340 RWG-341 RWG-342 RWG-343 RWG-344 RWG-345 RWG-346 RWG-347 RWG-348 RWG-349 RWG-350 RWG-351 RWG-352 RWG-353 RWG-354 RWG-355 RWG-356 RWG-357 RWG-358 RWG-359 RWG-360 RWG-361 RWG-362 RWG-363 RWG-364 RWG-365 RWG-366 RWG-367 RWG-368 RWG-369 RWG-370 RWG-371 RWG-372 RWG-375 RWG-376 RWG-377 RWG-378 RWG-379 RWG-381 RWG-382 RWG-383 RWG-385 RWG-386 RWG-387 RWG-388 RWG-389 RWG-390 RWG-391 RWG-392 RWG-393 RWG-394 RWG-395 RWG-396 RWG-397 RWG-398 RWG-399 RWG-400 RWG-401 RWG-402 RWG-403 RWG-404 RWG-405 RWG-406 RWG-407 RWG-408 RWG-409 RWG-410 RWG-432 RWG-433 RWG-436 RWG-437 RWG-439 RWG-440 RWG-444 RWG-445 RWG-447 RWG-450 RWG-451 RWG-454 RWG-455 RWG-456 RWG-468 RWG-469 RWG-485 | 384 |
| Hap_2 | RWG-008 RWG-037 RWG-043 RWG-044 RWG-113 RWG-137 RWG-438 | 7 |
| Hap_3 | RWG-031 | 1 |
| Hap_4 | RWG-036 RWG-070 RWG-491 | 3 |
| Hap_5 | RWG-038 RWG-295 | 2 |
| Hap_6 | RWG-042 RWG-061 RWG-064 RWG-069 RWG-179 RWG-201 RWG-236 RWG-272 RWG-296 RWG-373 RWG-374 RWG-384 RWG-411 RWG-412 RWG-415 RWG-416 RWG-417 RWG-418 RWG-419 RWG-420 RWG-421 RWG-422 RWG-423 RWG-425 RWG-426 RWG-427 RWG-492 RWG-493 RWG-494 | 29 |
| Hap_7 | RWG-088 RWG-191 RWG-197 RWG-380 RWG-414 RWG-428 RWG-429 RWG-430 | 8 |
| Hap_8 | RWG-413 RWG-424 | 2 |
| Hap_9 | RWG-431 | 1 |
| Hap_10 | RWG-448 | 1 |
| Hap_11 | RWG-449 RWG-472 | 2 |
| Hap_12 | RWG-452 RWG-462 | 2 |
| Hap_13 | RWG-453 | 1 |
| Hap_14 | RWG-457 RWG-482 RWG-483 RWG-484 | 4 |
| Hap_15 | RWG-458 | 1 |
| Hap_16 | RWG-459 | 1 |
| Hap_17 | RWG-460 RWG-461 | 2 |
| Hap_18 | RWG-463 | 1 |
| Hap_19 | RWG-464 RWG-466 | 2 |
| Hap_20 | RWG-465 | 1 |
| Hap_21 | RWG-467 | 1 |
| Hap_22 | RWG-470 | 1 |
| Hap_23 | RWG-471 | 1 |
| Hap_24 | RWG-473 | 1 |
| Hap_25 | RWG-474 | 1 |
| Hap_26 | RWG-475 RWG-476 | 2 |
| Hap_27 | RWG-477 | 1 |
| Hap_28 | RWG-478 | 1 |
| Hap_29 | RWG-479 | 1 |
| Hap_30 | RWG-480 | 1 |
| Hap_31 | RWG-481 | 1 |
| Hap_32 | RWG-486 RWG-487 | 2 |
| Hap_33 | RWG-434 | 1 |
| Hap_34 | RWG-443 | 1 |
| Hap_35 | RWG-435 | 1 |
| Hap_36 | RWG-441 | 1 |
| Hap_37 | RWG-442 | 1 |
| Hap_38 | RWG-446 | 1 |

Yellow box -Novel functional haplotypes

**Table S4.** Summary of significant eQTLs detected for BADH2 expression. (Excel file)

**Table S5.** Summary of significant pQTLs. (Excel file)

**Table S6.**  Summary of the nucleotide variations detected in 3K rice accessions using the *BADH2* gene region.

| **Population** | | **Admixture** | **Aromatic** | **Aus** | **Japonica** | **Indica** | **Total** |
| --- | --- | --- | --- | --- | --- | --- | --- |
| **Number of accessions** | | 135 | 68 | 215 | 839 | 1,743 | **3,000** |
| Number of distributions of nucleotide variations | Deletions (Del) | 10 | 7 | 8 | 8 | 12 | **45** |
|  | Insertions (Ins) | 1 | 3 | 6 | 4 | 5 | **19** |
|  | SNPs | 113 | 77 | 120 | 228 | 353 | **1,001** |
|  | Total (SNPs and InDels) | 124 | 87 | 134 | 350 | 370 | **1,065** |
|  | Heterozygotes | 118 | 23 | 93 | 270 | 288 | **792** |
|  | Transitions (Ts) | 61 | 40 | 64 | 174 | 164 | **503** |
|  | Transversion (Tv) | 52 | 37 | 56 | 164 | 189 | **498** |
|  | Ts/Tv | 1.17308 | 1.08108 | 1.14286 | 1.06098 | 0.86772 | **5.32572** |
| Polymorphism, statistical test, and haplotype analysis in coding region | Number of segregating sites (S) | 574 | 350 | 596 | 1,179 | 774 | **830** |
|  | Nucleotide diversity (π) | 0.03739 | 0.01493 | 0.15546 | 0.39388 | 0.02319 | **0.02948** |
|  | Theta W (𝛉w) | 0.06981 | 0.10314 | 0.24503 | 0.26727 | 0.10105 | **0.09079** |
|  | Average nonsynonymous site diversity (non) | 0.0386 | 0.01495 | 0.14265 | 0.39325 | 0.02375 | **0.03015** |
|  | π_non_/π_syn_ | 1.14608 | 1.03892 | 0.89245 | 0.99436 | 1.12346 | **1.12879** |
|  | Number of haplotypes | 12 | 6 | 13 | 105 | 84 | **150** |
|  | Haplotype diversity (Hd) | 0.3541 | 0.1407 | 0.6317 | 0.9337 | 0.3977 | **0.4065** |
|  | Tajima’s D Test | -1.53588 | -3.00137 *** | -1.17087 | 1.40698 | -2.20935 | **-1.89633** |

**Table S7.** Haplotype distribution based on *BADH2* gene in 3K rice accessions.

| **Haplotype** | **Accession no.** | **Total** |
| --- | --- | --- |
| Hap_1 | Reference B001 B002 B003 B004 B005 B006 B007 B008 B009 B010 B011 B012 B013 B015 B016 B017 B018 B019 B020 B021 B024 B026 B027 B028 B029 B030 B031 B032 B033 B035 B036 B037 B038 B039 B040 B043 B044 B046 B047 B048 B051 B052 B053 B054 B055 B056 B057 B058 B059 B060 B061 B062 B063 B064 B066 B067 B068 B069 B070 B071 B072 B073 B074 B075 B079 B081 B084 B085 B086 B087 B088 B089 B090 B091 B092 B093 B094 B095 B097 B100 B103 B104 B105 B106 B107 B108 B109 B112 B114 B115 B116 B117 B118 B119 B121 B122 B124 B125 B126 B127 B128 B130 B131 B132 B134 B135 B136 B138 B139 B140 B142 B143 B144 B145 B146 B147 B148 B149 B151 B152 B153 B155 B156 B157 B158 B159 B160 B161 B162 B163 B165 B166 B167 B168 B169 B170 B171 B173 B176 B179 B180 B181 B182 B183 B187 B188 B189 B190 B191 B192 B193 B194 B195 B196 B197 B198 B199 B200 B201 B202 B204 B205 B207 B208 B210 B212 B213 B214 B215 B216 B217 B218 B219 B222 B223 B224 B225 B226 B228 B229 B230 B232 B233 B234 B235 B236 B238 B239 B240 B241 B242 B244 B245 B246 B247 B248 B249 B250 B258 B259 B260 B261 B263 B264 B265 B266 B267 B268 B269 CX10 CX101 CX102 CX103 CX106 CX107 CX109 CX11 CX110 CX111 CX113 CX114 CX115 CX116 CX117 CX118 CX119 CX12 CX121 CX123 CX125 CX126 CX128 CX129 CX13 CX130 CX131 CX132 CX133 CX134 CX138 CX139 CX14 CX140 CX141 CX142 CX143 CX144 CX145 CX146 CX147 CX149 CX15 CX150 CX151 CX152 CX153 CX154 CX155 CX156 CX158 CX16 CX160 CX161 CX162 CX165 CX17 CX18 CX19 CX2 CX20 CX205 CX206 CX207 CX21 CX210 CX211 CX213 CX214 CX218 CX219 CX22 CX220 CX221 CX225 CX226 CX227 CX228 CX230 CX231 CX232 CX233 CX234 CX235 CX236 CX237 CX238 CX24 CX240 CX241 CX242 CX243 CX247 CX248 CX249 CX25 CX250 CX251 CX26 CX262 CX263 CX265 CX267 CX268 CX269 CX27 CX270 CX273 CX274 CX275 CX276 CX277 CX278 CX28 CX280 CX282 CX284 CX285 CX286 CX287 CX288 CX29 CX290 CX291 CX296 CX3 CX30 CX303 CX305 CX306 CX307 CX31 CX313 CX314 CX316 CX317 CX318 CX319 CX32 CX328 CX329 CX33 CX330 CX34 CX340 CX342 CX343 CX344 CX345 CX346 CX347 CX348 CX349 CX35 CX350 CX351 CX352 CX353 CX354 CX356 CX357 CX358 CX359 CX361 CX364 CX366 CX369 CX37 CX370 CX372 CX373 CX374 CX375 CX376 CX377 CX378 CX379 CX380 CX381 CX382 CX383 CX384 CX385 CX386 CX387 CX388 CX389 CX390 CX391 CX393 CX394 CX395 CX396 CX397 CX4 CX403 CX42 CX431 CX44 CX45 CX47 CX48 CX49 CX5 CX50 CX51 CX52 CX534 CX54 CX542 CX548 CX55 CX56 CX57 CX58 CX6 CX60 CX63 CX68 CX70 CX71 CX73 CX74 CX75 CX76 CX77 CX78 CX79 CX8 CX80 CX82 CX83 CX84 CX85 CX86 CX87 CX88 CX89 CX9 CX90 CX91 CX92 CX93 CX94 CX96 CX97 CX98 CX99 IRIS_313-10000 IRIS_313-10001 IRIS_313-10002 IRIS_313-10007  IRIS_313-10010 IRIS_313-10014 IRIS_313-10020 IRIS_313-10026 IRIS_313-10030 IRIS_313-10034 IRIS_313-10035 IRIS_313-10040 IRIS_313-10041 IRIS_313-10045 IRIS_313-10046 IRIS_313-10047 IRIS_313-10048 IRIS_313-10051 IRIS_313-10054 IRIS_313-10056 IRIS_313-10059 IRIS_313-10061 IRIS_313-10062 IRIS_313-10065 IRIS_313-10067 IRIS_313-10071 IRIS_313-10073 IRIS_313-10074 IRIS_313-10076 IRIS_313-10077 IRIS_313-10078 IRIS_313-10079 IRIS_313-10080 IRIS_313-10082 IRIS_313-10083 IRIS_313-10084 IRIS_313-10089 IRIS_313-10092 IRIS_313-10093 IRIS_313-10094 IRIS_313-10096 IRIS_313-10097 IRIS_313-10099 IRIS_313-10102 IRIS_313-10103 IRIS_313-10109 IRIS_313-10111 IRIS_313-10113 IRIS_313-10114 IRIS_313-10119 IRIS_313-10124 IRIS_313-10129 IRIS_313-10148 IRIS_313-10152 IRIS_313-10154 IRIS_313-10158 IRIS_313-10161 IRIS_313-10164 IRIS_313-10167 IRIS_313-10168 IRIS_313-10170 IRIS_313-10171 IRIS_313-10176 IRIS_313-10178 IRIS_313-10179 IRIS_313-10189 IRIS_313-10190 IRIS_313-10191 IRIS_313-10196 IRIS_313-10211 IRIS_313-10220 IRIS_313-10221 IRIS_313-10224 IRIS_313-10226 IRIS_313-10228 IRIS_313-10234 IRIS_313-10235 IRIS_313-10237 IRIS_313-10238 IRIS_313-10239 IRIS_313-10242 IRIS_313-10247 IRIS_313-10257 IRIS_313-10258 IRIS_313-10260 IRIS_313-10263 IRIS_313-10268 IRIS_313-10271 IRIS_313-10272 IRIS_313-10274 IRIS_313-10275 IRIS_313-10285 IRIS_313-10289 IRIS_313-10290 IRIS_313-10293 IRIS_313-10295 IRIS_313-10298 IRIS_313-10300 IRIS_313-10301 IRIS_313-10307 IRIS_313-10314 IRIS_313-10318 IRIS_313-10325 IRIS_313-10327 IRIS_313-10333 IRIS_313-10334 IRIS_313-10336 IRIS_313-10340 IRIS_313-10341 IRIS_313-10348 IRIS_313-10349 IRIS_313-10352 IRIS_313-10353 IRIS_313-10355 IRIS_313-10357 IRIS_313-10359 IRIS_313-10360 IRIS_313-10361 IRIS_313-10366 IRIS_313-10371 IRIS_313-10373 IRIS_313-10374 IRIS_313-10375 IRIS_313-10379 IRIS_313-10380 IRIS_313-10385 IRIS_313-10392 IRIS_313-10394 IRIS_313-10396 IRIS_313-10398 IRIS_313-10399 IRIS_313-10400 IRIS_313-10401 IRIS_313-10402 IRIS_313-10404 IRIS_313-10412 IRIS_313-10417 IRIS_313-10421 IRIS_313-10422 IRIS_313-10428 IRIS_313-10429 IRIS_313-10430 IRIS_313-10433 IRIS_313-10437 IRIS_313-10444 IRIS_313-10448 IRIS_313-10449 IRIS_313-10450 IRIS_313-10452 IRIS_313-10453 IRIS_313-10458 IRIS_313-10459 IRIS_313-10469 IRIS_313-10476 IRIS_313-10477 IRIS_313-10484 IRIS_313-10485 IRIS_313-10489 IRIS_313-10497 IRIS_313-10502 IRIS_313-10503 IRIS_313-10504 IRIS_313-10506 IRIS_313-10507 IRIS_313-10509 IRIS_313-10511 IRIS_313-10514 IRIS_313-10515 IRIS_313-10516 IRIS_313-10517 IRIS_313-10518 IRIS_313-10519 IRIS_313-10520 IRIS_313-10522 IRIS_313-10524 IRIS_313-10526 IRIS_313-10527 IRIS_313-10534 IRIS_313-10536 IRIS_313-10537 IRIS_313-10539 IRIS_313-10541 IRIS_313-10542 IRIS_313-10543 IRIS_313-10545 IRIS_313-10547 IRIS_313-10550 IRIS_313-10552 IRIS_313-10554 IRIS_313-10556 IRIS_313-10558 IRIS_313-10559 IRIS_313-10560 IRIS_313-10561 IRIS_313-10562 IRIS_313-10563 IRIS_313-10564 IRIS_313-10565 IRIS_313-10566 IRIS_313-10567 IRIS_313-10568 IRIS_313-10570 IRIS_313-10573 IRIS_313-10575 IRIS_313-10576 IRIS_313-10577 IRIS_313-10580 IRIS_313-10581 IRIS_313-10582 IRIS_313-10583 IRIS_313-10585 IRIS_313-10587 IRIS_313-10592 IRIS_313-10594 IRIS_313-10600 IRIS_313-10603 IRIS_313-10604 IRIS_313-10605 IRIS_313-10606 IRIS_313-10608 IRIS_313-10609 IRIS_313-10614 IRIS_313-10617 IRIS_313-10618 IRIS_313-10619 IRIS_313-10620 IRIS_313-10628 IRIS_313-10631 IRIS_313-10640 IRIS_313-10642 IRIS_313-10644 IRIS_313-10645 IRIS_313-10648 IRIS_313-10650 IRIS_313-10651 IRIS_313-10652 IRIS_313-10653 IRIS_313-10654 IRIS_313-10655 IRIS_313-10656 IRIS_313-10657 IRIS_313-10660 IRIS_313-10661 IRIS_313-10664 IRIS_313-10667 IRIS_313-10668 IRIS_313-10670 IRIS_313-10671 IRIS_313-10673 IRIS_313-10674 IRIS_313-10675 IRIS_313-10676 IRIS_313-10677 IRIS_313-10678 IRIS_313-10679 IRIS_313-10680 IRIS_313-10684 IRIS_313-10687 IRIS_313-10688 IRIS_313-10689 IRIS_313-10690 IRIS_313-10693 IRIS_313-10694 IRIS_313-10695 IRIS_313-10698 IRIS_313-10699 IRIS_313-10701 IRIS_313-10702 IRIS_313-10703 IRIS_313-10704 IRIS_313-10706 IRIS_313-10707 IRIS_313-10708 IRIS_313-10710 IRIS_313-10712 IRIS_313-10716 IRIS_313-10717 IRIS_313-10718 IRIS_313-10721 IRIS_313-10723 IRIS_313-10724 IRIS_313-10726 IRIS_313-10727 IRIS_313-10728 IRIS_313-10730 IRIS_313-10731 IRIS_313-10738 IRIS_313-10739 IRIS_313-10740 IRIS_313-10741 IRIS_313-10743 IRIS_313-10744 IRIS_313-10745 IRIS_313-10746 IRIS_313-10747 IRIS_313-10748 IRIS_313-10749 IRIS_313-10750 IRIS_313-10752 IRIS_313-10753 IRIS_313-10754 IRIS_313-10755 IRIS_313-10756 IRIS_313-10757 IRIS_313-10759 IRIS_313-10761 IRIS_313-10765 IRIS_313-10766 IRIS_313-10767 IRIS_313-10769 IRIS_313-10770 IRIS_313-10771 IRIS_313-10772 IRIS_313-10773 IRIS_313-10774 IRIS_313-10775 IRIS_313-10776 IRIS_313-10777 IRIS_313-10778 IRIS_313-10779 IRIS_313-10781 IRIS_313-10783 IRIS_313-10784 IRIS_313-10785 IRIS_313-10786 IRIS_313-10787 IRIS_313-10789 IRIS_313-10790 IRIS_313-10791 IRIS_313-10793 IRIS_313-10794 IRIS_313-10795 IRIS_313-10796 IRIS_313-10797 IRIS_313-10798 IRIS_313-10799 IRIS_313-10801 IRIS_313-10802 IRIS_313-10804 IRIS_313-10805 IRIS_313-10806 IRIS_313-10807 IRIS_313-10808 IRIS_313-10809 IRIS_313-10810 IRIS_313-10811 IRIS_313-10813 IRIS_313-10814 IRIS_313-10815 IRIS_313-10816 IRIS_313-10817 IRIS_313-10818 IRIS_313-10819 IRIS_313-10823 IRIS_313-10824 IRIS_313-10827 IRIS_313-10828 IRIS_313-10829 IRIS_313-10830 IRIS_313-10831 IRIS_313-10832 IRIS_313-10834 IRIS_313-10835 IRIS_313-10836 IRIS_313-10837 IRIS_313-10838 IRIS_313-10839 IRIS_313-10840 IRIS_313-10841 IRIS_313-10842 IRIS_313-10844 IRIS_313-10845 IRIS_313-10847 IRIS_313-10848 IRIS_313-10851 IRIS_313-10853 IRIS_313-10857 IRIS_313-10858 IRIS_313-10862 IRIS_313-10863 IRIS_313-10864 IRIS_313-10865 IRIS_313-10866 IRIS_313-10867 IRIS_313-10870 IRIS_313-10873 IRIS_313-10879 IRIS_313-10880 IRIS_313-10881 IRIS_313-10882 IRIS_313-10883 IRIS_313-10884 IRIS_313-10885 IRIS_313-10886 IRIS_313-10888 IRIS_313-10889 IRIS_313-10890 IRIS_313-10892 IRIS_313-10893 IRIS_313-10894 IRIS_313-10895 IRIS_313-10896 IRIS_313-10902 IRIS_313-10903 IRIS_313-10904 IRIS_313-10905 IRIS_313-10906 IRIS_313-10908 IRIS_313-10909 IRIS_313-10912 IRIS_313-10915 IRIS_313-10916 IRIS_313-10917 IRIS_313-10923 IRIS_313-10924 IRIS_313-10926 IRIS_313-10928 IRIS_313-10929 IRIS_313-10931 IRIS_313-10932 IRIS_313-10933 IRIS_313-10936 IRIS_313-10937 IRIS_313-10940 IRIS_313-10941 IRIS_313-10942 IRIS_313-10943 IRIS_313-10944 IRIS_313-10945 IRIS_313-10946 IRIS_313-10947 IRIS_313-10948 IRIS_313-10949 IRIS_313-10950 IRIS_313-10951 IRIS_313-10952 IRIS_313-10953 IRIS_313-10954 IRIS_313-10956 IRIS_313-10957 IRIS_313-10958 IRIS_313-10959 IRIS_313-10963 IRIS_313-10967 IRIS_313-10968 IRIS_313-10971 IRIS_313-10972 IRIS_313-10973 IRIS_313-10974 IRIS_313-10975 IRIS_313-10977 IRIS_313-10978 IRIS_313-10980 IRIS_313-10982 IRIS_313-10983 IRIS_313-10984 IRIS_313-10986 IRIS_313-10987 IRIS_313-10988 IRIS_313-10989 IRIS_313-10991 IRIS_313-10995 IRIS_313-10996 IRIS_313-10997 IRIS_313-10998 IRIS_313-10999 IRIS_313-11000 IRIS_313-11001 IRIS_313-11003 IRIS_313-11004 IRIS_313-11005 IRIS_313-11006 IRIS_313-11007 IRIS_313-11008 IRIS_313-11009 IRIS_313-11013 IRIS_313-11015 IRIS_313-11020 IRIS_313-11022 IRIS_313-11024 IRIS_313-11025 IRIS_313-11028 IRIS_313-11031 IRIS_313-11032 IRIS_313-11033 IRIS_313-11034 IRIS_313-11035 IRIS_313-11036 IRIS_313-11037 IRIS_313-11038 IRIS_313-11039 IRIS_313-11040 IRIS_313-11041 IRIS_313-11044 IRIS_313-11046 IRIS_313-11048 IRIS_313-11049 IRIS_313-11051 IRIS_313-11053 IRIS_313-11054 IRIS_313-11057 IRIS_313-11058 IRIS_313-11059 IRIS_313-11060 IRIS_313-11073 IRIS_313-11074 IRIS_313-11075 IRIS_313-11077 IRIS_313-11078 IRIS_313-11081 IRIS_313-11082 IRIS_313-11084 IRIS_313-11085 IRIS_313-11086 IRIS_313-11087 IRIS_313-11089 IRIS_313-11091 IRIS_313-11092 IRIS_313-11093 IRIS_313-11094 IRIS_313-11096 IRIS_313-11098 IRIS_313-11099 IRIS_313-11100 IRIS_313-11101 IRIS_313-11102 IRIS_313-11103 IRIS_313-11104 IRIS_313-11105 IRIS_313-11106 IRIS_313-11107 IRIS_313-11108 IRIS_313-11109 IRIS_313-11111 IRIS_313-11113 IRIS_313-11114 IRIS_313-11115 IRIS_313-11118 IRIS_313-11119 IRIS_313-11120 IRIS_313-11121 IRIS_313-11122 IRIS_313-11123 IRIS_313-11124 IRIS_313-11125 IRIS_313-11127 IRIS_313-11128 IRIS_313-11129 IRIS_313-11130 IRIS_313-11131 IRIS_313-11134 IRIS_313-11136 IRIS_313-11137 IRIS_313-11138 IRIS_313-11139 IRIS_313-11140 IRIS_313-11141 IRIS_313-11142 IRIS_313-11145 IRIS_313-11146 IRIS_313-11147 IRIS_313-11148 IRIS_313-11149 IRIS_313-11150 IRIS_313-11152 IRIS_313-11153 IRIS_313-11155 IRIS_313-11156 IRIS_313-11157 IRIS_313-11158 IRIS_313-11160 IRIS_313-11161 IRIS_313-11162 IRIS_313-11163 IRIS_313-11165 IRIS_313-11167 IRIS_313-11169 IRIS_313-11170 IRIS_313-11171 IRIS_313-11177 IRIS_313-11183 IRIS_313-11189 IRIS_313-11191 IRIS_313-11192 IRIS_313-11193 IRIS_313-11194 IRIS_313-11195 IRIS_313-11197 IRIS_313-11198 IRIS_313-11199 IRIS_313-11200 IRIS_313-11201 IRIS_313-11202 IRIS_313-11203 IRIS_313-11204 IRIS_313-11205 IRIS_313-11206 IRIS_313-11207 IRIS_313-11208 IRIS_313-11209 IRIS_313-11211 IRIS_313-11212 IRIS_313-11214 IRIS_313-11217 IRIS_313-11219 IRIS_313-11220 IRIS_313-11221 IRIS_313-11223 IRIS_313-11224 IRIS_313-11225 IRIS_313-11226 IRIS_313-11227 IRIS_313-11228 IRIS_313-11229 IRIS_313-11230 IRIS_313-11231 IRIS_313-11233 IRIS_313-11234 IRIS_313-11236 IRIS_313-11237 IRIS_313-11238 IRIS_313-11239 IRIS_313-11240 IRIS_313-11241 IRIS_313-11242 IRIS_313-11244 IRIS_313-11245 IRIS_313-11246 IRIS_313-11247 IRIS_313-11248 IRIS_313-11249 IRIS_313-11250 IRIS_313-11251 IRIS_313-11253 IRIS_313-11254 IRIS_313-11255 IRIS_313-11256 IRIS_313-11257 IRIS_313-11258 IRIS_313-11259 IRIS_313-11260 IRIS_313-11261 IRIS_313-11262 IRIS_313-11263 IRIS_313-11264 IRIS_313-11266 IRIS_313-11267 IRIS_313-11268 IRIS_313-11270 IRIS_313-11271 IRIS_313-11272 IRIS_313-11273 IRIS_313-11275 IRIS_313-11277 IRIS_313-11279 IRIS_313-11280 IRIS_313-11281 IRIS_313-11282 IRIS_313-11285 IRIS_313-11288 IRIS_313-11289 IRIS_313-11290 IRIS_313-11292 IRIS_313-11293 IRIS_313-11294 IRIS_313-11295 IRIS_313-11297 IRIS_313-11299 IRIS_313-11301 IRIS_313-11302 IRIS_313-11303 IRIS_313-11305 IRIS_313-11307 IRIS_313-11308 IRIS_313-11309 IRIS_313-11310 IRIS_313-11311 IRIS_313-11312 IRIS_313-11313  IRIS_313-11314 IRIS_313-11316 IRIS_313-11317 IRIS_313-11319 IRIS_313-11320 IRIS_313-11321 IRIS_313-11322 IRIS_313-11325 IRIS_313-11326 IRIS_313-11328 IRIS_313-11334 IRIS_313-11335 IRIS_313-11336 IRIS_313-11337 IRIS_313-11339 IRIS_313-11342 IRIS_313-11344 IRIS_313-11345 IRIS_313-11346 IRIS_313-11349 IRIS_313-11351 IRIS_313-11354 IRIS_313-11355 IRIS_313-11357 IRIS_313-11358 IRIS_313-11359 IRIS_313-11360 IRIS_313-11361 IRIS_313-11363 IRIS_313-11364 IRIS_313-11365 IRIS_313-11367 IRIS_313-11368 IRIS_313-11369 IRIS_313-11370 IRIS_313-11372 IRIS_313-11374 IRIS_313-11375 IRIS_313-11376 IRIS_313-11379 IRIS_313-11380 IRIS_313-11382 IRIS_313-11385 IRIS_313-11386 IRIS_313-11389 IRIS_313-11390 IRIS_313-11391 IRIS_313-11392 IRIS_313-11393 IRIS_313-11394 IRIS_313-11395 IRIS_313-11396 IRIS_313-11397 IRIS_313-11399 IRIS_313-11400 IRIS_313-11401 IRIS_313-11402 IRIS_313-11406 IRIS_313-11407 IRIS_313-11408 IRIS_313-11409 IRIS_313-11411 IRIS_313-11413 IRIS_313-11414 IRIS_313-11416 IRIS_313-11418 IRIS_313-11420 IRIS_313-11422 IRIS_313-11423 IRIS_313-11424 IRIS_313-11425 IRIS_313-11426 IRIS_313-11427 IRIS_313-11428 IRIS_313-11429 IRIS_313-11431 IRIS_313-11433 IRIS_313-11434 IRIS_313-11435 IRIS_313-11436 IRIS_313-11438 IRIS_313-11439 IRIS_313-11442 IRIS_313-11446 IRIS_313-11448 IRIS_313-11449 IRIS_313-11451 IRIS_313-11453 IRIS_313-11460 IRIS_313-11461 IRIS_313-11467 IRIS_313-11471 IRIS_313-11478 IRIS_313-11480 IRIS_313-11481 IRIS_313-11490 IRIS_313-11491 IRIS_313-11493 IRIS_313-11494 IRIS_313-11495 IRIS_313-11496 IRIS_313-11497 IRIS_313-11499 IRIS_313-11500 IRIS_313-11505 IRIS_313-11506 IRIS_313-11507 IRIS_313-11508 IRIS_313-11510 IRIS_313-11513 IRIS_313-11515 IRIS_313-11516 IRIS_313-11517 IRIS_313-11521 IRIS_313-11522 IRIS_313-11524 IRIS_313-11525 IRIS_313-11526 IRIS_313-11527 IRIS_313-11528 IRIS_313-11530 IRIS_313-11536 IRIS_313-11538 IRIS_313-11539 IRIS_313-11540 IRIS_313-11541 IRIS_313-11542 IRIS_313-11543 IRIS_313-11544 IRIS_313-11545 IRIS_313-11546 IRIS_313-11548 IRIS_313-11549 IRIS_313-11551 IRIS_313-11554 IRIS_313-11555 IRIS_313-11556 IRIS_313-11558 IRIS_313-11561 IRIS_313-11563 IRIS_313-11565 IRIS_313-11566 IRIS_313-11568 IRIS_313-11571 IRIS_313-11572 IRIS_313-11573 IRIS_313-11574 IRIS_313-11575 IRIS_313-11576 IRIS_313-11577 IRIS_313-11579 IRIS_313-11580 IRIS_313-11582 IRIS_313-11585 IRIS_313-11586 IRIS_313-11588 IRIS_313-11596 IRIS_313-11597 IRIS_313-11598 IRIS_313-11600 IRIS_313-11606 IRIS_313-11607 IRIS_313-11608 IRIS_313-11609 IRIS_313-11610 IRIS_313-11621 IRIS_313-11622 IRIS_313-11623 IRIS_313-11624 IRIS_313-11626 IRIS_313-11627 IRIS_313-11635 IRIS_313-11638 IRIS_313-11639 IRIS_313-11640 IRIS_313-11641 IRIS_313-11642 IRIS_313-11645 IRIS_313-11646 IRIS_313-11648 IRIS_313-11650 IRIS_313-11651 IRIS_313-11652 IRIS_313-11653 IRIS_313-11654 IRIS_313-11655 IRIS_313-11656 IRIS_313-11657 IRIS_313-11658 IRIS_313-11659 IRIS_313-11660 IRIS_313-11661 IRIS_313-11663 IRIS_313-11664 IRIS_313-11666 IRIS_313-11667 IRIS_313-11668 IRIS_313-11669 IRIS_313-11671 IRIS_313-11672 IRIS_313-11673 IRIS_313-11674 IRIS_313-11677 IRIS_313-11679 IRIS_313-11680 IRIS_313-11684 IRIS_313-11687 IRIS_313-11688 IRIS_313-11689 IRIS_313-11690 IRIS_313-11691 IRIS_313-11692 IRIS_313-11693 IRIS_313-11694 IRIS_313-11698 IRIS_313-11700 IRIS_313-11702 IRIS_313-11705 IRIS_313-11706 IRIS_313-11708 IRIS_313-11709 IRIS_313-11710 IRIS_313-11712 IRIS_313-11713 IRIS_313-11714 IRIS_313-11716 IRIS_313-11717 IRIS_313-11722 IRIS_313-11723 IRIS_313-11724 IRIS_313-11725 IRIS_313-11726 IRIS_313-11727 IRIS_313-11728 IRIS_313-11729 IRIS_313-11730 IRIS_313-11731 IRIS_313-11732 IRIS_313-11733 IRIS_313-11734 IRIS_313-11738 IRIS_313-11739 IRIS_313-11741 IRIS_313-11743 IRIS_313-11744 IRIS_313-11745 IRIS_313-11746 IRIS_313-11747 IRIS_313-11748 IRIS_313-11750 IRIS_313-11751 IRIS_313-11752 IRIS_313-11753 IRIS_313-11754 IRIS_313-11755 IRIS_313-11756 IRIS_313-11757 IRIS_313-11759 IRIS_313-11760 IRIS_313-11761 IRIS_313-11762 IRIS_313-11763 IRIS_313-11764 IRIS_313-11766 IRIS_313-11767 IRIS_313-11772 IRIS_313-11782 IRIS_313-11783 IRIS_313-11784 IRIS_313-11786 IRIS_313-11787 IRIS_313-11789 IRIS_313-11791 IRIS_313-11793 IRIS_313-11794 IRIS_313-11795 IRIS_313-11796 IRIS_313-11797 IRIS_313-11798 IRIS_313-11799 IRIS_313-11800 IRIS_313-11801 IRIS_313-11802 IRIS_313-11803 IRIS_313-11804 IRIS_313-11805 IRIS_313-11806 IRIS_313-11807 IRIS_313-11808 IRIS_313-11811 IRIS_313-11812 IRIS_313-11813 IRIS_313-11816 IRIS_313-11818 IRIS_313-11819 IRIS_313-11821 IRIS_313-11822 IRIS_313-11823 IRIS_313-11824 IRIS_313-11825 IRIS_313-11827 IRIS_313-11829 IRIS_313-11830 IRIS_313-11832 IRIS_313-11833 IRIS_313-11834 IRIS_313-11835 IRIS_313-11836 IRIS_313-11838 IRIS_313-11839 IRIS_313-11840 IRIS_313-11841 IRIS_313-11842 IRIS_313-11844 IRIS_313-11845 IRIS_313-11846 IRIS_313-11848 IRIS_313-11850 IRIS_313-11852 IRIS_313-11853 IRIS_313-11854 IRIS_313-11855 IRIS_313-11856 IRIS_313-11857 IRIS_313-11858 IRIS_313-11859 IRIS_313-11860 IRIS_313-11861 IRIS_313-11862 IRIS_313-11865 IRIS_313-11866 IRIS_313-11867 IRIS_313-11868 IRIS_313-11869 IRIS_313-11870 IRIS_313-11871 IRIS_313-11872 IRIS_313-11875 IRIS_313-11876 IRIS_313-11877 IRIS_313-11878 IRIS_313-11881 IRIS_313-11882 IRIS_313-11884 IRIS_313-11885 IRIS_313-11886 IRIS_313-11888 IRIS_313-11889 IRIS_313-11890 IRIS_313-11892 IRIS_313-11893 IRIS_313-11894 IRIS_313-11895 IRIS_313-11896 IRIS_313-11897 IRIS_313-11899 IRIS_313-11900 IRIS_313-11901 IRIS_313-11905 IRIS_313-11906 IRIS_313-11907 IRIS_313-11908 IRIS_313-11910 IRIS_313-11911 IRIS_313-11913 IRIS_313-11914 IRIS_313-11915 IRIS_313-11918 IRIS_313-11919 IRIS_313-11920 IRIS_313-11922 IRIS_313-11923 IRIS_313-11924 IRIS_313-11925 IRIS_313-11926 IRIS_313-11928 IRIS_313-11930 IRIS_313-11931 IRIS_313-11932 IRIS_313-11934 IRIS_313-11937 IRIS_313-11938 IRIS_313-11939 IRIS_313-11940 IRIS_313-11941 IRIS_313-11943 IRIS_313-11944 IRIS_313-11945 IRIS_313-11947 IRIS_313-11948 IRIS_313-11949 IRIS_313-11951 IRIS_313-11952 IRIS_313-11953 IRIS_313-11954 IRIS_313-11957 IRIS_313-11958 IRIS_313-11960 IRIS_313-11962 IRIS_313-11964 IRIS_313-11965 IRIS_313-11966 IRIS_313-11968 IRIS_313-11969 IRIS_313-11970 IRIS_313-11971 IRIS_313-11973 IRIS_313-11974 IRIS_313-11975 IRIS_313-11976 IRIS_313-11977 IRIS_313-11978 IRIS_313-11979 IRIS_313-11980 IRIS_313-11981 IRIS_313-11982 IRIS_313-11983 IRIS_313-11984 IRIS_313-11986 IRIS_313-11987 IRIS_313-11988 IRIS_313-11989 IRIS_313-11990 IRIS_313-11993 IRIS_313-11994 IRIS_313-11995 IRIS_313-11997 IRIS_313-11998 IRIS_313-12000 IRIS_313-12003 IRIS_313-12004 IRIS_313-12005 IRIS_313-12006 IRIS_313-12009 IRIS_313-12010 IRIS_313-12011 IRIS_313-12013 IRIS_313-12014 IRIS_313-12015 IRIS_313-12016 IRIS_313-12018 IRIS_313-12021 IRIS_313-12024 IRIS_313-12028 IRIS_313-12030 IRIS_313-12033 IRIS_313-12037 IRIS_313-12038 IRIS_313-12039 IRIS_313-12042 IRIS_313-12045 IRIS_313-12046 IRIS_313-12047 IRIS_313-12051 IRIS_313-12052 IRIS_313-12053 IRIS_313-12054 IRIS_313-12059 IRIS_313-12060 IRIS_313-12061 IRIS_313-12063 IRIS_313-12065 IRIS_313-12066 IRIS_313-12067 IRIS_313-12068 IRIS_313-12069 IRIS_313-12070 IRIS_313-12071 IRIS_313-12072 IRIS_313-12073 IRIS_313-12074 IRIS_313-12076 IRIS_313-12077 IRIS_313-12079 IRIS_313-12080 IRIS_313-12081 IRIS_313-12082 IRIS_313-12083 IRIS_313-12093 IRIS_313-12096 IRIS_313-12108 IRIS_313-12109 IRIS_313-12118 IRIS_313-12129 IRIS_313-12131 IRIS_313-12133 IRIS_313-12134 IRIS_313-12135 IRIS_313-12138 IRIS_313-12141 IRIS_313-12142 IRIS_313-12143 IRIS_313-12146 IRIS_313-12152 IRIS_313-12164 IRIS_313-12180 IRIS_313-12182 IRIS_313-12185 IRIS_313-12186 IRIS_313-12190 IRIS_313-12194 IRIS_313-12200 IRIS_313-12207 IRIS_313-12210 IRIS_313-12217 IRIS_313-12220 IRIS_313-12222 IRIS_313-12225 IRIS_313-12226 IRIS_313-12227 IRIS_313-12229 IRIS_313-12232 IRIS_313-12234 IRIS_313-12236 IRIS_313-12240 IRIS_313-12241 IRIS_313-12242 IRIS_313-12244 IRIS_313-12247 IRIS_313-12249 IRIS_313-12251 IRIS_313-12252 IRIS_313-12254 IRIS_313-12257 IRIS_313-12258 IRIS_313-12259 IRIS_313-12261 IRIS_313-12262 IRIS_313-12263 IRIS_313-12265 IRIS_313-12266 IRIS_313-12269 IRIS_313-12270 IRIS_313-12271 IRIS_313-12273 IRIS_313-12275 IRIS_313-12278 IRIS_313-12279 IRIS_313-12280 IRIS_313-12281 IRIS_313-12282 IRIS_313-12283 IRIS_313-12284 IRIS_313-12285 IRIS_313-12286 IRIS_313-12288 IRIS_313-12289 IRIS_313-12290 IRIS_313-12291 IRIS_313-12292 IRIS_313-12296 IRIS_313-12297 IRIS_313-12299 IRIS_313-12300 IRIS_313-12307 IRIS_313-12312 IRIS_313-12313 IRIS_313-12319 IRIS_313-12321 IRIS_313-12322 IRIS_313-12323 IRIS_313-12324 IRIS_313-12325 IRIS_313-12329 IRIS_313-12330 IRIS_313-12332 IRIS_313-12336 IRIS_313-12340 IRIS_313-12341 IRIS_313-12342 IRIS_313-12345 IRIS_313-12346 IRIS_313-12347 IRIS_313-12348 IRIS_313-12350 IRIS_313-12351 IRIS_313-12353 IRIS_313-12354 IRIS_313-12355 IRIS_313-15897 IRIS_313-15898 IRIS_313-15899 IRIS_313-15900 IRIS_313-15901 IRIS_313-15904 IRIS_313-15905 IRIS_313-15906 IRIS_313-15907 IRIS_313-15910 IRIS_313-7620 IRIS_313-7635 IRIS_313-7636 IRIS_313-7646 IRIS_313-7650 IRIS_313-7651  IRIS_313-7654 IRIS_313-7664 IRIS_313-7665 IRIS_313-7668 IRIS_313-7681  IRIS_313-7684 IRIS_313-7685 IRIS_313-7688 IRIS_313-7689 IRIS_313-7690  IRIS_313-7691 IRIS_313-7696 IRIS_313-7698 IRIS_313-7699 IRIS_313-7719  IRIS_313-7720 IRIS_313-7722 IRIS_313-7725 IRIS_313-7728 IRIS_313-7736  IRIS_313-7766 IRIS_313-7769 IRIS_313-7770 IRIS_313-7773 IRIS_313-7778  IRIS_313-7780 IRIS_313-7793 IRIS_313-7795 IRIS_313-7797 IRIS_313-7799  IRIS_313-7807 IRIS_313-7808 IRIS_313-7815 IRIS_313-7816 IRIS_313-7819  IRIS_313-7820 IRIS_313-7824 IRIS_313-7826 IRIS_313-7832 IRIS_313-7838  IRIS_313-7850 IRIS_313-7856 IRIS_313-7859 IRIS_313-7863 IRIS_313-7866  IRIS_313-7868 IRIS_313-7870 IRIS_313-7883 IRIS_313-7885 IRIS_313-7902  IRIS_313-7907 IRIS_313-7909 IRIS_313-7911 IRIS_313-7912 IRIS_313-7914  IRIS_313-7922 IRIS_313-7924 IRIS_313-7933 IRIS_313-7959 IRIS_313-7992  IRIS_313-7993 IRIS_313-7994 IRIS_313-8003 IRIS_313-8010 IRIS_313-8011  IRIS_313-8023 IRIS_313-8024 IRIS_313-8025 IRIS_313-8026 IRIS_313-8029  IRIS_313-8031 IRIS_313-8033 IRIS_313-8037 IRIS_313-8039 IRIS_313-8041  IRIS_313-8046 IRIS_313-8050 IRIS_313-8052 IRIS_313-8057 IRIS_313-8058  IRIS_313-8060 IRIS_313-8061 IRIS_313-8062 IRIS_313-8063 IRIS_313-8065  IRIS_313-8067 IRIS_313-8068 IRIS_313-8069 IRIS_313-8072 IRIS_313-8074  IRIS_313-8075 IRIS_313-8076 IRIS_313-8084 IRIS_313-8085 IRIS_313-8087  IRIS_313-8090 IRIS_313-8095 IRIS_313-8096 IRIS_313-8097 IRIS_313-8099  IRIS_313-8102 IRIS_313-8105 IRIS_313-8109 IRIS_313-8111 IRIS_313-8112  IRIS_313-8113 IRIS_313-8114 IRIS_313-8115 IRIS_313-8116 IRIS_313-8118  IRIS_313-8119 IRIS_313-8121 IRIS_313-8123 IRIS_313-8124 IRIS_313-8125  IRIS_313-8126 IRIS_313-8127 IRIS_313-8128 IRIS_313-8129 IRIS_313-8132  IRIS_313-8134 IRIS_313-8135 IRIS_313-8136 IRIS_313-8137 IRIS_313-8138  IRIS_313-8139 IRIS_313-8140 IRIS_313-8141 IRIS_313-8142 IRIS_313-8143  IRIS_313-8145 IRIS_313-8147 IRIS_313-8148 IRIS_313-8149 IRIS_313-8151  IRIS_313-8154 IRIS_313-8155 IRIS_313-8158 IRIS_313-8159 IRIS_313-8160  IRIS_313-8161 IRIS_313-8162 IRIS_313-8164 IRIS_313-8165 IRIS_313-8166  IRIS_313-8168 IRIS_313-8171 IRIS_313-8177 IRIS_313-8185 IRIS_313-8186  IRIS_313-8192 IRIS_313-8193 IRIS_313-8195 IRIS_313-8200 IRIS_313-8204  IRIS_313-8205 IRIS_313-8208 IRIS_313-8209 IRIS_313-8212 IRIS_313-8213  IRIS_313-8214 IRIS_313-8215 IRIS_313-8217 IRIS_313-8218 IRIS_313-8232  IRIS_313-8244 IRIS_313-8265 IRIS_313-8268 IRIS_313-8277 IRIS_313-8279  IRIS_313-8283 IRIS_313-8285 IRIS_313-8293 IRIS_313-8303 IRIS_313-8305  IRIS_313-8306 IRIS_313-8312 IRIS_313-8315 IRIS_313-8316 IRIS_313-8323  IRIS_313-8324 IRIS_313-8339 IRIS_313-8341 IRIS_313-8349 IRIS_313-8356  IRIS_313-8368 IRIS_313-8380 IRIS_313-8381 IRIS_313-8386 IRIS_313-8387  IRIS_313-8391 IRIS_313-8392 IRIS_313-8398 IRIS_313-8399 IRIS_313-8400  IRIS_313-8405 IRIS_313-8412 IRIS_313-8431 IRIS_313-8433 IRIS_313-8434  IRIS_313-8435 IRIS_313-8436 IRIS_313-8437 IRIS_313-8450 IRIS_313-8453  IRIS_313-8454 IRIS_313-8457 IRIS_313-8458 IRIS_313-8466 IRIS_313-8468  IRIS_313-8481 IRIS_313-8486 IRIS_313-8493 IRIS_313-8519 IRIS_313-8523  IRIS_313-8530 IRIS_313-8536 IRIS_313-8557 IRIS_313-8565 IRIS_313-8568  IRIS_313-8572 IRIS_313-8578 IRIS_313-8580 IRIS_313-8594 IRIS_313-8595  IRIS_313-8599 IRIS_313-8603 IRIS_313-8606 IRIS_313-8614 IRIS_313-8616  IRIS_313-8621 IRIS_313-8622 IRIS_313-8626 IRIS_313-8627 IRIS_313-8632  IRIS_313-8637 IRIS_313-8638 IRIS_313-8643 IRIS_313-8645 IRIS_313-8657  IRIS_313-8658 IRIS_313-8659 IRIS_313-8660 IRIS_313-8664 IRIS_313-8665  IRIS_313-8679 IRIS_313-8681 IRIS_313-8683 IRIS_313-8687 IRIS_313-8690  IRIS_313-8694 IRIS_313-8697 IRIS_313-8702 IRIS_313-8703 IRIS_313-8704  IRIS_313-8713 IRIS_313-8717 IRIS_313-8721 IRIS_313-8723 IRIS_313-8725  IRIS_313-8727 IRIS_313-8731 IRIS_313-8732 IRIS_313-8733 IRIS_313-8735  IRIS_313-8737 IRIS_313-8739 IRIS_313-8743 IRIS_313-8745 IRIS_313-8747  IRIS_313-8751 IRIS_313-8754 IRIS_313-8755 IRIS_313-8757 IRIS_313-8765  IRIS_313-8767 IRIS_313-8768 IRIS_313-8769 IRIS_313-8778 IRIS_313-8781  IRIS_313-8785 IRIS_313-8793 IRIS_313-8796 IRIS_313-8803 IRIS_313-8811  IRIS_313-8812 IRIS_313-8813 IRIS_313-8814 IRIS_313-8815 IRIS_313-8822  IRIS_313-8831 IRIS_313-8844 IRIS_313-8846 IRIS_313-8854 IRIS_313-8856  IRIS_313-8857 IRIS_313-8859 IRIS_313-8865 IRIS_313-8870 IRIS_313-8872  IRIS_313-8876 IRIS_313-8883 IRIS_313-8884 IRIS_313-8889 IRIS_313-8890  IRIS_313-8894 IRIS_313-8895 IRIS_313-8900 IRIS_313-8903 IRIS_313-8909  IRIS_313-8914 IRIS_313-8921 IRIS_313-8922 IRIS_313-8923 IRIS_313-8924  IRIS_313-8925 IRIS_313-8930 IRIS_313-8932 IRIS_313-8935 IRIS_313-8940  IRIS_313-8955 IRIS_313-8956 IRIS_313-8957 IRIS_313-8960 IRIS_313-8967  IRIS_313-8968 IRIS_313-8976 IRIS_313-8978 IRIS_313-8982 IRIS_313-8986  IRIS_313-8987 IRIS_313-8988 IRIS_313-8999 IRIS_313-9002 IRIS_313-9006  IRIS_313-9017 IRIS_313-9019 IRIS_313-9020 IRIS_313-9023 IRIS_313-9032  IRIS_313-9039 IRIS_313-9048 IRIS_313-9049 IRIS_313-9054 IRIS_313-9065  IRIS_313-9066 IRIS_313-9067 IRIS_313-9070 IRIS_313-9072 IRIS_313-9081 IRIS_313-9098 IRIS_313-9108 IRIS_313-9114 IRIS_313-9121 IRIS_313-9123 IRIS_313-9129 IRIS_313-9140 IRIS_313-9148 IRIS_313-9156 IRIS_313-9160 IRIS_313-9170 IRIS_313-9172 IRIS_313-9174 IRIS_313-9176 IRIS_313-9182 IRIS_313-9184 IRIS_313-9187 IRIS_313-9193 IRIS_313-9197 IRIS_313-9198 IRIS_313-9201 IRIS_313-9204 IRIS_313-9209 IRIS_313-9210 IRIS_313-9218 IRIS_313-9227 IRIS_313-9228 IRIS_313-9239 IRIS_313-9249 IRIS_313-9251 IRIS_313-9253 IRIS_313-9258 IRIS_313-9259 IRIS_313-9262 IRIS_313-9273 IRIS_313-9281 IRIS_313-9285 IRIS_313-9286 IRIS_313-9287 IRIS_313-9288 IRIS_313-9294 IRIS_313-9297 IRIS_313-9301 IRIS_313-9302 IRIS_313-9310 IRIS_313-9313 IRIS_313-9314 IRIS_313-9317 IRIS_313-9320 IRIS_313-9324 IRIS_313-9325 IRIS_313-9329 IRIS_313-9342 IRIS_313-9346 IRIS_313-9347 IRIS_313-9348 IRIS_313-9351 IRIS_313-9357 IRIS_313-9360 IRIS_313-9363 IRIS_313-9366 IRIS_313-9368 IRIS_313-9372 IRIS_313-9375 IRIS_313-9379 IRIS_313-9384 IRIS_313-9389 IRIS_313-9391 IRIS_313-9392 IRIS_313-9397 IRIS_313-9400 IRIS_313-9402 IRIS_313-9403 IRIS_313-9405 IRIS_313-9406 IRIS_313-9409 IRIS_313-9410 IRIS_313-9415 IRIS_313-9423 IRIS_313-9424 IRIS_313-9427 IRIS_313-9429 IRIS_313-9438 IRIS_313-9445 IRIS_313-9449 IRIS_313-9451 IRIS_313-9452 IRIS_313-9461 IRIS_313-9464 IRIS_313-9468 IRIS_313-9469 IRIS_313-9482 IRIS_313-9484 IRIS_313-9491 IRIS_313-9492 IRIS_313-9503 IRIS_313-9505 IRIS_313-9506 IRIS_313-9519 IRIS_313-9522 IRIS_313-9523 IRIS_313-9529 IRIS_313-9533 IRIS_313-9539 IRIS_313-9550 IRIS_313-9551 IRIS_313-9555 IRIS_313-9557 IRIS_313-9566 IRIS_313-9567 IRIS_313-9570 IRIS_313-9574 IRIS_313-9575 IRIS_313-9582 IRIS_313-9590 IRIS_313-9593 IRIS_313-9594 IRIS_313-9600 IRIS_313-9602 IRIS_313-9604 IRIS_313-9605 IRIS_313-9606 IRIS_313-9609 IRIS_313-9611 IRIS_313-9617 IRIS_313-9619 IRIS_313-9626 IRIS_313-9636 IRIS_313-9641 IRIS_313-9648 IRIS_313-9661 IRIS_313-9682 IRIS_313-9685 IRIS_313-9691 IRIS_313-9694 IRIS_313-9695 IRIS_313-9696 IRIS_313-9697 IRIS_313-9698 IRIS_313-9699 IRIS_313-9701 IRIS_313-9702 IRIS_313-9703 IRIS_313-9705 IRIS_313-9706 IRIS_313-9708 IRIS_313-9709 IRIS_313-9723 IRIS_313-9724 IRIS_313-9725 IRIS_313-9727 IRIS_313-9730 IRIS_313-9732 IRIS_313-9740 IRIS_313-9742 IRIS_313-9745 IRIS_313-9758 IRIS_313-9759 IRIS_313-9767 IRIS_313-9769 IRIS_313-9770 IRIS_313-9771 IRIS_313-9774 IRIS_313-9778 IRIS_313-9782 IRIS_313-9783 IRIS_313-9789 IRIS_313-9790 IRIS_313-9791 IRIS_313-9795 IRIS_313-9809 IRIS_313-9811 IRIS_313-9813 IRIS_313-9814 IRIS_313-9817 IRIS_313-9818 IRIS_313-9822 IRIS_313-9825 IRIS_313-9832 IRIS_313-9838 IRIS_313-9839 IRIS_313-9841 IRIS_313-9851 IRIS_313-9867 IRIS_313-9884 IRIS_313-9886 IRIS_313-9887 IRIS_313-9890 IRIS_313-9891 IRIS_313-9897 IRIS_313-9917 IRIS_313-9922 IRIS_313-9924 IRIS_313-9925 IRIS_313-9926 IRIS_313-9928 IRIS_313-9935 IRIS_313-9936 IRIS_313-9937 IRIS_313-9939 IRIS_313-9940 IRIS_313-9944 IRIS_313-9949 IRIS_313-9953 IRIS_313-9964 IRIS_313-9966 IRIS_313-9968 IRIS_313-9969 IRIS_313-9970 IRIS_313-9974 IRIS_313-9976 IRIS_313-9980 IRIS_313-9986 IRIS_313-9995 IRIS_313-9996 | 2,296 |
| Hap_2 | B014 | 1 |
| Hap_3 | B023 | 1 |
| Hap_4 | B025 | 1 |
| Hap_5 | B034 | 1 |
| Hap_6 | B045 | 1 |
| Hap_7 | B049 B164 B221 B243 CX108 CX367 CX368 CX399 IRIS_313-10025 IRIS_313-10150 IRIS_313-10593 IRIS_313-10598 IRIS_313-10602 IRIS_313-10623 IRIS_313-10734 IRIS_313-10735 IRIS_313-10736 IRIS_313-10737 IRIS_313-10852 IRIS_313-10854 IRIS_313-10856 IRIS_313-10859 IRIS_313-10861 IRIS_313-10871 IRIS_313-10875 IRIS_313-10876 IRIS_313-10877 IRIS_313-10878 IRIS_313-10891 IRIS_313-10925 IRIS_313-10927 IRIS_313-10930 IRIS_313-10964 IRIS_313-10965 IRIS_313-10969 IRIS_313-10976 IRIS_313-10981 IRIS_313-11016 IRIS_313-11017 IRIS_313-11018 IRIS_313-11019 IRIS_313-11029 IRIS_313-11047 IRIS_313-11050 IRIS_313-11052 IRIS_313-11055 IRIS_313-11056 IRIS_313-11063 IRIS_313-11064 IRIS_313-11065 IRIS_313-11067 IRIS_313-11168 IRIS_313-11172 IRIS_313-11173 IRIS_313-11174 IRIS_313-11175 IRIS_313-11176 IRIS_313-11213 IRIS_313-11216 IRIS_313-11232 IRIS_313-11265 IRIS_313-11286 IRIS_313-11298 IRIS_313-11306 IRIS_313-11323 IRIS_313-11324 IRIS_313-11348 IRIS_313-11353 IRIS_313-11371 IRIS_313-11432 IRIS_313-11455 IRIS_313-11456 IRIS_313-11458 IRIS_313-11462 IRIS_313-11476 IRIS_313-11477 IRIS_313-11483 IRIS_313-11489 IRIS_313-11492 IRIS_313-11595 IRIS_313-11602 IRIS_313-11603 IRIS_313-11604 IRIS_313-11616 IRIS_313-11617 IRIS_313-11619 IRIS_313-11620 IRIS_313-11625 IRIS_313-11628 IRIS_313-11629 IRIS_313-11636 IRIS_313-11643 IRIS_313-11647 IRIS_313-11737 IRIS_313-11742 IRIS_313-11809 IRIS_313-11917 IRIS_313-11963 IRIS_313-12002 IRIS_313-12139 IRIS_313-12183 IRIS_313-15908 IRIS_313-7792 IRIS_313-8073 IRIS_313-8252 IRIS_313-8302 IRIS_313-8321 IRIS_313-8332 IRIS_313-8382 IRIS_313-8390 IRIS_313-8410 IRIS_313-8554 IRIS_313-8641 IRIS_313-8655 IRIS_313-8771 IRIS_313-8783 IRIS_313-8789 IRIS_313-8864 IRIS_313-8963 IRIS_313-9044 IRIS_313-9137 IRIS_313-9283 IRIS_313-9422 IRIS_313-9572 IRIS_313-9861 | 125 |
| Hap_8 | B065 B082 B083 B113 B120 B129 B184 B227 B253 B254 CX124 CX148 CX157 CX23 CX304 CX61 CX64 IRIS_313-10050 IRIS_313-10177 IRIS_313-10287 IRIS_313-10337 IRIS_313-10423 IRIS_313-10441 IRIS_313-10480 IRIS_313-10510 IRIS_313-10513 IRIS_313-10523 IRIS_313-10525 IRIS_313-10544 IRIS_313-10572 IRIS_313-10610 IRIS_313-10659 IRIS_313-10666 IRIS_313-10681 IRIS_313-10682 IRIS_313-10683 IRIS_313-10692 IRIS_313-10697 IRIS_313-10700 IRIS_313-10715 IRIS_313-10719 IRIS_313-10742 IRIS_313-10751 IRIS_313-10760 IRIS_313-10762 IRIS_313-10768 IRIS_313-10792 IRIS_313-10820 IRIS_313-10821 IRIS_313-10822 IRIS_313-10833 IRIS_313-10855 IRIS_313-10887 IRIS_313-10907 IRIS_313-10913 IRIS_313-10934 IRIS_313-10935 IRIS_313-10938 IRIS_313-10955 IRIS_313-10962 IRIS_313-10966 IRIS_313-10990 IRIS_313-11010 IRIS_313-11042 IRIS_313-11043 IRIS_313-11071 IRIS_313-11076 IRIS_313-11083 IRIS_313-11090 IRIS_313-11095 IRIS_313-11097 IRIS_313-11110 IRIS_313-11132 IRIS_313-11133 IRIS_313-11135 IRIS_313-11151 IRIS_313-11179 IRIS_313-11180 IRIS_313-11181 IRIS_313-11182 IRIS_313-11196 IRIS_313-11235 IRIS_313-11252 IRIS_313-11276 IRIS_313-11296 IRIS_313-11304 IRIS_313-11315 IRIS_313-11318 IRIS_313-11330 IRIS_313-11331 IRIS_313-11338 IRIS_313-11343 IRIS_313-11347 IRIS_313-11377 IRIS_313-11381 IRIS_313-11383 IRIS_313-11384 IRIS_313-11388 IRIS_313-11405 IRIS_313-11410 IRIS_313-11437 IRIS_313-11447 IRIS_313-11463 IRIS_313-11485 IRIS_313-11512 IRIS_313-11534 IRIS_313-11535 IRIS_313-11547 IRIS_313-11570 IRIS_313-11591 IRIS_313-11594 IRIS_313-11644 IRIS_313-11676 IRIS_313-11682 IRIS_313-11685 IRIS_313-11686 IRIS_313-11695 IRIS_313-11707 IRIS_313-11719 IRIS_313-11721 IRIS_313-11735 IRIS_313-11740 IRIS_313-11758 IRIS_313-11902 IRIS_313-11904 IRIS_313-11912 IRIS_313-11916 IRIS_313-11921 IRIS_313-11927 IRIS_313-11950 IRIS_313-11955 IRIS_313-11959 IRIS_313-11961 IRIS_313-11967 IRIS_313-11985 IRIS_313-12007 IRIS_313-12017 IRIS_313-12027 IRIS_313-12048 IRIS_313-12049 IRIS_313-12057 IRIS_313-12078 IRIS_313-12127 IRIS_313-12128 IRIS_313-12130 IRIS_313-12147 IRIS_313-12187 IRIS_313-12221 IRIS_313-12224 IRIS_313-12231 IRIS_313-12246 IRIS_313-12287 IRIS_313-12301 IRIS_313-12302 IRIS_313-12303 IRIS_313-12305 IRIS_313-12308 IRIS_313-12309 IRIS_313-12334 IRIS_313-15902 IRIS_313-7638 IRIS_313-7641 IRIS_313-8291 IRIS_313-8292 IRIS_313-8407 IRIS_313-8409 IRIS_313-8492 IRIS_313-8559 IRIS_313-8567 IRIS_313-8586 IRIS_313-8591 IRIS_313-8608 IRIS_313-8631 IRIS_313-8699 IRIS_313-8744 IRIS_313-8791 IRIS_313-8833 IRIS_313-8873 IRIS_313-8880 IRIS_313-8946 IRIS_313-9062 IRIS_313-9091 IRIS_313-9097 IRIS_313-9102 IRIS_313-9112 IRIS_313-9115 IRIS_313-9116 IRIS_313-9119 IRIS_313-9131 IRIS_313-9139 IRIS_313-9188 IRIS_313-9190 IRIS_313-9208 IRIS_313-9243 IRIS_313-9256 IRIS_313-9271 IRIS_313-9623 IRIS_313-9669 IRIS_313-9882 | 199 |
| Hap_9 | B076 | 1 |
| Hap_10 | B077 | 1 |
| Hap_11 | B096 | 1 |
| Hap_12 | B101 | 1 |
| Hap_13 | B102 | 1 |
| Hap_14 | B110 B111 B123 CX104 CX112 CX182 CX266 CX281 CX341 CX355 CX362 CX363 CX365 CX392 CX43 CX46 CX53 CX59 CX65 CX66 CX72 IRIS_313-10032 IRIS_313-10134 IRIS_313-10147 IRIS_313-10151 IRIS_313-10555 IRIS_313-10578 IRIS_313-10732 IRIS_313-10758 IRIS_313-10782 IRIS_313-10812 IRIS_313-10850 IRIS_313-10872 IRIS_313-10910 IRIS_313-10918 IRIS_313-10921 IRIS_313-10922 IRIS_313-10960 IRIS_313-10961 IRIS_313-10994 IRIS_313-11021 IRIS_313-11023 IRIS_313-11045 IRIS_313-11062 IRIS_313-11066 IRIS_313-11070 IRIS_313-11079 IRIS_313-11080 IRIS_313-11178 IRIS_313-11218 IRIS_313-11300 IRIS_313-11340 IRIS_313-11350 IRIS_313-11356 IRIS_313-11362 IRIS_313-11417 IRIS_313-11464 IRIS_313-11473 IRIS_313-11487 IRIS_313-11509 IRIS_313-11532 IRIS_313-11537 IRIS_313-11567 IRIS_313-11611 IRIS_313-11630 IRIS_313-11632 IRIS_313-11711 IRIS_313-11720 IRIS_313-11736 IRIS_313-11765 IRIS_313-11780 IRIS_313-11785 IRIS_313-11810 IRIS_313-11814 IRIS_313-11815 IRIS_313-11820 IRIS_313-11826 IRIS_313-11831 IRIS_313-11837 IRIS_313-11929 IRIS_313-11946 IRIS_313-11996 IRIS_313-12094 IRIS_313-12121 IRIS_313-12144 IRIS_313-12188 IRIS_313-12272 IRIS_313-12337 IRIS_313-12349 IRIS_313-12352 IRIS_313-15909 IRIS_313-7809 IRIS_313-7876 IRIS_313-8172 IRIS_313-8173 IRIS_313-8180 IRIS_313-8184 IRIS_313-8326 IRIS_313-8439 IRIS_313-8571 IRIS_313-8656 IRIS_313-8669 IRIS_313-8980 IRIS_313-8996 IRIS_313-8998 IRIS_313-9005 IRIS_313-9101 IRIS_313-9470 IRIS_313-9558 IRIS_313-9568 IRIS_313-9629 IRIS_313-9989 | 112 |
| Hap_15 | B133 | 1 |
| Hap_16 | B137 B255 CX315 CX360 CX371 CX69 IRIS_313-10279 IRIS_313-10403 IRIS_313-10521 IRIS_313-10531 IRIS_313-10649 IRIS_313-10705 IRIS_313-10874 IRIS_313-10919 IRIS_313-10920 IRIS_313-10992 IRIS_313-10993 IRIS_313-11026 IRIS_313-11030 IRIS_313-11117 IRIS_313-11143 IRIS_313-11278 IRIS_313-11327 IRIS_313-11329 IRIS_313-11373 IRIS_313-11403 IRIS_313-11421 IRIS_313-11441 IRIS_313-11465 IRIS_313-11511 IRIS_313-11564 IRIS_313-11851 IRIS_313-11903 IRIS_313-11992 IRIS_313-12029 IRIS_313-12031 IRIS_313-12050 IRIS_313-12344 IRIS_313-8256 IRIS_313-8385 IRIS_313-8485 IRIS_313-9050 IRIS_313-9120 IRIS_313-9601 IRIS_313-9616 IRIS_313-9898 IRIS_313-9929 IRIS_313-9978 | 48 |
| Hap_17 | B141 | 1 |
| Hap_18 | B150 IRIS_313-9404 | 2 |
| Hap_19 | B154 | 1 |
| Hap_20 | B185 | 1 |
| Hap_21 | B203 | 1 |
| Hap_22 | B252 | 1 |
| Hap_23 | CX100 | 1 |
| Hap_24 | CX120 | 1 |
| Hap_25 | CX122 | 1 |
| Hap_26 | CX212 | 1 |
| Hap_27 | CX400 CX401 CX402 IRIS_313-10729 IRIS_313-11159 IRIS_313-11415 | 6 |
| Hap_28 | CX67 | 1 |
| Hap_29 | IRIS_313-10016 | 1 |
| Hap_30 | IRIS_313-10057 | 1 |
| Hap_31 | IRIS_313-10075 | 1 |
| Hap_32 | IRIS_313-10294 | 1 |
| Hap_33 | IRIS_313-10332 | 1 |
| Hap_34 | IRIS_313-10397 | 1 |
| Hap_35 | IRIS_313-10440 IRIS_313-11788 | 2 |
| Hap_36 | IRIS_313-10549 | 1 |
| Hap_37 | IRIS_313-10557 IRIS_313-10725 IRIS_313-10897 IRIS_313-10898 IRIS_313-10901 IRIS_313-10911 IRIS_313-10970 IRIS_313-11072 IRIS_313-11088 IRIS_313-11341 IRIS_313-11472 IRIS_313-11523 IRIS_313-11615 IRIS_313-11683 IRIS_313-11773 IRIS_313-11843 IRIS_313-11936 IRIS_313-11999 IRIS_313-12036 IRIS_313-12040 IRIS_313-12041 IRIS_313-12043 IRIS_313-12044 IRIS_313-12097 IRIS_313-12101 IRIS_313-12102 IRIS_313-12151 IRIS_313-12161 IRIS_313-8948 IRIS_313-9687 | 30 |
| Hap_38 | IRIS_313-10569 | 1 |
| Hap_39 | IRIS_313-10574 IRIS_313-11387 IRIS_313-11681 IRIS_313-8911 | 4 |
| Hap_40 | IRIS_313-10595 | 1 |
| Hap_41 | IRIS_313-10662 | 1 |
| Hap_42 | IRIS_313-10711 | 1 |
| Hap_43 | IRIS_313-10720 | 1 |
| Hap_44 | IRIS_313-10722 | 1 |
| Hap_45 | IRIS_313-10733 IRIS_313-11126 IRIS_313-11287 IRIS_313-11443 IRIS_313-12268 IRIS_313-8401 IRIS_313-8585 IRIS_313-8994 IRIS_313-9560 IRIS_313-9634 | 10 |
| Hap_46 | IRIS_313-10780 IRIS_313-10800 IRIS_313-10803 IRIS_313-11479 IRIS_313-11498 IRIS_313-11790 IRIS_313-8314 | 7 |
| Hap_47 | IRIS_313-10788 | 1 |
| Hap_48 | IRIS_313-10825 | 1 |
| Hap_49 | IRIS_313-10849 | 1 |
| Hap_50 | IRIS_313-10868 | 1 |
| Hap_51 | IRIS_313-10869 IRIS_313-11027 IRIS_313-11061 IRIS_313-11243 IRIS_313-11274 IRIS_313-11291 IRIS_313-11452 IRIS_313-11618 IRIS_313-8342 | 9 |
| Hap_52 | IRIS_313-10899 IRIS_313-10900 | 2 |
| Hap_53 | IRIS_313-10979 IRIS_313-11112 IRIS_313-11116 IRIS_313-11482 IRIS_313-11484 | 5 |
| Hap_54 | IRIS_313-10985 | 1 |
| Hap_55 | IRIS_313-11011 | 1 |
| Hap_56 | IRIS_313-11012 | 1 |
| Hap_57 | IRIS_313-11014 | 1 |
| Hap_58 | IRIS_313-11068 | 1 |
| Hap_59 | IRIS_313-11069 | 1 |
| Hap_60 | IRIS_313-11144 | 1 |
| Hap_61 | IRIS_313-11154 | 1 |
| Hap_62 | IRIS_313-11164 | 1 |
| Hap_63 | IRIS_313-11166 | 1 |
| Hap_64 | IRIS_313-11210 | 1 |
| Hap_65 | IRIS_313-11222 | 1 |
| Hap_66 | IRIS_313-11269 | 1 |
| Hap_67 | IRIS_313-11283 | 1 |
| Hap_68 | IRIS_313-11284 | 1 |
| Hap_69 | IRIS_313-11352 | 1 |
| Hap_70 | IRIS_313-11398 | 1 |
| Hap_71 | IRIS_313-11404 | 1 |
| Hap_72 | IRIS_313-11419 | 1 |
| Hap_73 | IRIS_313-11445 | 1 |
| Hap_74 | IRIS_313-11454 | 1 |
| Hap_75 | IRIS_313-11486 | 1 |
| Hap_76 | IRIS_313-11557 | 1 |
| Hap_77 | IRIS_313-11578 | 1 |
| Hap_78 | IRIS_313-11584 | 1 |
| Hap_79 | IRIS_313-11599 | 1 |
| Hap_80 | IRIS_313-11665 | 1 |
| Hap_81 | IRIS_313-11678 | 1 |
| Hap_82 | IRIS_313-11704 IRIS_313-11849 IRIS_313-12148 IRIS_313-9637 | 4 |
| Hap_83 | IRIS_313-11779 | 1 |
| Hap_84 | IRIS_313-11792 | 1 |
| Hap_85 | IRIS_313-11817 | 1 |
| Hap_86 | IRIS_313-11828 | 1 |
| Hap_87 | IRIS_313-11863 IRIS_313-11909 IRIS_313-12237 IRIS_313-9111 | 4 |
| Hap_88 | IRIS_313-11887 | 1 |
| Hap_89 | IRIS_313-11898 | 1 |
| Hap_90 | IRIS_313-11935 | 1 |
| Hap_91 | IRIS_313-11956 | 1 |
| Hap_92 | IRIS_313-11991 | 1 |
| Hap_93 | IRIS_313-12012 | 1 |
| Hap_94 | IRIS_313-12055 | 1 |
| Hap_95 | IRIS_313-12058 IRIS_313-8985 | 2 |
| Hap_96 | IRIS_313-12193 | 1 |
| Hap_97 | IRIS_313-12228 | 1 |
| Hap_98 | IRIS_313-12260 | 1 |
| Hap_99 | IRIS_313-12311 | 1 |
| Hap_100 | IRIS_313-15896 | 1 |
| Hap_101 | IRIS_313-15903 | 1 |
| Hap_102 | IRIS_313-7758 | 1 |
| Hap_103 | IRIS_313-8027 | 1 |
| Hap_104 | IRIS_313-8032 | 1 |
| Hap_105 | IRIS_313-8044 | 1 |
| Hap_106 | IRIS_313-8048 | 1 |
| Hap_107 | IRIS_313-8049 | 1 |
| Hap_108 | IRIS_313-8053 | 1 |
| Hap_109 | IRIS_313-8064 | 1 |
| Hap_110 | IRIS_313-8066 | 1 |
| Hap_111 | IRIS_313-8167 | 1 |
| Hap_112 | IRIS_313-8170 | 1 |
| Hap_113 | IRIS_313-8183 | 1 |
| Hap_114 | IRIS_313-8202 | 1 |
| Hap_115 | IRIS_313-8216 | 1 |
| Hap_116 | IRIS_313-8253 IRIS_313-8674 | 2 |
| Hap_117 | IRIS_313-8288 | 1 |
| Hap_118 | IRIS_313-8383 | 1 |
| Hap_119 | IRIS_313-8414 | 1 |
| Hap_120 | IRIS_313-8444 | 1 |
| Hap_121 | IRIS_313-8474 | 1 |
| Hap_122 | IRIS_313-8498 | 1 |
| Hap_123 | IRIS_313-8502 | 1 |
| Hap_124 | IRIS_313-8509 | 1 |
| Hap_125 | IRIS_313-8514 | 1 |
| Hap_126 | IRIS_313-8647 | 1 |
| Hap_127 | IRIS_313-8712 | 1 |
| Hap_128 | IRIS_313-8722 | 1 |
| Hap_129 | IRIS_313-8850 | 1 |
| Hap_130 | IRIS_313-8920 | 1 |
| Hap_131 | IRIS_313-8927 | 1 |
| Hap_132 | IRIS_313-9053 | 1 |
| Hap_133 | IRIS_313-9083 | 1 |
| Hap_134 | IRIS_313-9117 | 1 |
| Hap_135 | IRIS_313-9233 | 1 |
| Hap_136 | IRIS_313-9242 | 1 |
| Hap_137 | IRIS_313-9267 | 1 |
| Hap_138 | IRIS_313-9388 | 1 |
| Hap_139 | IRIS_313-9433 | 1 |
| Hap_140 | IRIS_313-9463 | 1 |
| Hap_141 | IRIS_313-9472 | 1 |
| Hap_142 | IRIS_313-9516 | 1 |
| Hap_143 | IRIS_313-9547 | 1 |
| Hap_144 | IRIS_313-9610 | 1 |
| Hap_145 | IRIS_313-9800 | 1 |
| Hap_146 | IRIS_313-9831 | 1 |
| Hap_147 | IRIS_313-9862 | 1 |
| Hap_148 | IRIS_313-9880 | 1 |
| Hap_149 | IRIS_313-9961 | 1 |
| Hap_150 | IRIS_313-9963 | 1 |
| **Total** |  | **3,000** |

Yellow box -Novel functional haplotypes

**Table S8.** Details of functional SNPS in *BADH2* coding region of 3000 accession set. (Excel file)

**Table S9.** List of *BADH1* haplotypes in 421 cultivated accessions. (Excel file)

**Table S10.** Markers identified in association analysis between the fragrance and *BADH1* gene region. (Excel file)

**Table S11.** Primer sets used for validation the *BADH2* functional novel alleles.

| **No.** | **Name** | **Sequence 5' to 3'** | **Length (bp)** | **Tm** | **Size (bp)** |
| --- | --- | --- | --- | --- | --- |
| 1 | P476_E2C>A_F | GAGGCGCTGAAGAGGAACC | 19 | 60 | 159 |
|  | P476_E2C>A_R | TGTGTTCCGTACAGGGGG | 19 | 60 |  |
| 2 | P440_E2C>G_F | GAGGCGCTGAAGAGGAACC | 19 | 60 | 77 |
|  | P440_E2C>G_R | ATTGCGCGGAGGTACTTG | 18 | 60 |  |
| 3 | P4460_E10G>A_F | GGCATGAAGCAACTTTGAACT | 21 | 59 | 171 |
|  | P4460_E10G>A_R | AATATTTTTGGCCCATGCAA | 20 | 60 |  |
| 4 | P5433_E13A>T_F | GTTATGGTCTGGCTGGTGCT | 20 | 60 | 184 |
|  | P5433_E13A>T_R | GGAGTCCAGGGAAGAACTGG | 20 | 61 |  |

**Fig. S1.** Comparison between 2AP biosynthesis pathway of aromatic rice and non-aromatic rice.


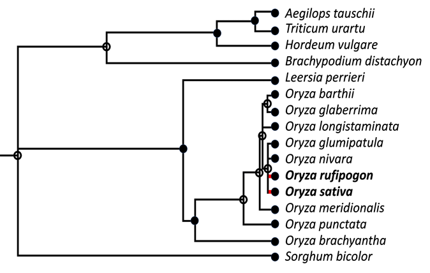


**Fig. S2.** Phylogenetic analysis of *BADH2* orthologs from plants.


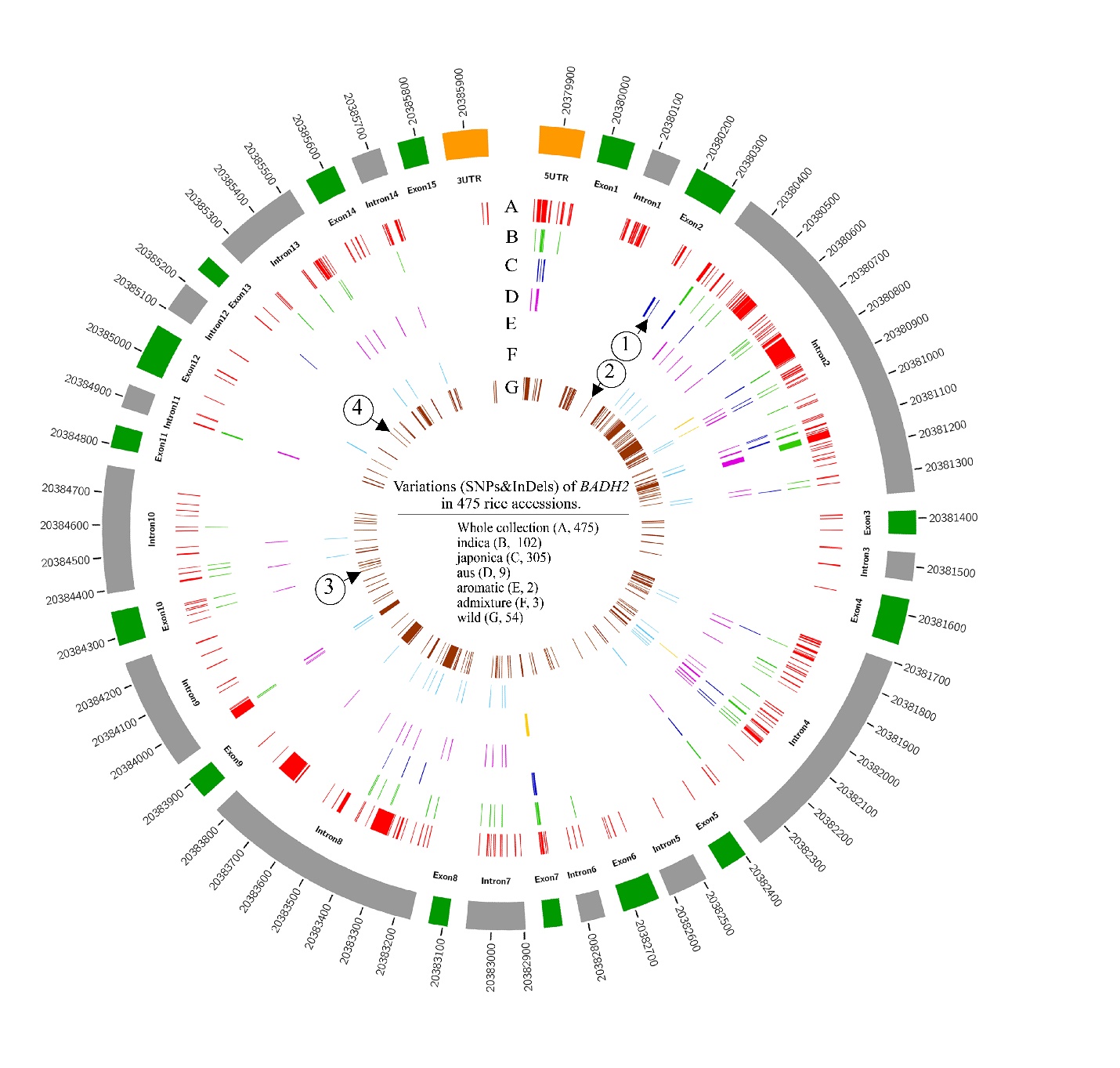


**Fig. S3.** Distribution of the nucleotide variations (SNPs and InDels) in the BADH2 gene of 475 rice accessions. The lines on the circle map indicate SNP/InDel positions. Total variations detected in 475 accessions (**A**), and groups-indica (**B**), japonica (**C**), aus (**D**), aromatic (**E**), admixture (**F**), and (**G**) wild. The numbers in circles indicate the four novel positions identified in this study. The position on chromosome 8 indicated in bp on outside. The number at center indicates the number of accessions from each group.


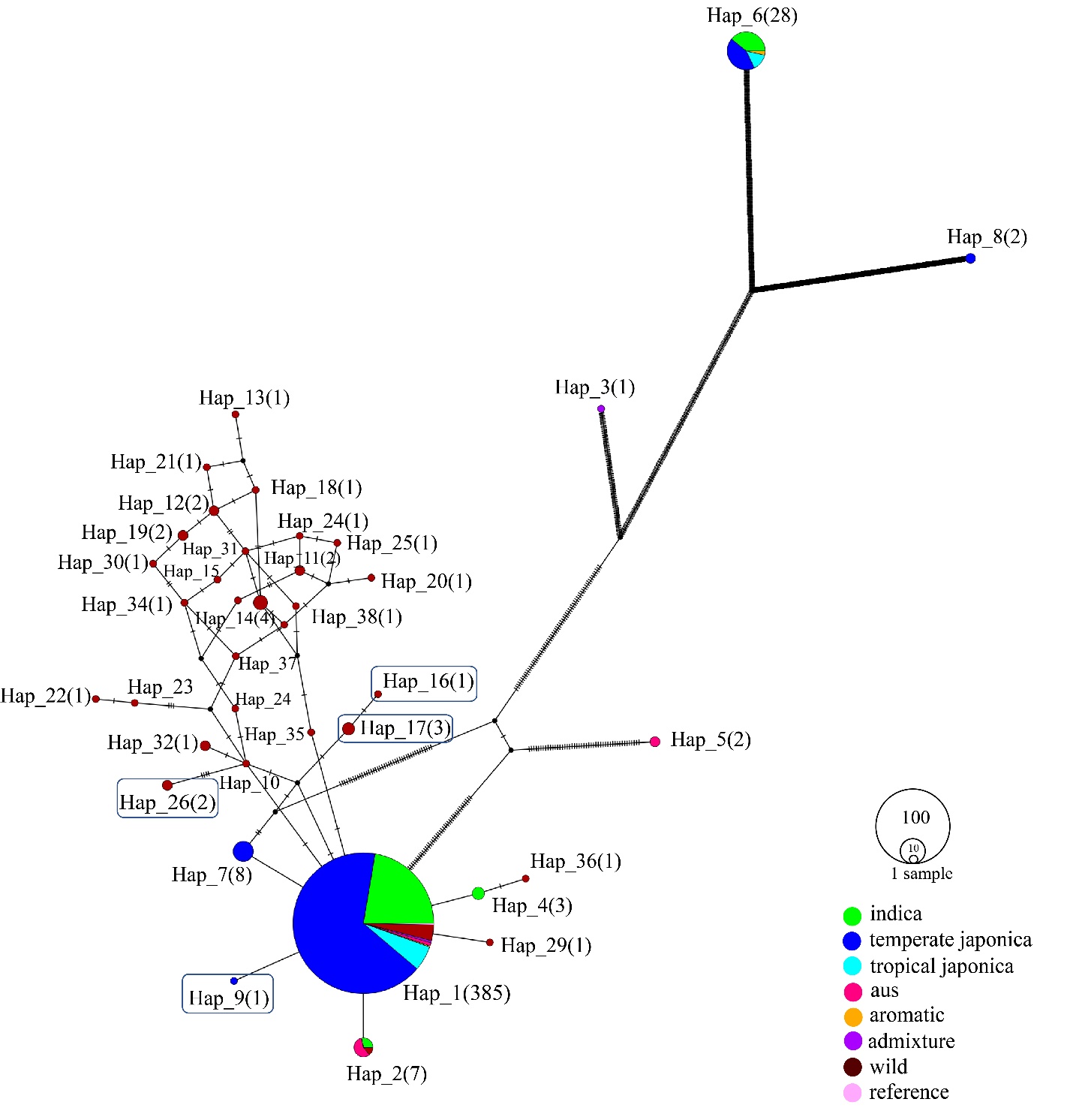


**Fig. S4.** Haplotype network of the *BADH2* gene from 475 rice accessions. The circle size is proportional to the number of samples and ecotypes, the four haplotypes in rectangles are newly identified haplotypes, and the dashes between haplotypes represent mutational steps between alleles.


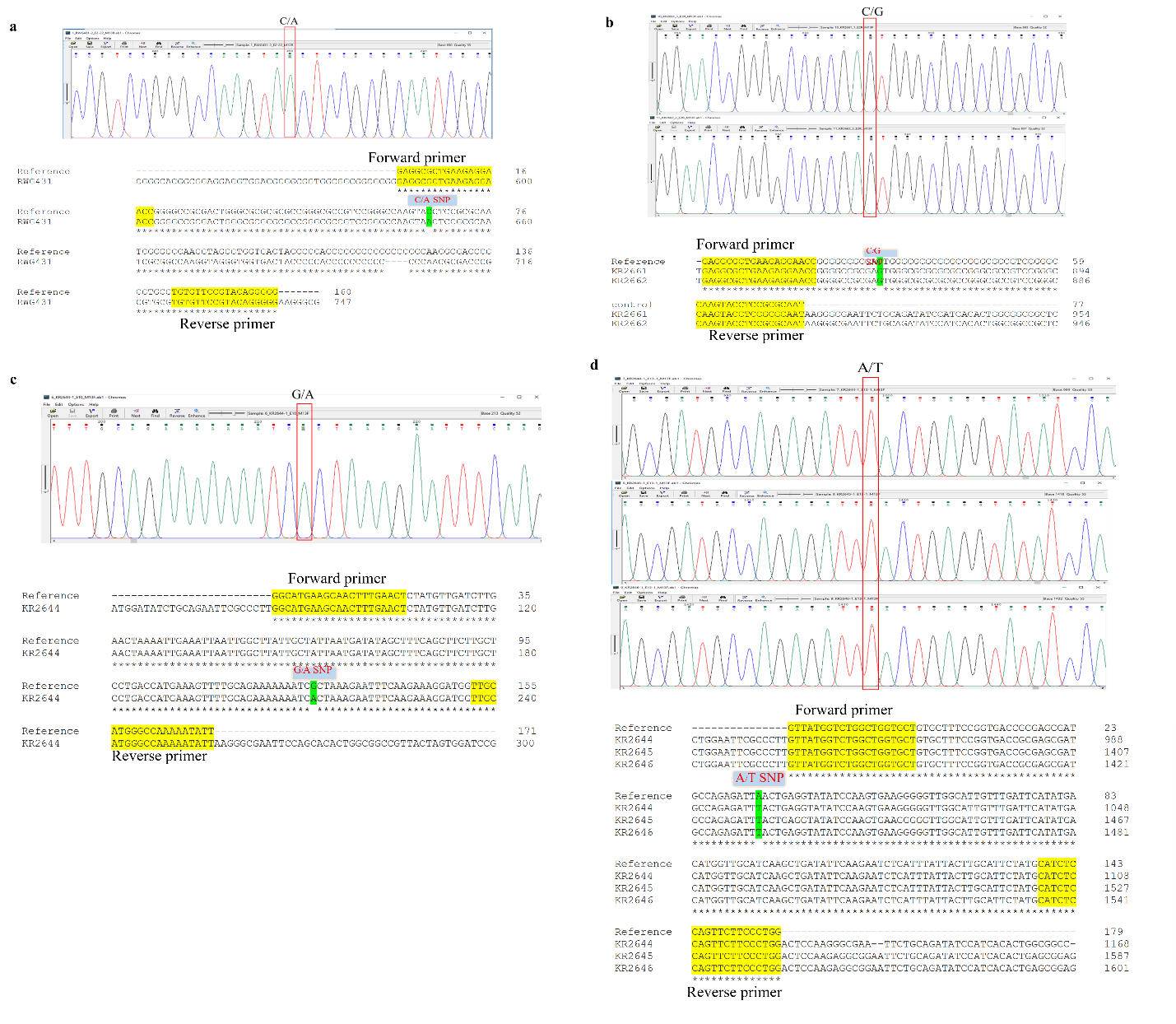


**Fig. S5.** Multiple sequence alignment (MSA) for valiadation of novel functional alleles from different accessions with reference sequence. **a,** MSA for *BADH2-E2-476C>A* from RWG-431. **b,** MSA for *BADH2-E2-440C>G* from RWG-475 and RWG-476 **c,** MSA for *BADH2-*E10-4460G>A from RWG-459. **d,** MSA for *BADH2-*E13-5433A>T RWG-459 from RWG-459, RWG-460, and RWG-461.


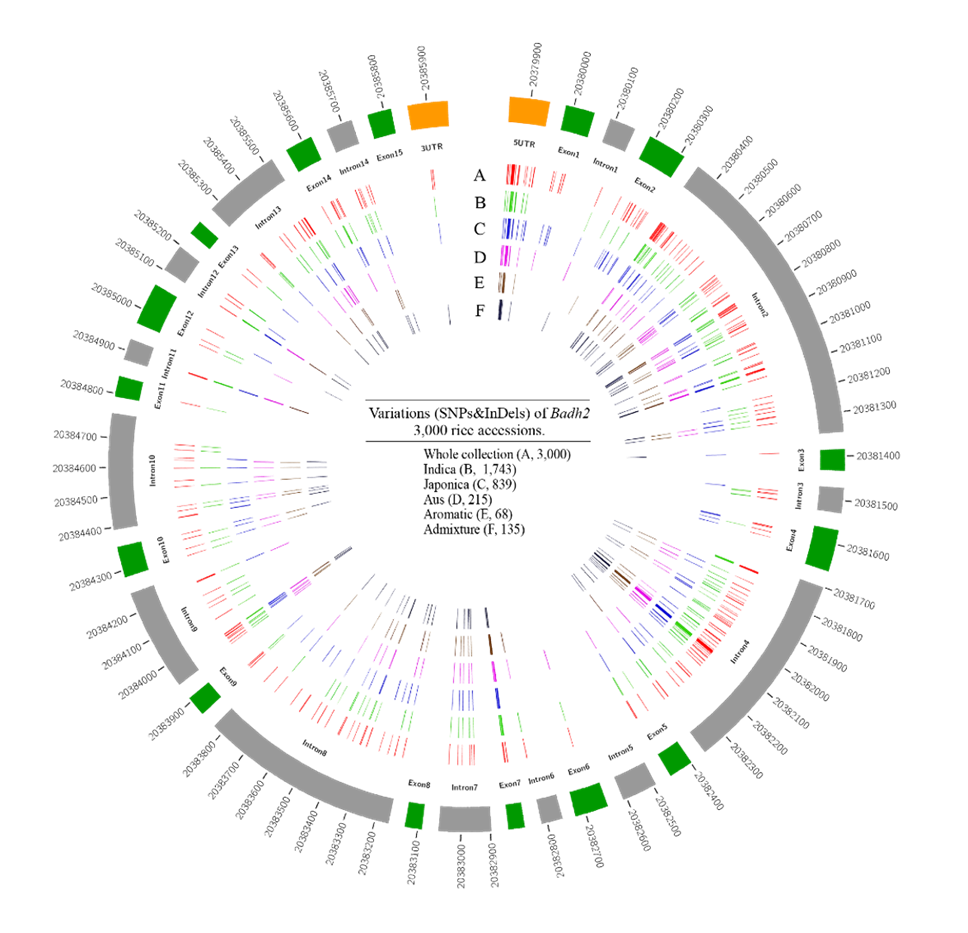


**Fig. S6.** Distribution of the nucleotide variations (SNPs and indels) in *BADH2* gene 3K rice accessions**.** (**A-G**) Highlights SNP/indel positions. (**A**) Total polymorphisms detected in 3,000 accessions, (**B**) Polymorphisms in the rice of indica type, (**C**) Polymorphisms in japonica type, (**D**) Polymorphisms in aus type, (**E**) Polymorphisms in aromatic type, (**F**) Polymorphisms in admixture type, (**G**) Polymorphisms in wild rice.


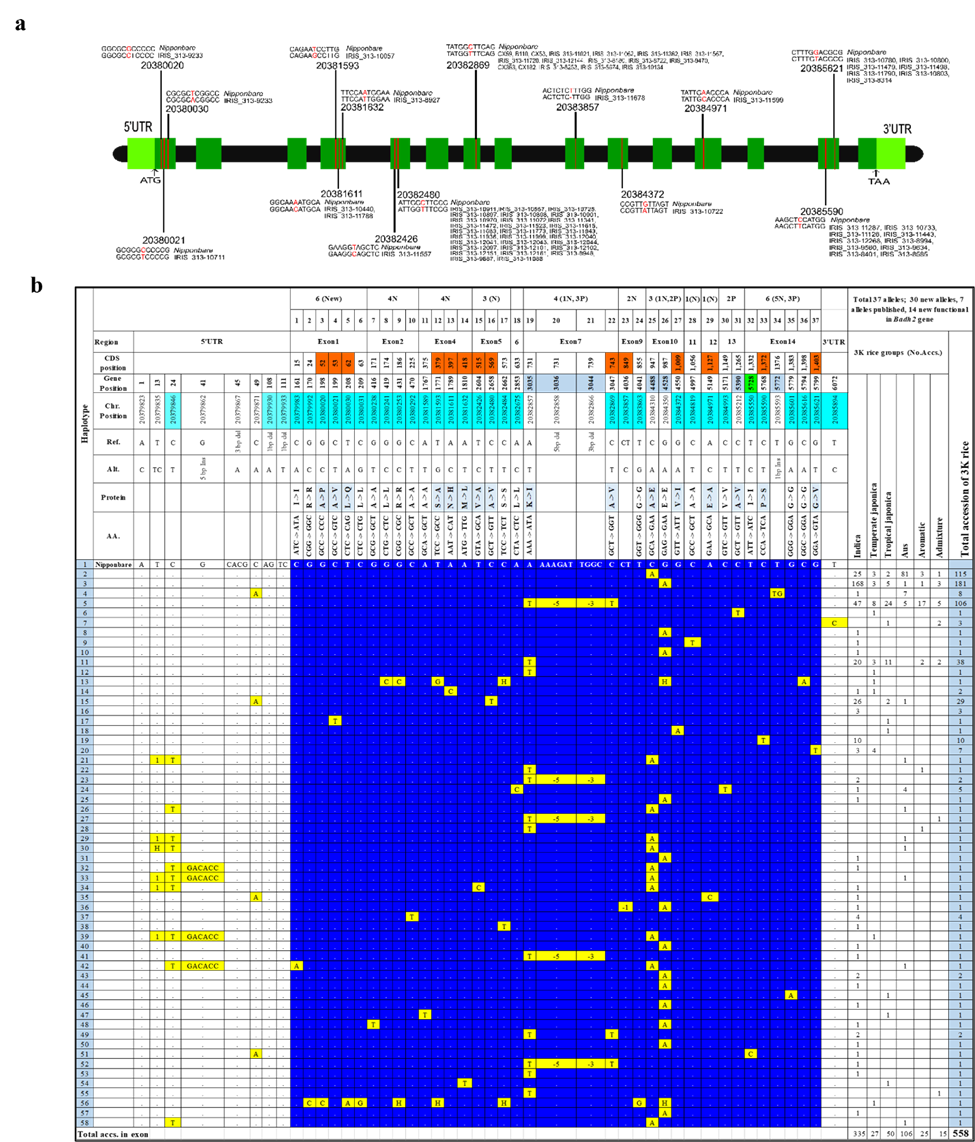


**Fig. S7.** The allellic polymorphism and haplotype analysis of the *BADH*2 coding region from 3K rice accessions. (a) The novel 14 SNPs and indels positions. (b) Fifty-eight haplotypes generated based on *BADH2* coding region from 3,000 rice accessions.


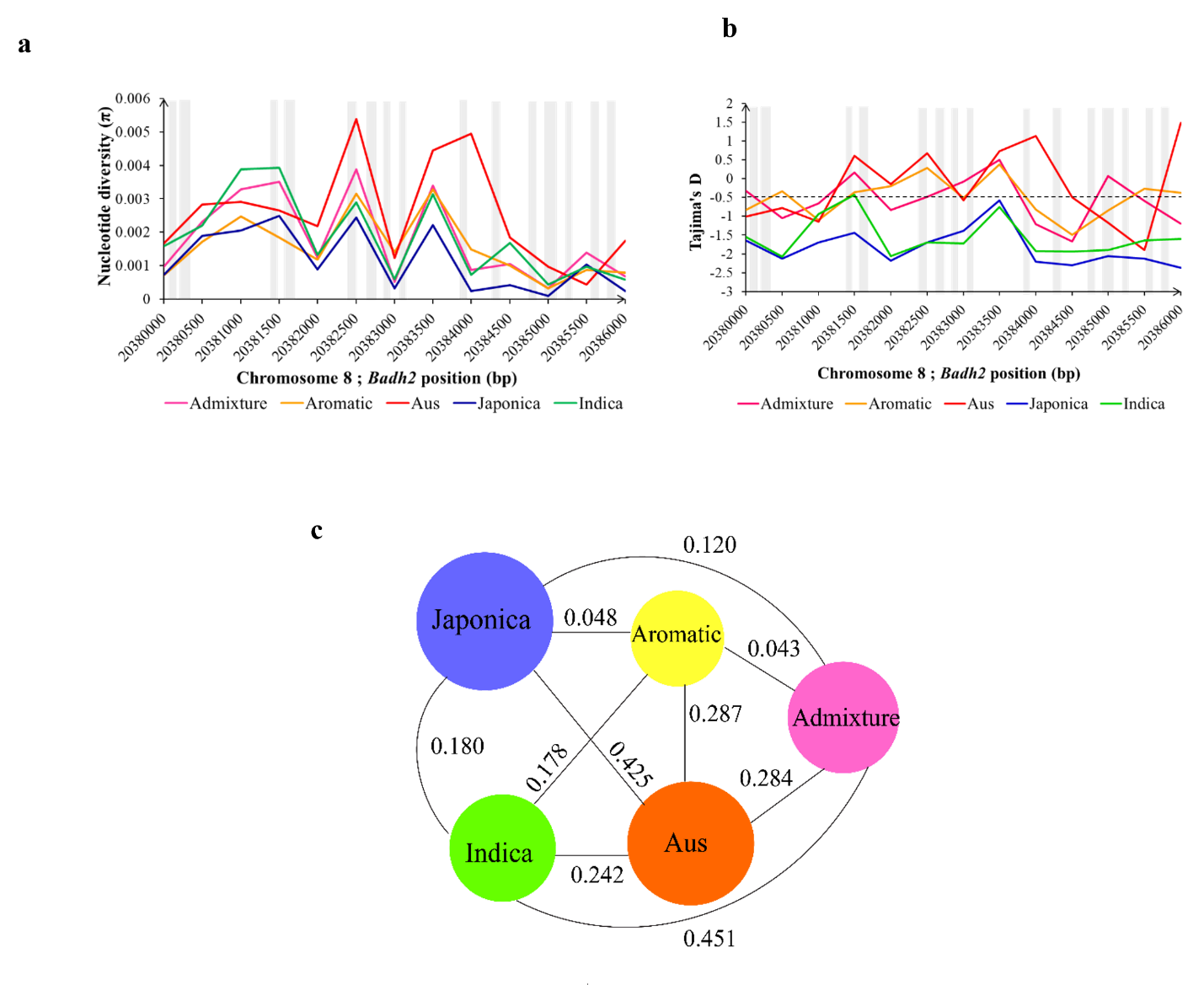


**Fig. S8** Genetic diversity indices based *BADH*2 gene of 3K rice accessions. **a,** Nucleotide diversity (π) with a 500-slide window. **b**, Tajima’s D with a 500-slide window. **c,** The *Fst* between different groups. The circle size demonstrated the levels of nucleotide diversity value.


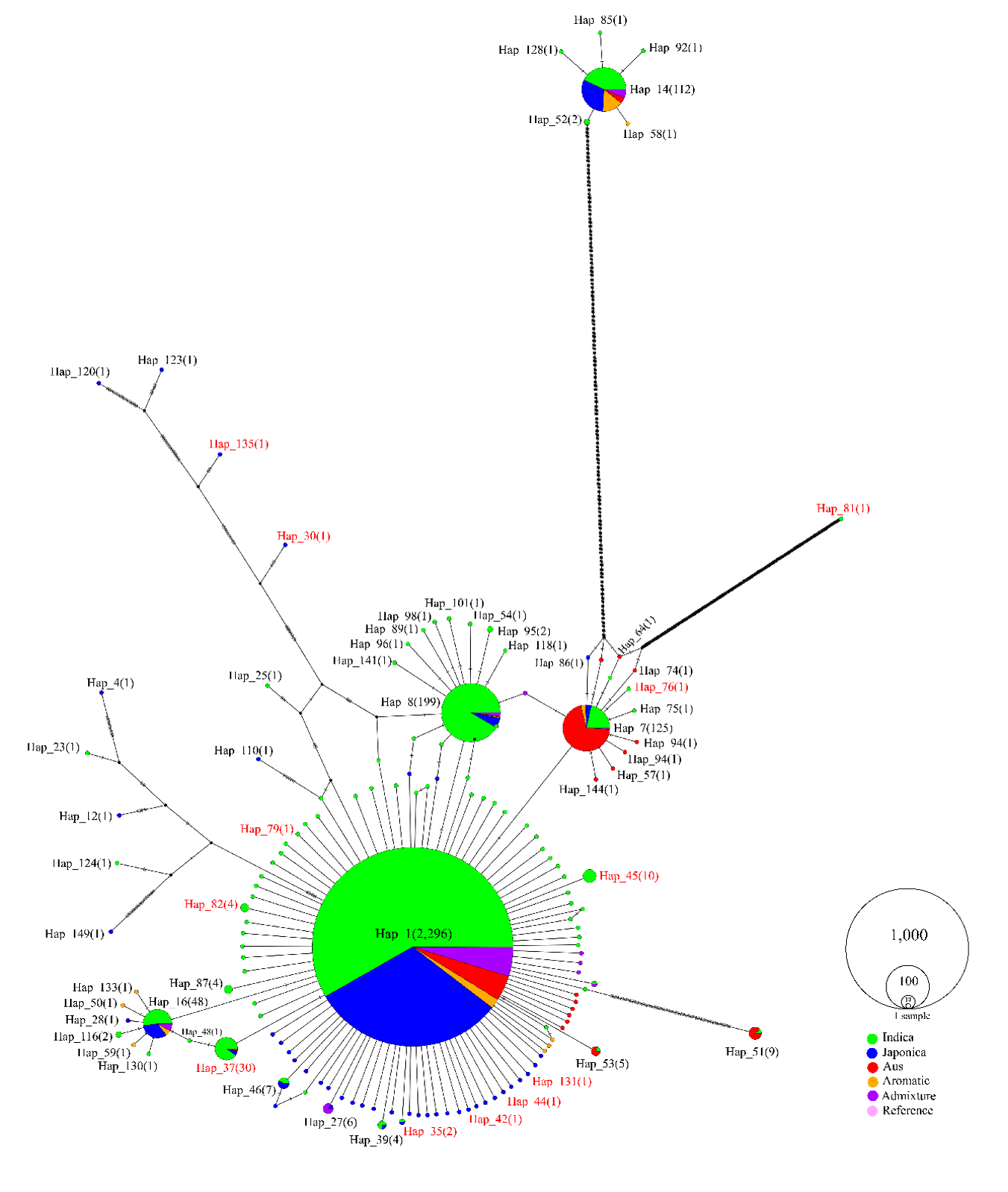


**Fig. S9.** Haplotype network of *BADH*2 gene from 3K rice accessions. Circle size is proportional to the number of samples and ecotypes, and dashes between haplotype represent mutational steps between alleles.


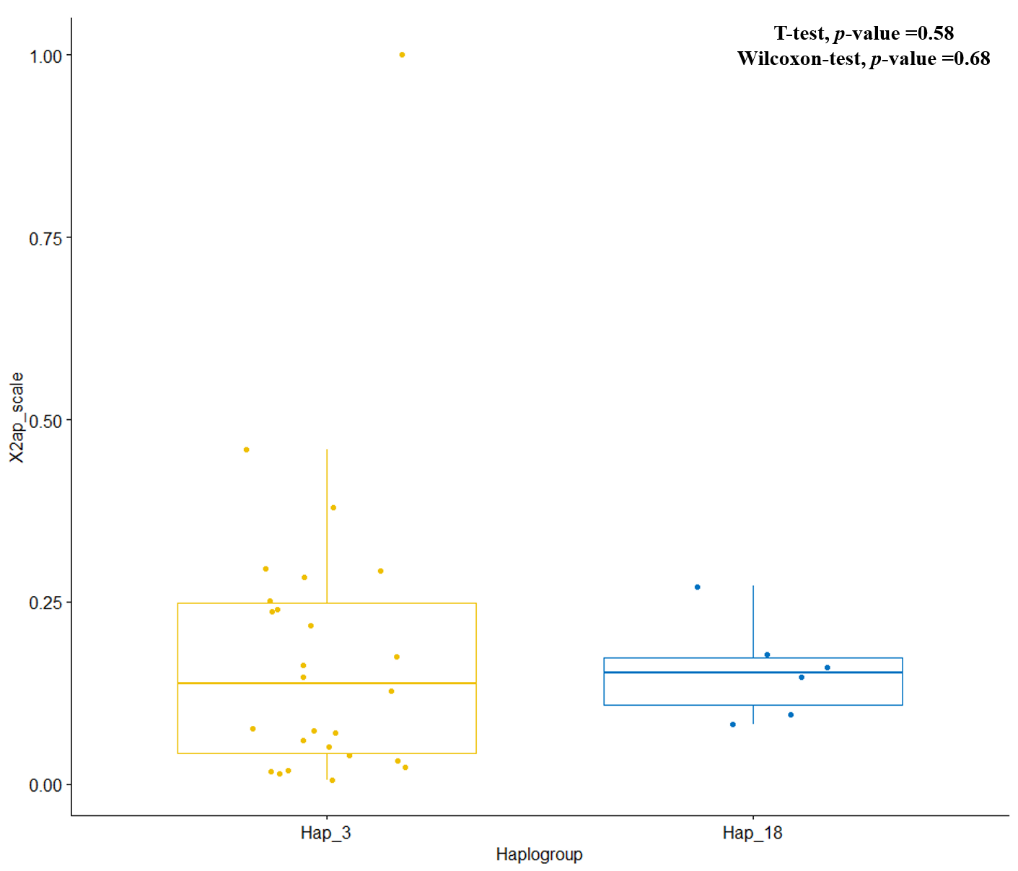


**Fig. S10.** Comparison of 2AP contents in two major haplotypes using the T‐test and Wilcoxon test. There is no significant difference between the two groups (Hap3 and Hap18).

**Supplementary References**

[1] Löytynoja A, Goldman N. Phylogeny-aware gap placement prevents errors in sequence alignment and evolutionary analysis. Science 2008;320:1632–5.

[2] Sood BC, Siddiq. A rapid technique for scent determination in rice [India]. Indian J Genet Plant Breed 1978.

[3] Lestari AP, Abdullah B, Junaedi A, Aswidinnoor H. PERFORMANCE OF GRAIN QUALITY AND AROMA OF AROMATIC NEW PLANT TYPE PROMISING RICE LINES. Indones J Agric Sci 2011;12:83–93. https://doi.org/10.21082/ijas.v12n2.2011.p83-93.

[4] Trung KH, Nguyen TK, Khuat HBT, Nguyen TD, Khanh TD, Xuan TD, et al. Whole genome sequencing reveals the islands of novel polymorphisms in two native aromatic japonica rice landraces from Vietnam. Genome Biol Evol 2017;9:1816–20.

[5] Bradbury LM, Fitzgerald TL, Henry RJ, Jin Q, Waters DL. The gene for fragrance in rice. Plant Biotechnol J 2005;3:363–70.

[6] Shi W, Yang Y, Chen S, Xu M. Discovery of a new fragrance allele and the development of functional markers for the breeding of fragrant rice varieties. Mol Breed 2008;22:185–92.

[7] He Q, Yu J, Kim T-S, Cho Y-H, Lee Y-S, Park Y-J. Resequencing reveals different domestication rate for BADH1 and BADH2 in rice (Oryza sativa). PloS One 2015;10:e0134801.

[8] Shao G, Tang S, Chen M, Wei X, He J, Luo J, et al. Haplotype variation at Badh2, the gene determining fragrance in rice. Genomics 2013;101:157–62.

[9] Kovach MJ, Calingacion MN, Fitzgerald MA, McCouch SR. The origin and evolution of fragrance in rice (Oryza sativa L.). Proc Natl Acad Sci 2009;106:14444–9.

[10] Dissanayaka S, Kottearachchi NS, Weerasena J, Peiris M. Development of a CAPS marker for the badh2. 7 allele in S ri L ankan fragrant rice (O ryza sativa). Plant Breed 2014;133:560–5.

[11] Hashemi FSG, Rafii MY, Ismail MR, Mahmud TMM, Rahim HA, Asfaliza R, et al. Biochemical, Genetic and Molecular Advances of Fragrance Characteristics in Rice. Crit Rev Plant Sci 2013;32:445–57. https://doi.org/10.1080/07352689.2013.807716.

[12] Min M-H, Maung TZ, Cao Y, Phitaktansakul R, Lee G-S, Chu S-H, et al. Haplotype Analysis of BADH1 by Next-Generation Sequencing Reveals Association with Salt Tolerance in Rice during Domestication. Int J Mol Sci 2021;22:7578.

[13] Fitzgerald TL, Waters DLE, Henry RJ. The effect of salt on betaine aldehyde dehydrogenase transcript levels and 2-acetyl-1-pyrroline concentration in fragrant and non-fragrant rice (Oryza sativa). Plant Sci 2008;175:539–46. https://doi.org/10.1016/j.plantsci.2008.06.005.

[14] He Q, Park Y-J. Discovery of a novel fragrant allele and development of functional markers for fragrance in rice. Mol Breed 2015;35:1–10.
